# Target mechanisms of mindfulness-based programmes and practices: a scoping review

**DOI:** 10.1136/bmjment-2023-300955

**Published:** 2024-08-24

**Authors:** Shannon Maloney, Merle Kock, Yasmijn Slaghekke, Lucy Radley, Alba Lopez-Montoyo, Jesus Montero-Marin, Willem Kuyken

**Affiliations:** 1 Department of Psychiatry, University of Oxford, Oxford, Oxfordshire, UK; 2 Centre for the Psychology of Learning and Experimental Psychopathology, KU Leuven, Leuven, Flanders, Belgium; 3 Department of Experimental Psychology, University of Oxford, Oxford, UK; 4 Universitat Jaume I, Castello de la Plana, Comunitat Valenciana, Spain; 5 Teaching, Research & Innovation Unit, Parc Sanitari Sant Joan de Déu, Sant Boi de Llobregat, Spain; 6 Consortium for Biomedical Research in Epidemiology & Public Health (CIBER Epidemiology and Public Health - CIBERESP), Madrid, Spain

**Keywords:** Depression, Adult psychiatry, Depression & mood disorders

## Abstract

**Question:**

Mindfulness-based programmes (MBPs) and practices have demonstrated effects in mental health and well-being, yet questions regarding the target mechanisms that drive change across the population remain unresolved.

**Study selection and analysis:**

Five databases were searched for randomised controlled trials that evaluate the indirect effects (IEs) of an MBP or mindfulness practice in relation to mental health and well-being outcomes through psychological mechanisms.

**Findings:**

27 eligible studies were identified, with only four exploring mechanisms in the context of specific mindfulness practices. Significant IEs were reported for mindfulness skills, decentering and attitudes of mindfulness (eg, self-compassion) across different outcomes, population samples, mental health strategies and active comparators. Evidence gap maps and requirements for testing and reporting IEs are provided to help guide future work.

**Conclusions:**

Mindfulness skills, decentering and attitudes of mindfulness may be key intervention targets for addressing the mental health of whole populations. However, future work needs to address significant knowledge gaps regarding the evidence for alternative mechanisms (eg, attention and awareness) in relation to unique outcomes (eg, well-being), mental health strategies (ie, promotion) and active comparators. High-quality trials, with powered multivariate mediation analyses that meet key requirements, will be needed to advance this area of work.

**Trial registration number:**

10.17605/OSF.IO/NY2AH.

**WHAT IS ALREADY KNOWN ON THIS TOPIC**

- Mindfulness-based programmes (MBPs) and practices have demonstrated effects in mental health and well-being, yet questions regarding the target mechanisms that drive change across the population remain unresolved.

**WHAT THIS STUDY ADDS**

- This review aims to build on past reviews by (1) summarising the evidence for potential mechanisms underlying mindfulness practices as one key subcomponent of MBPs; (2) prioritising high quality and established formal analytical methods of mediation; and (3) examining the evidence for mechanisms in relation to outcomes that map across the population.

**HOW THIS STUDY MIGHT AFFECT RESEARCH, PRACTICE OR POLICY**

- The hope is that the findings of this review will help inform future work and provide novel insight into the broader application of MBPs and the potential mechanisms responsible for shifting more of the population towards the ‘highs’ and away from the ‘lows’.

## Background

 It has been estimated that around one in every eight individuals have a mental health condition and its attribution to total disease burden continues to rise on a global scale.^1^ Mental health conditions concern a broad range of disorders, psychosocial disabilities and mental states of distress. Mental health, on the other hand, refers to the capacity of thought, emotion and behaviour that allows an individual to realise their own potential and positively contribute to their community. It exists on a continuum and, therefore, concerns the entire population.^1^ Mental health approaches, which solely address those with mental health conditions (ie, *high-risk strategies*, eg, treatment), leave a larger percentage of the population susceptible to entering ill health without intervention. To address this concern, global mental health agendas have recognised the importance of *population-based strategies*, which aim to address the entire population distribution from the ‘lows’ (eg, poor mental health, low well-being) to the ‘highs’ (eg, good mental health, high well-being) and ultimately shift this distribution in a more positive direction.

Mindfulness-based programmes (MBPs) may serve as one potential pathway to help improve mental health and well-being across a wider distribution of the population. MBPs align with broader global mental health objectives by prioritising prevention to reduce the development of mental health conditions, building resilience in individuals and communities and recognising the importance of addressing mental health on a continuum (eg, ‘the lows’ to ‘the highs’).^2^ MBPs were introduced in mainstream settings when mindfulness-based stress reduction (MBSR) was developed to help people with physical health conditions manage symptoms, such as chronic pain.^3^ Mindfulness-based cognitive therapy (MBCT), an adaptation which includes psychoeducation elements from cognitive–behavioural therapy (CBT), was developed to help prevent depressive relapse.^4^ MBSR/MBCT are traditionally formatted as 8-week programmes with weekly group-based sessions, led by a trained mindfulness instructor, and include daily self-led home-based mindfulness practice. These programmes have demonstrated effectiveness in those with recurrent depression (MBCT) and chronic pain (MBSR).^5 6^ There is also a growing body of evidence that has demonstrated that mindfulness interventions can help a wider distribution of the population experience improvements in mental well-being.^7^ However, more work is needed to further understand effectiveness, and the mechanisms of action of MBPs within this broader global mental health approach.

Theoretical frameworks^8^ suggest that the model of change for MBPs is driven by the cultivation of mindfulness skills. Mindfulness, as a multidimensional construct, involves paying attention to the present moment experience with attitudes such as self-compassion, curiosity, kindness and care. It enables people to take a wider perspective (sometimes called decentering or meta-awareness) and see both internal and external stimuli as temporary events. The theory of MBCT and MBSR postulates that through an increase in mindfulness skills individuals with recurrent depression (MBCT) and chronic pain (MBSR), respectively, can attend to their internal experiences (eg, thoughts, feelings, bodily sensations) without judgement—allowing them to see these experiences more objectively (ie, decentering or meta-awareness) and as temporary which can help reduce maladaptive strategies (eg, rumination, reactivity) which exacerbate symptoms. Past reviews have examined mechanisms of change underlying MBPs in the context of more clinical or at-risk populations (eg, individuals with recurrent depression or psychological and physical conditions) and found consistent evidence for mindfulness skills in relation to clinical outcomes.^9 10^ Additional reviews^11 12^ have investigated mechanisms of MBPs across a wider distribution of the population (eg, clinical and non-clinical samples) and found consistent evidence for mindfulness skills and reactivity (eg, emotional, cognitive) in relation to mental health outcomes (eg, anxiety, depression, psychological distress, stress, negative affectivity). However, these reviews do not evaluate the quality of mediation testing and reporting in alignment with emerging mediation frameworks.^13^ In light of the growing evidence base for MBPs and their efficacy across a wider distribution of the population and in relation to outcomes pertaining to the positive valence system (eg, mental well-being),^714^,^18^ an updated review that comprehensively maps out the evidence for potential mechanisms that may be shared across and unique to different population samples and outcomes is needed. There is also an argument for investigating mechanisms in the context of independent components of MBPs (ie, individual mindfulness practices) to further understand active ingredients. Past reviews have demonstrated that stand-alone mindfulness practices produce change in outcomes relating to symptoms of depression, anxiety and stress with small-to-moderate effects.^19 20^ Moreover, there is a growing body of evidence that has examined potential mechanisms of individual practices and practice modules.^21 22^ However, this area of research is underdeveloped and warrants further investigation.

## Objective

The primary aim of this review is to summarise the evidence for target mechanisms, underlying MBPs and mindfulness practices, which may be shared across or unique to different population samples and outcomes. This review aims to build on past reviews by (1) summarising the evidence for potential mechanisms underlying mindfulness practices as one key subcomponent of MBPs, (2) prioritising high quality and established formal analytical methods of mediation and (3) examining the evidence for mechanisms in relation to outcomes that map across the population. The hope is that the findings of this review will help inform future work and provide novel insight into the broader application of MBPs and the potential mechanisms responsible for shifting more of the population towards the ‘highs’ and away from the ‘lows’.

## Study selection and analysis

A scoping review approach was implemented to allow for an iterative and exploratory focus to assess the broad research question, to map the available evidence for mediation in relation to emerging frameworks for testing and reporting the indirect effect (IE) and to map key conceptual terms for mechanisms and outcomes. Several frameworks^23 24^ were used as guidance and the reporting follows the checklist for Preferred Reporting Items for Systematic reviews and Meta-Analyses (PRISMA) extension for Scoping Reviews.^25^ This review was preregistered with Open Science Framework (OSF) on 16 July 2020 (DOI: 10.17605/OSF.IO/XJDSU). A pre-print protocol was made available on 14 September 2020, outlining the research questions, methods and data synthesis strategy. The initial search was run on 13 December 2020, with additional searches run on 25 July 2022, 20 December 2022, 3 July 2023 and 7 March 2024. An addendum to the preregistration form was submitted to OSF on 3 November 2021 (DOI: 10.17605/OSF.IO/NY2AH) and an updated protocol was uploaded to OSF on 1 April 2023 (see online supplemental 1). The timeline for these updates is summarised in online supplemental 2.

Five electronic databases (PubMed, PsycINFO, Embase, Scopus and Cochrane Central) were searched for relevant records up until 7 March 2024. The search string was developed by first breaking down the research question (‘Through which mediators and mechanisms do MBPs and mindfulness practices produce change in the adult population’) by population (ie, relevant characteristics of participant sample; eg, entire adult population distribution), context (ie, the setting or discipline; eg, mindfulness) and concept terms (ie, research designs, frameworks, theories; eg, mediation).^26^ Synonyms and related terms were then generated. The search string was refined by running a mock search in PubMed and consulting experts in the field, such as mindfulness teachers and a research librarian. Key terms in the search string included ‘mechanism*’, ‘mediat*’, ‘*mindfulness*’ and ‘*mindfulness-based*’. Specific practice terms included: ‘*body scan*’, ‘*mindful movement*’ and ‘*yoga*’. The search string used for each database is provided in online supplemental 3. Other sources (eg, Web of Science conference proceedings, PsycEXTRA and connectedpapers.com) were searched for grey literature up until 7 March 2024. The snowballing method was also implemented to identify additional records from the reference lists of the included papers (online supplemental 4) and key systematic reviews.^9^,^12^

The inclusion and exclusion criteria reflect the population, context and concept.^26^ In terms of the population, we included any study concerning an adult sample (aged 18 and above) to reflect the entire adult population distribution (from mental health conditions to well-being). For the context, eligible studies evaluated an MBP defined by Crane *et al*^27^ or a formal mindfulness practice that, in the context of an MBP, is home-based, scheduled with audio guidance and practised at least three times per week. This criterion was developed from a content analysis of four key MBP curricula (online supplemental 5). For the concept, we included studies that evaluated the IEs of an MBP or formal mindfulness practice (independent variable) on mental health and well-being outcomes (dependent variable) through psychological mediators (mechanism) (see online supplemental 6 for a visual depiction of the IE). The evaluation of the IE followed recommendations outlined by Zhao *et al*,^13^ which establish five categories of mediation (complementary, competitive, indirect-only, direct-only effect or no effect). For more information regarding these criteria, see online supplemental 7. Only studies that implemented a randomised controlled trial (RCT) design with a control group that had active ingredients were included to capture the highest-quality evidence. The comparator was loosely defined as including ‘active’ ingredients if it included more than the mere passage of time (eg, wait list control group). For more details on the inclusion and exclusion criteria, see online supplemental 8.

Each record was screened by at least two independent authors for titles and abstracts (SM, MK, YS, JM-M and LR) and full texts (SM, MK, YS and AL-M). Following Siddaway *et al*, data extraction was piloted and completed by one author (SM), with 15% of the output randomly cross-checked by a second author (YS, MK and JM-M).^28^ Any disagreements were resolved by a third author. For records that did not have the full text, corresponding authors were contacted. The following information was piloted and then extracted for charting: (1) author(s) and year of publication, (2) sample characteristics, (3) type of mental health strategy (ie, treatment, prevention or promotion), (4) type of MBP or formal mindfulness practice, (5) dosage, (6) delivery mode (eg, online or face to face), (7) type of active comparator(s), (8) reported mechanisms, (9) reported outcomes, (10) data analytic approach for testing mediation and (11) key findings. A narrative synthesis was conducted whereby evidence gap maps help elucidate how and why MBPs and practices may work in relation to different outcomes, mental health strategies (eg, population samples) and active comparators. Further details can be found in online supplemental materials on the evidence gap map methodology (online supplemental 9) and conceptual mapping of key terms (ie, outcomes, mechanisms, mental health strategies) (online supplemental 10 and 11). Quality assessments for testing and reporting IEs are provided in online supplemental 12.

## Findings

**Figure 1:**
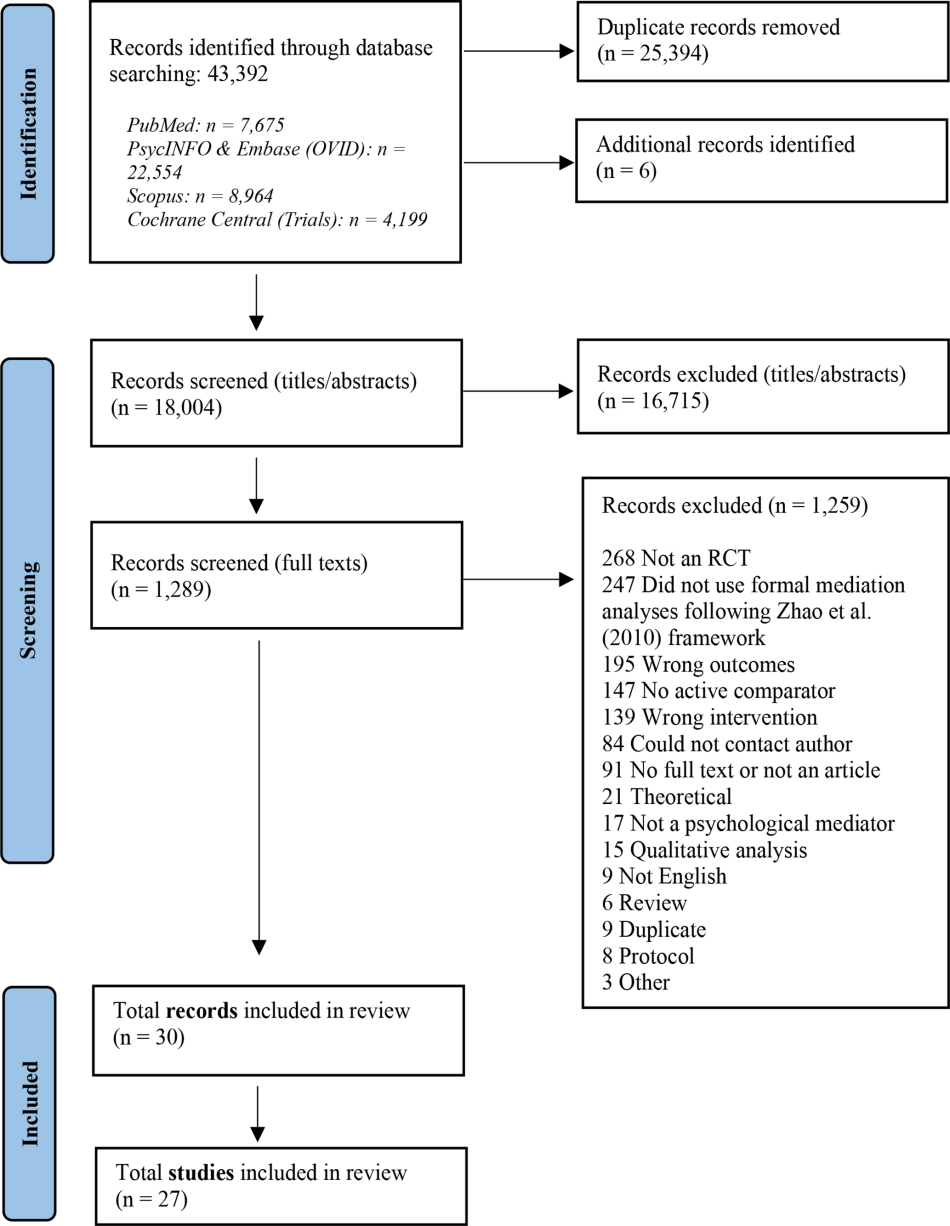
PRISMA flow chart. The PRISMA flow diagram of the records initially identified from the database search before and after deduplication is illustrated. The chart also outlines the records screened at the title and abstract stage as well as the full-text stage, including the number of records removed after full-text screening with the reasons specified. The additional records found outside of the search (n=6) were identified from either forward or backward citation searching of the included records and relevant reviews, from grey literature sources or from corresponding authors who shared additional records. Out of the 30 records included, 3 records shared the same sample with another record and therefore there were 27 studies included in total. Note that both English and Spanish publications were considered, given the primary languages of the research team. However, all included studies were published in English and, therefore, no Spanish publications met the inclusion criteria. RCT, randomised controlled trial.

The PRISMA flow chart shows 43 392 records identified across the 5 electronic databases. Before the records were screened for eligibility, 25 394 records were removed as duplicates. Six additional records were identified from either snowballing included papers or past reviews, searching grey literature sources or corresponding authors sharing additional papers. For title and abstract screening, 18 004 records were reviewed by two independent referees, with 16 715 excluded, leaving 1289 records for full-text screening. A total of 30 records were included in the review with 3 records sharing the same sample with another record, leaving 27 eligible studies (figure 1).

General findings, regarding the scope of the evidence, indicate four eligible studies that evaluated a formal mindfulness practice (1 body scan, 1 mindfulness of emotions, 1 breath and 1 body scan and breath) and 23 studies that evaluated an MBP. In terms of outcomes, 11 studies examined outcomes related to mental health conditions, 16 studies examined outcomes related to mental languishing and 9 studies examined outcomes related to well-being. 11 studies examined an MBP or practice as a treatment strategy (10=MBP, 1=practice), 16 as a prevention strategy (14=MBP, 2=practice) and 9 as a promotion strategy only (8=MBP, 1=practice). For the 16 studies that implemented a preventive strategy, 4 examined universal prevention, none examined indicated prevention only, 6 explored selective prevention only and 6 studies examined elements of both indicated and selective prevention. The majority of the included studies compared an MBP to treatment as usual (TAU) (n=13). Five studies compared an MBP or practice to stress management or relaxation (SM/R), three studies compared an MBP to CBT or cognitive interventions (CBT/CI), four studies compared an MBP or practice to attentional control (AC) and three studies compared an MBP to self-help mindfulness (SH-M).

**Figure 2:**
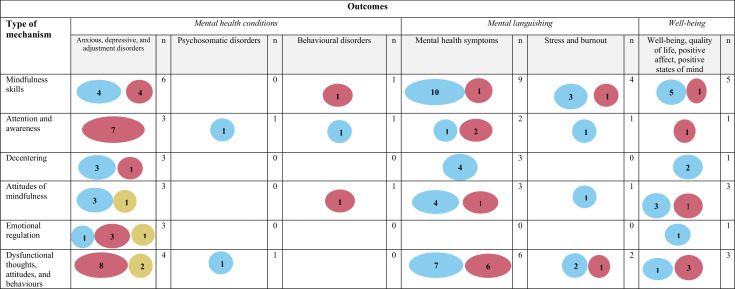
The evidence gap map for mechanisms of MBP and practices split by outcomes (mental health conditions: anxious, depressive and adjustment disorders; psychosomatic disorders; behavioural disorders; mental languishing: mental health, and stress and burnout; and well-being: positive affect, positive states of mind, quality of life and well-being) is illustrated. This evidence map reflects the key findings (related to the indirect effects in univariate mediation models) reported in online supplemental materials. Indirect effects (IEs) in multivariate mediation models are included in online supplemental materials. The IEs in the context of univariate models are prioritised in the evidence maps for conceptual clarity as multivariate models demonstrate the extent to which the mediated effect is dependent on the interaction between multiple mediators and the extent to which some make significant independent contributions, which are nuances that could not be captured here but are provided in online supplemental materials. For studies that reported total scores and subscale scores, total scores were prioritised. For studies whereby the proposed mechanisms were explored pre–post and pre follow-up, the pre–post results were prioritised. Online supplemental materials show the list of mechanism categories (mindfulness skills, attention and awareness, decentering, attitudes of mindfulness, emotional regulation, and dysfunctional thoughts, attitudes, and behaviours) and outcome categories and provides a working definition for each category and the full list of measures (with item examples) from the included papers that map onto each category. The total number of non-significant and significant indirect effects are reported, along with significant opposite direction findings. The size of the circle corresponds to the number of findings with larger circles signifying more findings. The opposite direction findings are indicated when the results provided support for the active comparator (eg, cognitive–behavioural therapy) rather than for the mindfulness condition. To help isolate mechanisms of change, and in light of the limited evidence base for individual mindfulness practices, the evidence gap maps combined the evidence across MBPs and practices. Note that multiple findings can be reported from one study. Studies therefore had multiple aims or addressed multiple outcomes that fell under different categories. 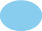
=Significant findings; 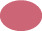
=Non-significant findings; 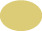
=Significant findings in opposite direction; 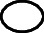
=Less evidence; 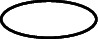
=more evidence. N, number of studies.

**Figure 3:**
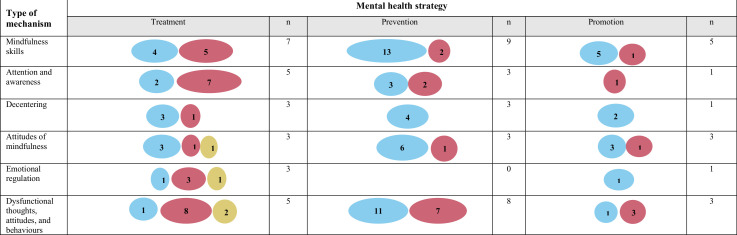
Evidence map for mechanisms of MBP and practices split by mental health strategy (treatment, prevention and promotion) is provided. The different types of mental health strategies are reported in online supplemental materials. This evidence map reflects the key findings (related to the indirect effects in univariate mediation models) reported in online supplemental materials. Indirect effects (IEs) in multivariate mediation models are included in online supplemental materials. The IEs in the context of univariate models are prioritised in the evidence maps for conceptual clarity as multivariate models demonstrate the extent to which the mediated effect is dependent on the interaction between multiple mediators and the extent to which some make significant independent contributions, which are nuances that could not be captured here but are provided in online supplemental materials. For studies that reported total scores and subscale scores, total scores were prioritised. For studies whereby the proposed mechanisms were explored pre–post and pre follow-up, the pre–post results were prioritised. Online supplemental materials shows the list of mechanism and outcome categories and also provides a working definition for each category and the full list of measures (with item examples) from the included papers that map onto each category. The total number of non-significant and significant indirect effects are reported, along with significant opposite direction findings. The size of the circle corresponds to the number of findings with larger circles signifying more findings. The opposite direction findings are indicated when the results provided support for the active comparator (eg, cognitive–behavioural therapy) rather than for the mindfulness condition. To help isolate mechanisms of change, and in light of the limited evidence base for individual mindfulness practices, the evidence gap maps combined the evidence across MBPs and practices. Note that multiple findings can be reported from one study. 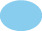
=Significant findings; 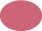
=Non-significant findings; 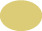
=Significant findings in opposite direction; 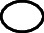
=Less evidence; 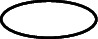
=more evidence. N, number of studies.

**Figure 4:**
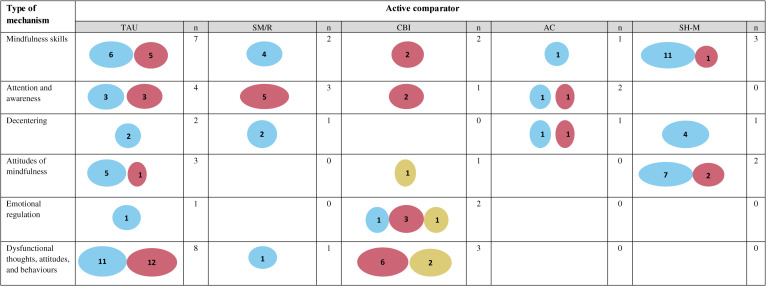
The evidence gap map for mechanisms of MBPs and practices split by type of active comparator is illustrated. Five categories for different types of active comparators were identified: treatment as usual (TAU), stress management and relaxation (SM/R), cognitive behavioural therapy/cognitive interventions (CBT/CI), attentional control (AC) and self-help mindfulness (SH-M). This evidence map reflects the key findings (related to the indirect effects in univariate mediation models) reported in online supplemental materials. Indirect effects (IEs) in multivariate mediation models are included in online supplemental materials. The IEs in the context of univariate models are prioritised in the evidence maps for conceptual clarity as multivariate models demonstrate the extent to which the mediated effect is dependent on the interaction between multiple mediators and the extent to which some make significant independent contributions, which are nuances that could not be captured here but are provided in online supplemental materials. For studies that reported total scores and subscale scores, total scores were prioritised. For studies whereby the proposed mechanisms were explored pre–post and pre follow-up, the pre–post results were prioritised. Online supplemental materials show the list of mechanism and outcome categories and also provides a working definition for each category and the full list of measures (with item examples) from the included papers that map onto each category. The total number of non-significant and significant indirect effects are reported, along with significant opposite direction findings. The size of the circle corresponds to the number of findings with larger circles signifying more findings. The opposite direction findings are indicated when the results provided support for the active comparator (eg, cognitive–behavioural therapy) rather than for the mindfulness condition. To help isolate mechanisms of change, and in light of the limited evidence base for individual mindfulness practices, the evidence gap maps combined the evidence across MBPs and practices. Note that multiple findings can be reported from one study. 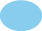
=Significant findings; 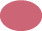
=Non-significant findings; 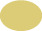
=Significant findings in opposite direction; 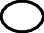
=Less evidence; 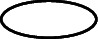
=Less evidence. N, number of studies.

Evidence gap maps were generated to summarise the number of significant, non-significant and opposite direction IEs by outcome (mental health conditions, mental languishing, well-being), type of mental health strategy (treatment, prevention, promotion) and type of active comparator (TAU, SM/R, CBT/CI, AC and SH-M). The largest proportion of significant IEs to non-significant or opposite direction IEs was demonstrated for mindfulness skills, decentering and attitudes of mindfulness in relation to mental languishing outcomes; with mixed findings in the context of outcomes pertaining to mental health conditions; and preliminary evidence in the context of well-being (figure 2). With the evidence by mental health strategy, the largest proportion of significant IEs was found for mindfulness skills, decentering, and attitudes of mindfulness in the context of prevention; with mixed evidence in the context of treatment; and preliminary evidence in the context of promotion (figure 3). The most commonly used active comparator was TAU, whereby there were significant IEs for mindfulness skills, decentering and attitudes of mindfulness in the context of the mindfulness condition but not TAU (figure 4). Online supplemental 13 provides additional information regarding key findings.

**Figure 5:**
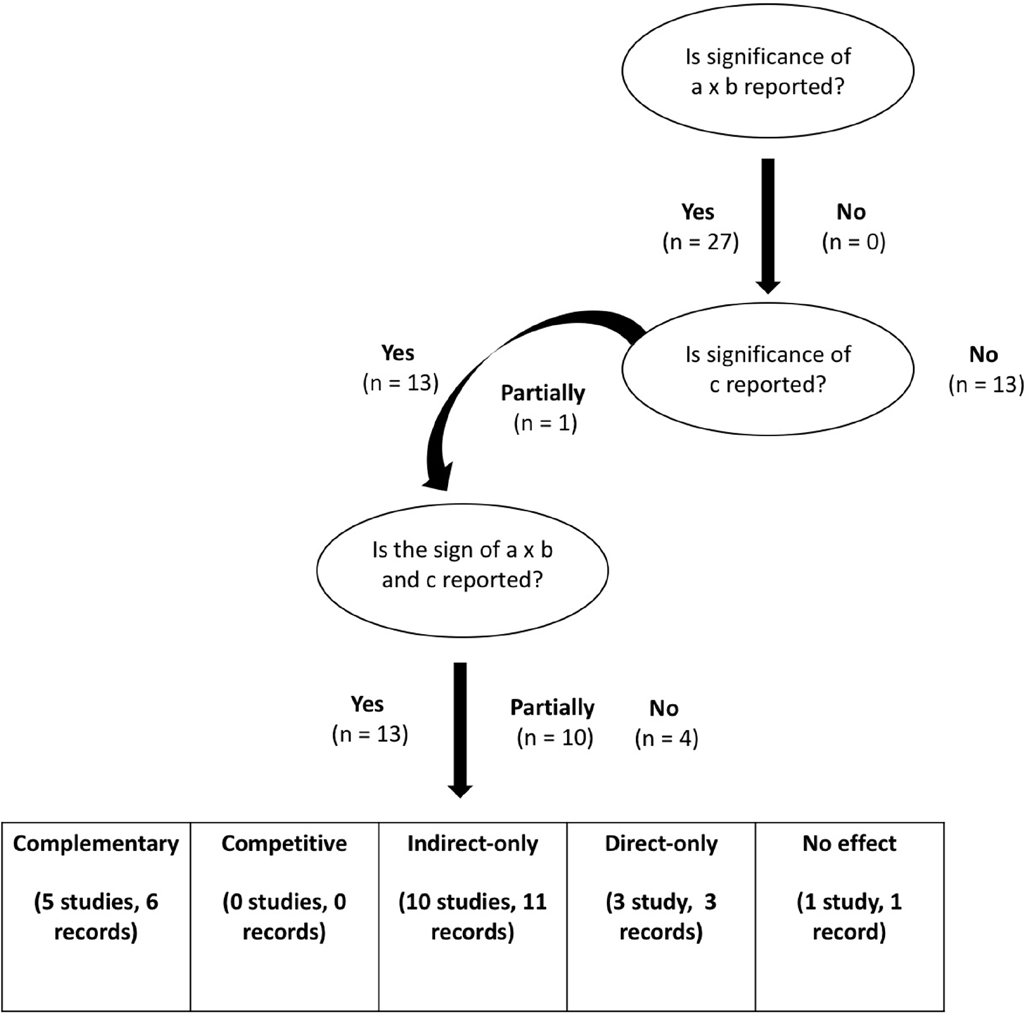
Flow chart of included studies by mediation type and reporting requirements. The flow chart of the included studies is illustrated based on meeting key steps for reporting and categorising mediation: (1) the significance of path a×b, (2) the significance of path c and (3) the sign of paths a×b and c. All included studies reported the significance of the a×b path as this was a key inclusion criteria. From there, the number of included studies (n) that reported the significance of path c was recorded, followed by the number of included studies that reported the sign of the a×b and c paths. At the bottom of the flow chart, we have the total number of studies for each category of mediation. Note that for studies that partially demonstrated the significance of path c and partially demonstrated the signs of a×b and c, this meant that these papers met the criteria for some findings but not for all. Only 13 studies fully met these criteria, and therefore mediation categories were determined. This figure reflects key findings in online supplemental 13 and was adapted from Zhao *et al*.^13^ .

Following the Zhao *et al*’s^13^ framework, all included studies (n=27) reported the significance of the IE. However, out of these studies, only 13 studies fully reported the significance of the direct effect (DE), after controlling for the IE, and reported the sign of the IE and DE to accurately classify the type of mediation.^13^ Out of these 13 studies, 5 reported complementary mediation, none reported competitive mediation, 10 reported indirect-only mediation, 3 reported direct-only effects and 1 reported no effects (non-mediation) (figure 5). In terms of overall quality assessment for mediation, following the Zhao *et al*’s^13^ and Kazdin’s^29^ (2007) frameworks, the overall rating (score range 0–30) was high for reporting the IE (score of 22), high for plausibility or coherence (score of 25.5), intermediate (score of 12.5) for timeline and low (score of 6) for gradient (online supplemental 12).

## Conclusions and clinical implications

The aim of this scoping review was to summarise the literature on potential target mechanisms underlying MBPs and formal mindfulness practices that may drive change in mental health and well-being across the population. The results of this review demonstrated limited high-quality evidence in the context of individual formal mindfulness practices, as one key component of MBPs. The current review identified additional knowledge gaps in terms of the evidence for potential mechanisms by outcome (figure 2), mental health strategy (figure 3) and active comparator (figure 4).

**Figure 6:**
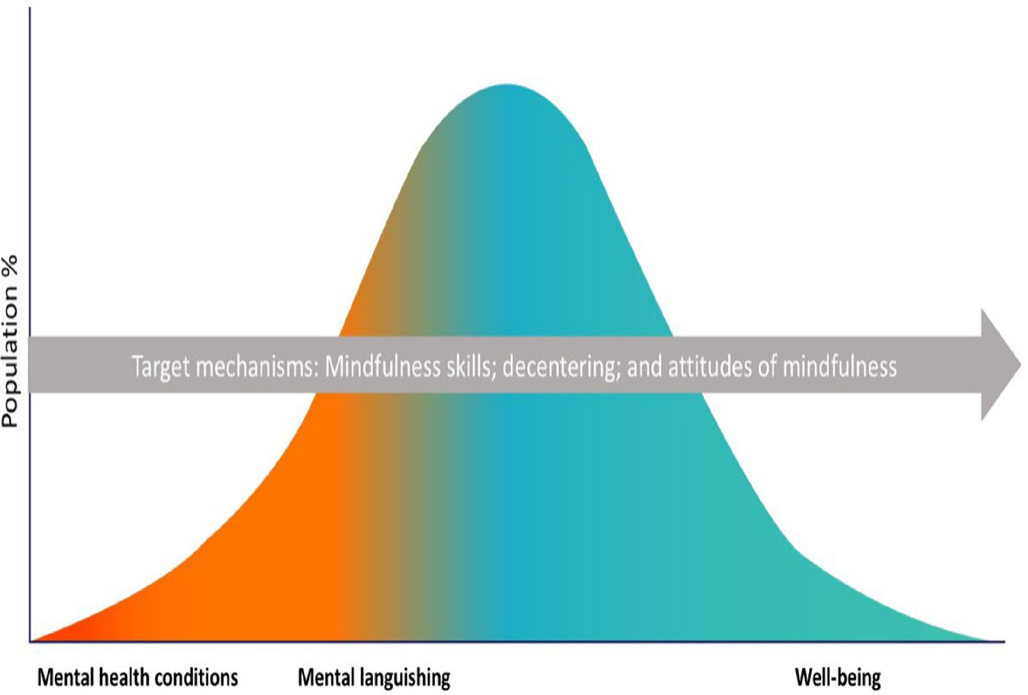
Conceptual framework of target mechanisms. A conceptual framework of target mechanisms that may be responsible for shifting change across the entire population distribution (eg, from mental health conditions to mental languishing to well-being) is illustrated. In this figure, the ‘red’ region on the left-hand side represents outcomes pertaining to mental health conditions (eg, anxious, depressive and adjustment disorders; psychosomatic disorders; behavioural disorders, etc). The ‘orange-to-yellow’ region represents outcomes pertaining to mental languishing (eg, mental health symptoms, stress, burnout, etc). The ‘blue’ region represents outcomes pertaining to well-being (eg, mental well-being, quality of life, positive affect, positive states of mind, etc). The grey arrow represents the proposed mechanisms of change (ie, mindfulness skills, decentering and attitudes of mindfulness). This conceptual framework was informed by the results of the scoping review and summarises key hypotheses regarding the ‘how’ and ‘why’ mindfulness-based programmes and practices work across the whole population distribution. However, future research will need to further interrogate this conceptual framework in different contexts (eg, mental health strategies, ie, promotion), outcomes (eg, well-being) and active comparators (eg, self-help mindfulness, attentional control). Illustration by Delphine Perrot.

Looking at the evidence by outcomes, the results provided preliminary support for mindfulness skills, decentering, and attitudes of mindfulness as potential target mechanisms (figure 2). The largest number of significant IEs was found in the context of mental languishing outcomes (ie, mental health symptoms), which builds on the results of past reviews.^9^,^12^ In the context of outcomes relating to mental health conditions, the evidence was mixed. Significant IEs for mindfulness skills, decentering and attitudes of mindfulness were also reported in relation to well-being outcomes; however, future research will need to replicate these findings. The evidence by mental health strategy demonstrated consistent evidence for mindfulness skills, decentering and attitudes of mindfulness in the context of prevention, mixed evidence in the context of treatment and preliminary evidence in the context of promotion (figure 3). In terms of the evidence by active comparator (figure 4), preliminary evidence for changes in mindfulness skills, decentering and attitudes of mindfulness was found when the mindfulness condition was compared with TAU. However, TAU ranged across studies (eg, continuation of medication and/or psychotherapy and/or regular doctor visits) with differing levels of adherence. For many studies, TAU was also included within the mindfulness condition, which made it difficult to understand mindfulness-specific effects. Overall, very few studies replicated findings whereby the same putative mechanism was tested in the context of one active comparator category. The largest number of opposite direction IEs were found in the context of comparing an MBP to cognitive–behavioural therapy or cognitive interventions (CBT/CI), which may suggest that CBT/CI may demonstrate superiority in terms of activating certain putative mechanisms (eg, empathy, reappraisal self-efficacy, safety behaviours) in relation to specific outcomes (eg, social anxiety). This mirrors what past reviews have found in terms of outcomes, with limited evidence for MBP superiority when compared with current gold-standard treatments (eg, CBT).^5 7^ Across all evidence gap maps (figures2^4^), one key hypothesis generated was that changes in mindfulness skills, decentering and attitudes of mindfulness may be responsible for shifting the entire population more towards improved outcome (figure 6). However, this hypothesis will need to be tested in future work across a wider range of population samples, outcomes and active comparators.

In addition to mapping out the evidence for mechanisms underlying MBPs and mindfulness practices, another aim of this review was to interrogate the quality of the evidence with regard to meeting key testing and reporting requirements for mediation. All eligible studies tested the IE and implemented an RCT design with a comparator that had some active components (ie, more than the mere passage of time) as this was a key inclusion criteria. Some excluded studies explored the IE but only within the mindfulness condition^30^,^32^ and others compared the MBP or mindfulness practice to a passive control group (ie, wait list).^1433^,^36^ Additional studies were excluded on the basis of using alternative analytic methods (eg, simple correlations) that could not effectively calculate the IEs or its significance and, in consequence, determine the type of mediation.^37^,^40^ Out of the included studies, 13 fully met all reporting requirements (figure 5). Out of these thirteen studies, the majority found indirect-only mediation and demonstrated consistent support for mindfulness skills, decentering and attitudes of mindfulness (ie, self-compassion) as putative mechanisms across a range of outcomes (eg, stress, depression, anxiety, burnout, well-being) and samples (eg, secondary school teachers, partial remitters for MDD) (online supplemental 13).

Additional criteria for establishing and reporting a mechanism of change were also evaluated.^29^ The included records scored a high quality score for the reporting of the IE. However, the assumption is that this quality score would be much lower if we looked across all mechanism studies conducted in the mindfulness field, considering the inclusion and exclusion criteria of this review. For the other criteria explored (online supplemental 12), the overall quality score was high for the ‘plausibility and coherence’ criterion, intermediate for the ‘timeline’ criterion and low for the ‘gradient’ criterion. For the timeline criterion, only 12 studies met this criterion which suggests that future work should incorporate research designs or analytic strategies that minimise measurement overlap for proposed mediators and outcomes. This is an area of research that is advancing and there are statistical analyses that can help disentangle this relationship (eg, cross-lagged panel models^41^) even when there is overlap with measurement timepoints. Overall, this criterion helps with increasing confidence in establishing temporality, which indicates that change in the proposed mediator occurs before change in outcome. For the gradient criterion, only five studies met this criterion which indicates that more work is required to establish that different doses (eg, longer home-based practices or number of sessions) are related to changes in the proposed mediator and outcome to help increase confidence that changes in the mediator are a result of mindfulness-specific components. The ‘high’ score for plausibility and coherence suggests that the selected studies were mostly driven by a proposed theoretical framework, which is one strength of the included studies (n=21) which met this criterion (online supplemental 12).

One strength of this scoping review includes the breadth of the topic and search strategy to provide a comprehensive summary of putative psychological mechanisms and to identify knowledge gaps. Another strength of the review is the adherence to emerging mediation frameworks,^13 29^ which meant that the highest-quality evidence for mediation was identified. In terms of limitations, the evidence gap maps captured multiple findings from the same study and many studies presented findings across multiple categories (eg, mechanisms, outcomes, mental health strategy) which could inflate the evidence; however, the number of studies per category are specified to help manage this and the hope is that this presentation will help clearly map out what has and has not been explored. Although many researchers were involved in the conceptual mapping of measurements onto categories, future work is needed to reach a consensus on how mindfulness and overlapping processes (eg, attention and awareness, attitudes of mindfulness, and decentering) are operationally defined. Additionally, the inclusion of RCTs with ‘active comparators’ may have skewed the results to more clinical samples and ‘high-risk’ approaches (eg, treatment and indicated prevention) whereby active comparators are more established compared with alternative strategies (eg, universal prevention, promotion) which address a wider distribution of the population (eg, general population samples). Moreover, the majority of studies included TAU control groups which can be flawed with issues such as resentful demoralisation whereby participants in the TAU arm, aware of the treatment arm, may feel disheartened and report less desirable outcomes. To address these limitations, future work will need to explore active comparators in a range of contexts (eg, mental health strategies, outcomes, population samples) and consider how to reduce potential bias. Qualitative studies and alternative research designs (eg, quasi-experimental) were not included in the current review to isolate the highest quality evidence for mediation; however, these methods may be useful in identifying additional mechanisms of change. Therefore, qualitative methods and alternative designs may serve as starting points and future work can then test hypothesised mechanisms with an RCT design and by following key testing and reporting requirements (see online supplemental 14).

Focusing solely on those with mental health conditions or who are languishing leaves the majority of the population susceptible to entering ill health without any intervention.^42^ Therefore, there is a global health argument for going beyond treatment and looking at mental health across a wider distribution of the population. Adaptations of MBCT/MBSR for the general population have been developed, and there is preliminary evidence that supports their efficacy.^14 16 43^ However, more work is required to extrapolate the putative mechanisms that may be shared across or unique to these adaptations and their parent programmes to help optimise their effectiveness and increase scalability. In this review, the results provided preliminary support for mindfulness skills, decentering and attitudes of mindfulness as key intervention targets for addressing mental health of whole populations (figure 6). However, the field moving forward requires more RCTs, with active comparators that include more than the mere passage of time and TAU, to further understand the unique benefits of MBPs and their potential utility of applying them more broadly. In parallel, proper testing and reporting of mediation^13 29 44^ is needed to help refine the proposed theoretical framework to then help support thoughtful adaptations of MBPs.

## Supplementary material

10.1136/bmjment-2023-300955 online supplemental file 1

## Data Availability

All data relevant to the study are included in the article or uploaded as online supplemental information.
