# Supplementary material for: Target mechanisms of mindfulness-based programmes and practices: a scoping review
Source: BMJ Ment Health. 2024 Aug 24;27(1):e300955. doi: 10.1136/bmjment-2023-300955 (PMC11344521; doi:10.1136/bmjment-2023-300955)
Supplement: online supplemental file 1 [file bmjment-27-1-s001.pdf]

Target Mechanisms of Mindfulness-Based Programmes and Practices: A Scoping Review

Supplementary Materials

Supplement 1. Updated study protocol document...2

Supplement 2. Flow chart of changes made since initial review registration………........................18

Supplement 3. Search string used for each database...19

Supplement 4. References for included papers…………………………........................………...…23

Supplement 5. Content analysis of key MBP curricula for practice terms…………………….........27

Supplement 6. Visual depiction of indirect effect and relevant paths (a, b, c, and c')………............36

Supplement 7. Zhao et al. (2010) and Kazdin (2007) recommendation for establishing a mechanism of action...37

Supplement 8. Further details on inclusion and exclusion criteria..38

Supplement 9. Further details on methodology for evidence gap map development...........................39

Supplement 10. Conceptual mapping of mechanism and outcome terms...42

Supplement 11. Operational definitions of mental health strategies...52

Supplement 12. Quality assessment of included studies based on Zhao et al. (2010) and Kazdin (2007)..54

Supplement 13. Description of included randomized controlled trials (RCTs) with active comparator(s)………..……..56

Supplementary 14. A refined framework for testing and reporting mediation..72

Supplement 1. Updated study protocol document.

The mechanisms through which mindfulness-based programmes and practices produce change across the population: A scoping review protocol

Shannon Maloney[1], Merle Kock[2], Yasmijn Slaghekke[1], Lucy Radley[1,3], Alba Lopez-Montoyo[4], Jesus Montero-Marin [1, 5, 6] *, and Prof Willem Kuyken[1] *

*Joint senior authorship (co-last)

[1]Department of Psychiatry, University of Oxford, Oxford, OX37JX, UK

[2]Centre for the Psychology of Learning and Experimental Psychopathology, KU Leuven, Belgium

[3]Department of Experimental Psychology, University of Oxford, Oxford, OX26GG, UK

[4]Department of Basic, Clinical Psychology, and Psychobiology, Universitat Jaume I, Castellón, Spain

[5]Teaching, Research & Innovation Unit, Parc Sanitari Sant Joan de Déu, Sant Boi de Llobregat, Spain

[6]CIBER of Epidemiology and Public Health (CIBERESP), Madrid, Spain

Author Note:

Shannon Maloney: https://orcid.org/0000-0002-6939-2298

Merle Kock: https://orcid.org/0000-0001-9429-6321

Lucy Radley: https://orcid.org/0000-0002-1976-8912

Jesus-Montero Marin: https://orcid.org/0000-0001-5677-1662

Alba Lopez-Montoyo: https://orcid.org/0000-0001-7494-3499

Willem Kuyken: https://orcid.org/0000-0002-8596-5252

Corresponding author: Shannon Maloney; Department of Psychiatry, University of Oxford, Oxford, OX37JX, UK; Email: shannon.maloney@psych.ox.ac.uk

Abstract

Some of the strongest evidence for these mindfulness-based programmes (MBPs) is in the context of recurrent depression and chronic pain, but there is also scope for investigating several adaptations of these programmes across a wider distribution of the population. Moreover, further consolidation of the evidence in terms of the mechanisms through which these MBPs and their key components (i.e., mindfulness practices) produce change is needed. Five electronic databases (PubMed, PsycINFO, Embase, Scopus, and the Cochrane Central) will be searched for eligible studies. Randomized controlled trials (RCTs), with an active comparator, that have evaluated potential mechanisms of a MBP (or an individual mindfulness practice) will be included. The following information will be piloted and extracted for charting: Name of author(s); year of publication; sample characteristics; intention behind mindfulness-based programme (treatment, prevention, and promotion); description of mindfulness condition and active comparator; dosage; delivery mode; reported mediators and outcomes; data-analytic approach for testing mediation; and key findings. Adherence to methodological requirements in establishing a mechanism will also be reported. A qualitative narrative summary of the results will be described in accordance with the information extracted for charting. The results from this review will help inform mindfulness teachers, researchers, and health-care professionals who intend to adapt MBPs or practices to unique populations and samples. Registration DOI: 10.17605/OSF.IO/XJDSU.

Keywords: Mindfulness-based programme, mechanism, mediator

Background

Mindfulness, defined as bringing moment-to-moment awareness of one's thoughts, emotions, and bodily sensations in a non-evaluative and accepting way, was introduced as a secular practice in mainstream settings in the late 1970s when Jon Kabat-Zinn developed the mindfulness-based stress reduction (MBSR) programme to help relieve chronic pain symptoms (1). Mindfulness-based cognitive therapy (MBCT), an adaptation which includes elements of cognitive therapy, was created in the early 2000s as a strategy to help prevent depressive relapse (2). MBSR/MBCT are mindfulness programmes traditionally formatted as eight-week courses with weekly group sessions guided by a trained mindfulness instructor. Some of the strongest evidence for these mindfulness-based programmes (MBPs) is in the context of recurrent depression (3) and chronic pain (4), but there is also scope for investigating several adaptations of these programmes across a wider distribution of the population (5,6).

A population-based strategy can be adopted to investigate the effects of MBPs across the population from mental ill health to flourishing (e.g., optimal well-being). According to this approach, most conditions fall on a continuum in that there are few 'highs' and 'lows' and many values that fall somewhere in between. According to Geoffrey Rose, by solely focusing on those on the lower end of this distribution (e.g., mental ill health), this leaves a larger proportion of the population susceptible to entering these higher-risk categories without any intervention (7). In the context of MBPs, past research has mostly examined the application of these programmes in the lower end of this distribution as treatment (e.g., current symptoms of anxiety and depression) and prevention (e.g., reducing risk of depressive relapse) strategies (3,8). However, there is also a growing body of work that has examined the application of these programmes in the higher end of this distribution as a promotion strategy (e.g., improving well-being in non-clinical samples) yet higher-quality randomised controlled trials (RCTs) are needed (9).

To uncover the processes through which specific programmes, such as MBPs, have an effect on a particular outcome, Kazdin (2007) argues that one must elucidate the mechanisms of change. These variables explain how a programme (P) influences change in a specific outcome (O) by illuminating the 'interim processes' between the programme and the outcome (P→O). The ultimate goal with uncovering a mechanism of change is therefore to understand this relationship (P→Mechanisms→O) from 'beginning to end' (10). There are requirements put forth that help elaborate a mechanism of change. These requirements involve establishing the mediator (M), which demonstrates the relationship (P→O) statistically and is therefore an intermediate step in establishing a causal relation and mechanism of change. When

testing for mediation, Zhao and colleagues (2010) argue that the significance and size of the indirect effect (*path a x b*) is the most important (11). This framework differs from the commonly used three-step approach. This approach, adopted by Baron and Kenny (12), argues for *full mediation* when (a) variations in the levels of the independent variable (programme) significantly account for change in the dependent variable (outcome) (*path a*); (b) variations in the proposed mediator significantly account for variations in the dependent variable (*path b*); and (c) a non-significant association between the independent variable and dependent variable after controlling for *path a* and *path b (path c)*. If the first two conditions are met, but a significant *path c* is found, then Baron and Kenny (1986) argue that this is *partial mediation*. According Zhao and colleagues (2010), mediation can still exist if *path c* is significant and that this usually means that one or more mediators were not included in the model. Ultimately, if this requirement of establishing mediation statistically is met, along with other requirements [see Kazdin (2007)], then this increases the likelihood of a mechanism of change. It is important to note that the distinguishing factors between a mediator and mechanism are discrete in that both attempt to uncover the same processes yet a mediator is established at an earlier stage of research whereas a mechanism is uncovered after extensive replication studies. Mediators can be understood as unrefined mechanisms that require further investigation to determine whether key requirements are met and the finding has been supported in numerous studies.

The existing literature on the mechanisms through which MBPs work across the entire population is sparse and studies typically do not meet all requirements for establishing a mechanism of change. Gu et al. (2015) identified twenty papers, in a systematic review and meta-analysis, that examined mediators of MBCT and MBSR on mental health and well-being outcomes in clinical samples (e.g. adults with symptoms of depression) and non-clinical samples (e.g. students with limited or no previous mindfulness training). The main results indicated strong evidence for cognitive and emotional reactivity, moderate evidence for mindfulness and repetitive negative thinking, and weak evidence for self-compassion and psychological flexibility as potential mechanisms through which MBPs produce change (13). However, the eligible studies include quasi-experimental designs and include a range of mediation tests that do not necessarily follow the recommendations outlined by Zhao et al. (2010). One aim of this review is to build on the findings of this review to provide stricter inclusion criteria to isolate the highest-quality evidence for mediation. Moreover, the current review aims to further consolidate the evidence of the mechanisms of MBPs in relation to its intended purpose (e.g., treatment, prevention, or promotion) to help further understanding of mechanism specificity and which mechanism drive change across the population. Other systematic reviews (14–16) have investigated mechanisms of MBPs in clinical

samples (e.g., individuals with recurrent depression or psychological and physical conditions) or in relation to outcomes of mental ill health. The current review aims to also build on these reviews by providing more open inclusion criteria based on the population sample and outcomes to help summarize the evidence of mechanisms across the entire adult population.

In addition to exploring these variables in MBPs, there is also an opportunity to explore the processes through which key components of MBPs (i.e., individual mindfulness practices) produce change. Kazdin (2007) argues that a deeper understanding of how individual components work outside the framework of the entire programme can help one further understand how the programme works on the whole. Mindfulness practices are regarded as a core component of MBPs (17) and conceptual frameworks (1,2,18) along with early research (19,20) suggest that these individual meditation practices can produce change in outcomes without being nested within the entire MBP. Past research seems to highlight practices that are derived from the general framework of mindfulness-based programmes, but the Crane et al. (2017) definition is not implemented which makes it difficult to pin-point whether or not these practices originate from MBSR/MBCT and their adaptations. Sauer-Zavala et al. (2012) compared three different meditation practices (sitting meditation, mindful yoga, and body scan) and found greater improvements in psychological well-being in mindful yoga compared to the other practices, and more change in emotion regulation difficulties in the mindful yoga condition compared to the body scan condition. Graduate students who led the practices followed scripts from MBSR/MBCT curricula. This helps clarify the effects of these practices within these frameworks, but it is important to note that practice adherence was not measured. Without this measurement, it is difficult to fully understand how change is produced (21).

Research on the mechanisms through which individual mindfulness practices produce change is in its early stages. Kok and Singer (2017) suggest that each meditation practice has a 'phenomenological fingerprint' or rather a unique subjective experience. For instance, it was observed that the body scan meditation brought about greater changes in interoceptive awareness (the ability to use the body as a barometer for one's thoughts and emotions) compared to a breathing, loving-kindness, and observing-thought meditation (22). Britton and colleagues (2018) tested two 'active ingredients' of MBCT by dismantling this programme and creating two structurally equivalent curricula that either implemented focused attention (FA) elements or open-monitoring (OM) elements and compared these curricula to a formal MBCT intervention in individuals with mild to severe affective disturbances. One interesting result indicated that individuals that took part in the OM programme demonstrated greater improvements in non-reactivity whereas

participants in the FA programme experienced more change in attentional control. However, given that there was no ‘business as usual’ control, it is difficult to understand whether changes in outcome were as a result of practice-specific effects or non-specific effects (23). Overall, more work is required in this area and further consolidation of the current evidence on potential mechanisms of individual mindfulness practices in relation to the proposed strategy (treatment, prevention, promotion), population sample, context, active comparator, and outcome is needed.

This scoping review will provide a unique contribution to the field by summarizing the existing literature on (i) the mechanisms through which one key component of MBPs (individual mindfulness practices) produce change and on the (ii) process variables of MBPs and individual mindfulness practices in relation to mental health and well-being outcomes and unique population samples. To the knowledge of the authors, there is no review that has comprehensively examined these process variables across the entire population, which includes clinical samples (the ‘highs’), non-clinical samples (‘lows’) and the distribution in between. In light of the limited evidence on the change processes of both MBPs and the practices derived from these programmes, across the entire population, the rationale for this review is to provide a summary of the current evidence to inform future research studies and stakeholders in the field who would be interested in adapting these programmes and practices.

Objectives

The primary aim of the current scoping review is to examine the mechanisms through which MBPs and practices produce change across the population. Due to the novelty and breadth of this research topic, the authors decided to follow the stages and methodology of a scoping review.

The objective of this scoping review is to explore the following questions: (a) through which mediators and mechanisms do MBPs and practices produce change in the adult population? (c) Which data analytic approaches for testing mediation (e.g. path analysis, structural equation modelling) are used? (d) To what extent have the identified papers met formal recommendations laid out by Kazdin (2007) in establishing pathways of change? The key elements of these research questions include the entire population [the ‘highs,’ ‘lows,’ and the distribution in between these two ends] (population), mediators and mechanisms (concept), and MBPs and practices (context). Several established and accepted frameworks (24–26) will be used to drive the methodology and stages of the scoping review. This review protocol adheres to the PRISMA-P checklist along with the PRISMA extension for Scoping Reviews (PRISMA-ScR) checklist (27,28).

Method

Eligibility criteria

Participants

Studies will be included if they evaluate a MBP or individual mindfulness practice irrespective of the population sample (e.g., clinical, sub-clinical, non-clinical). Therefore, the population sample can fall anywhere on the population distribution and can be represented by the 'highs,' 'lows,' and the full range of values in between (Figure 1). For clarity, clinical samples will be defined by the presence of formal clinical diagnoses to help distinguish from non-clinical samples. Participants must be adults (aged 18 or above).

Context

Eligible studies must include MBPs, defined by Crane and colleagues (2017) as predominately MBSR and MBCT and any adaptation of these programmes, or mindfulness practices that derive from these programmes. The rationale for focusing on these programmes and practices is because this is where the evidence-base is strongest. Mindfulness-informed programmes (e.g. Acceptance and Commitment Therapy, Dialectical Behavior Therapy, and Mindful Self Compassion) will be excluded because sustained mindfulness training is not a core component of these programmes. Second-generation mindfulness programmes will also be excluded because the evidence-base is still underdeveloped and the range of programmes is more heterogeneous (17).

As discussed, eligible studies will include mindfulness practices derived from MBPs. Due to heterogeneity of terms used for mindfulness practices, a content analysis was conducted to determine the extent that different terms in the literature related to the same or different practices. Two reviewers developed this analysis and were in agreement. According to four key curricula for MBCT and MBSR (1,2,29,30), 'formal' mindfulness practices are key ingredients of the MBP approach as they allow for deeper engagement whereas 'informal' practices are used to cultivate mindfulness specifically during daily activities. For the scope of this review, eligible studies will examine formal practices because the evidence-base is stronger and they have clearer operational definitions and parameters compared to informal practices. Future studies will need to investigate informal practices in addition to other practice-related components of MBPs (e.g. silent retreats, orientation sessions, home-based versus in-class practices).

The existing literature on a definition or set criteria for formal practices is sparse and inconclusive. Crane and colleagues (2014) operationalize formal practices as approximately 40 minutes in duration and as a practice that is engaged with at least three times each week on average (which was also used by Perich et al. 2013) (31,32). Moreover, Hawley et al. (2013) argue that guidance is provided for formal practices and that these practices are engaged with for a specific amount of time whereas there is less structure and no required length or time for informal practices (33). One thing to keep in mind is that these studies formulated these operational definitions in the context of recurrent depression. Therefore, there might be scope for a more flexible operational definition (e.g., in terms of dosage).

The content analysis, adopted to clarify the eligibility criteria for formal practices, rests on the assumption that practices used across all four key MBP curricula (1,2,29,30) are core to the MBP approach and regarded as formal meditation practices. This process began by hand searching these texts to compile a list of all the practices implemented across these MBCT and MBSR curricula. In addition to having two reviewers, experienced mindfulness instructors were also consulted to help match these practices across each curriculum based on the intentionality or theme behind the practice. From there, a list of key characteristics, shared between all these practices, was gathered. The determined key elements (listed below) were then used to clarify the eligibility criteria for studies that evaluate mindfulness practices. It is important to note that this content analysis was used to specify eligibility criteria for formal mindfulness practices and should be replicated in future studies to validate its usage.

In summary, a formal mindfulness practice must be:

- Home-based, in that the practice is predominately engaged with at home in the context of the original MBP
- Scheduled with CD or audio guidance in the context of the original MBP (supported by Hawley et al., 2013)
- Practiced at least three times per week, during the appropriate weekly session, in the context of the original MBP (supported by Crane et al., 2014 and Perich et al., 2013)

For the scope of this review, outcomes related to mental health and well-being will be prioritized to help consolidate the evidence of the mechanism of MBPs and individual practices in relation to the proposed intention (mental health treatment, prevention, or promotion strategy), following definitions used by (34,35). Moreover, in an effort to synthesize the highest-quality evidence and to help articulate specificity of mechanisms, only randomised-controlled trials with active comparators will be eligible. English and Spanish publications will be included, in light of the primary language

of the research team, and a specific year range (1979 and onwards) will be considered. The rationale for starting with the publication year of 1979 is because this year coincides with the introduction of mindfulness into mainstream settings when MBSR was developed.

Concept

Studies will be included if they evaluate mediators of a MBP or practice following the recommendations outlined by Zhao and colleagues (2010) which prioritizes the significance and size of the indirect effect. The extent to which these eligible studies adhere to requirements laid out by Kazdin (2007) (13) in establishing pathways of change will also be explored.

Search strategy

Five electronic databases (PubMed, PsycINFO, Embase, Scopus, and Cochrane Central) will be systematically searched for eligible studies. These databases were chosen based on their relevance to the social and medical sciences. Other sources (e.g. Web of Science conference proceedings, PsycEXTRA, or connectedpapers.com) will also be systematically searched to identify grey literature.

The primary research question ('Through which mediators and mechanisms do MBPs and practices produce change in the adult population?') was broken down by population, context, and concept terms and then a list of synonyms and related terms were developed. For the search, the population terms were left open to ensure that more specific samples (e.g. nonclinical and clinical) were not excluded. Key search terms from the primary research question include 'mediators,' 'mechanisms,' and 'mindfulness.' A list of synonyms and related terms were developed by first running mock search in one electronic database (PubMed) and searching keywords in relevant papers. Experts in the field, including a research librarian, were also consulted to help refine the search terms. Moreover, the content analysis used to clarify the criteria for mindfulness-based practices (see above) was also used to specify the search terms. Specific practice terms were included as direct phrases to reduce noise and to narrow focus on practice terms specifically used in the four key MBP curricula used for guidance.

The snowballing method or PEARL citation method will be used for eligible studies to help identify other included studies. Reference lists of relevant systematic reviews and meta-analyses will also be searched by hand to locate other

possible eligible studies. Corresponding authors of eligible studies will be contacted to retrieve further information, if needed. A PRISMA flow diagram will be used to track sources identified through databases and other sources, number of duplicates removed, number of papers screened for eligibility, and ultimately the number of papers included for narrative synthesis. Any amendments made will be documented and provided with a rationale within the final publication.

In the initial scoping review registration form (July 16, 2020; DOI: https://doi.org/10.17605/OSF.IO/XJDSU), it was outlined that the current review would additionally include studies that have examined moderators of MBPs and individual mindfulness practices and the outcomes were left open. However, after running the initial search and re-evaluating the scope of the review, the inclusion of moderators was removed. Moreover, the outcome variables were narrowed down to mental health and well-being to increase conceptual clarity. Moreover, in the initial registration, it was suggested that the Mindfulness journal would be searched separately. However, since the included databases also search this journal the authors of this review felt that it was unnecessary to search this journal independently. From the initial registration, it was also specified that Cochrane Central, as one key database of the Cochrane Library, will be searched. Lastly, connectedpapers.com was additionally suggested as a potential source to search for grey literature and Covidence software was additionally suggested for data management alongside the use of EndNote. The initial search was run in December 13th 2020.

Data extraction

EndNote and Covidence software will be used to manage studies throughout the review process. Two reviewers will be responsible for screening titles and abstracts for eligibility. Any uncertainties will be discussed with a third reviewer. Two reviewers will also read the full-texts independently to determine final inclusion. In regards to data charting, the aim is to extract the following information from each included study: Name of author(s); year of publication; sample characteristics; intention behind mindfulness-based programme (treatment, prevention, and promotion); description of mindfulness condition and active comparator; dosage; delivery mode; reported mediators and outcomes, data-analytic approach for testing mediation, and key findings. One reviewer will be in charge of charting the data from the included sources. A second reviewer will confirm a percentage of the data collected from these sources and where there is any dispute a third reviewer will help make the final decision.

Strategy for data synthesis

A qualitative narrative summary of the results will be described according to the information extracted for charting. Depending on the amount of data, the idea is to develop a conceptual framework on the mechanisms through which MBPs and practices produce change across the population. If appropriate, a schematic diagram or visual depiction will be provided to summarize the findings of the review.

Dissemination

The results of this scoping review will be submitted for publication in a peer-reviewed journal.

Discussion

The overarching aim of this scoping review is to examine the mechanisms through which MBPs and mindfulness practices work across the entire population. To the knowledge of the authors, this scoping review will be the first to extensively examine the change processes of MBPs and practices across the entire population distribution. The hope is that the results from this review will help inform mindfulness instructors, researchers, and health-care professionals who intend to adapt MBPs or practices.

References

1. Kabat-Zinn J. Full catastrophe living: using the wisdom of your body and mind to face stress, pain, and illness. New York: Bantam Books; 2013.

2. Segal Z, Williams M, Teasdale J. Mindfulness-Based Cognitive Therapy for Depression, Second Edition. Guilford Publications; 2018. 473 p.

3. Kuyken W, Warren FC, Taylor RS, Whalley B, Crane C, Bondolfi G, et al. Efficacy of Mindfulness-Based Cognitive Therapy in Prevention of Depressive Relapse: An Individual Patient Data Meta-analysis From Randomized Trials. JAMA Psychiatry. 2016 Jun 1;73(6):565.

4. Grossman P, Niemann L, Schmidt S, Walach H. Mindfulness-based stress reduction and health benefits: A meta-analysis. J Psychosom Res. 2004 Jul 1;57(1):35–43.

5. Strauss C, Gu J, Montero-Marin J, Whittington A, Chapman C, Kuyken W. Reducing stress and promoting well-being in healthcare workers using mindfulness-based cognitive therapy for life. Int J Clin Health Psychol [Internet]. 2021 May 1 [cited 2021 Nov 12];21(2). Available from: https://www.sciencedirect.com/science/article/pii/S1697260021000089

6. Montero-Marin J, Taylor L, Crane C, Greenberg MT, Ford TJ, Williams JMG, et al. Teachers "finding peace in a frantic world": An experimental study of self-taught and instructor-led mindfulness program formats on acceptability, effectiveness, and mechanisms. J Educ Psychol. 2021;113(8):1689.

7. Rose GA. Rose's strategy of preventive medicine: the complete original text. Oxford University Press; 2008.

8. Goldberg SB, Tucker RP, Greene PA, Davidson RJ, Wampold BE, Kearney DJ, et al. Mindfulness-based interventions for psychiatric disorders: A systematic review and meta-analysis. Clin Psychol Rev. 2018 Feb 1;59:52–60.

9. Galante J, Friedrich C, Dawson AF, Modrego-Alarcón M, Gebbing P, Delgado-Suárez I, et al. Mindfulness-based programmes for mental health promotion in adults in nonclinical settings: A systematic review and meta-analysis of randomised controlled trials. Patel V, editor. PLOS Med. 2021 Jan 11;18(1):e1003481.

10. Kazdin AE. Mediators and Mechanisms of Change in Psychotherapy Research. Annu Rev Clin Psychol. 2007 Apr;3(1):1–27.

11. Zhao X, Lynch JG Jr, Chen Q. Reconsidering Baron and Kenny: Myths and Truths about Mediation Analysis. J Consum Res. 2010 Aug 1;37(2):197–206.

12. Baron RM, Kenny DA. The moderator–mediator variable distinction in social psychological research: Conceptual, strategic, and statistical considerations. J Pers Soc Psychol. 1986;51(6):1173.

13. Gu J, Strauss C, Bond R, Cavanagh K. How do mindfulness-based cognitive therapy and mindfulness-based stress reduction improve mental health and wellbeing? A systematic review and meta-analysis of mediation studies. Clin Psychol Rev. 2015 Apr;37:1–12.

14. Alsubaie M, Abbott R, Dunn B, Dickens C, Keil TF, Henley W, et al. Mechanisms of action in mindfulness-based cognitive therapy (MBCT) and mindfulness-based stress reduction (MBSR) in people with physical and/or psychological conditions: A systematic review. Clin Psychol Rev. 2017 Jul;55:74–91.

15. Maddock A, Blair C. How do mindfulness-based programmes improve anxiety, depression and psychological distress? A systematic review. Curr Psychol [Internet]. 2021 Sep 22 [cited 2022 Oct 1]; Available from: https://doi.org/10.1007/s12144-021-02082-y

16. van der Velden AM, Kuyken W, Wattar U, Crane C, Pallesen KJ, Dahlgaard J, et al. A systematic review of mechanisms of change in mindfulness-based cognitive therapy in the treatment of recurrent major depressive disorder. Clin Psychol Rev. 2015 Apr;37:26–39.

17. Crane RS, Brewer J, Feldman C, Kabat-Zinn J, Santorelli S, Williams JMG, et al. What defines mindfulness-based programs? The warp and the weft. Psychol Med. 2017;47(6):990–9.

18. Woods SL, Rockman P, Collins E. Mindfulness-based cognitive therapy: Embodied presence and inquiry and practice. New Harbinger Publications; 2019.

19. Blanck P, Perleth S, Heidenreich T, Kröger P, Ditzen B, Bents H, et al. Effects of mindfulness exercises as stand-alone intervention on symptoms of anxiety and depression: Systematic review and meta-analysis. Behav Res Ther. 2018 Mar;102:25–35.

20. Schumer MC, Lindsay EK, Creswell JD. Brief Mindfulness Training for Negative Affectivity: A Systematic Review and Meta-Analysis. J Consult Clin Psychol. 2018;86(7):569–83.

21. Sauer-Zavala SE, Walsh EC, Eisenlohr-Moul TA, Lykins EL. Comparing mindfulness-based intervention strategies: differential effects of sitting meditation, body scan, and mindful yoga. Mindfulness. 2013;4(4):383–8.

22. Singer T, Kok B. Phenomenological Fingerprints of Four Meditations: Differential State Changes in Affect, Mind-Wandering, Meta-Cognition, and Interoception Before and After Daily Practice Across 9 Months of Training. Mindfulness. 2017;

23. Britton WB, Davis JH, Loucks EB, Peterson B, Cullen BH, Reuter L, et al. Dismantling Mindfulness-Based Cognitive Therapy: Creation and validation of 8-week focused attention and open monitoring interventions within a 3-armed randomized controlled trial. Behav Res Ther. 2018 Feb 1;101:92–107.
24. The Joanna Briggs Institute Reviewers' Manual 2015: Methodology for JBI Scoping Reviews [Internet]. Australia: The Joanna Briggs Institute; 2015. Available from: https://nursing.lsuhsc.edu/jbi/docs/reviewersmanuals/scoping-.pdf
25. Peters MDJ, Godfrey CM, Khalil H, McInerney P, Parker D, Soares CB. Guidance for conducting systematic scoping reviews. JBI Evid Implement. 2015 Sep;13(3):141–6.
26. Arksey H, O'Malley L. Scoping studies: towards a methodological framework. Int J Soc Res Methodol. 2005;8(1):19–32.
27. Shamseer L, Moher D, Clarke M, Ghersi D, Liberati A, Petticrew M, et al. Preferred reporting items for systematic review and meta-analysis protocols (PRISMA-P) 2015: elaboration and explanation. Bmj. 2015;349.
28. Tricco AC, Lillie E, Zarin W, O'Brien KK, Colquhoun H, Levac D, et al. PRISMA extension for scoping reviews (PRISMA-ScR): checklist and explanation. Ann Intern Med. 2018;169(7):467–73.
29. Williams M, Penman D. Mindfulness: A practical guide to finding peace in a frantic world. Piatkus; 2011.
30. Santorelli SF, Kabat-Zinn J, Blacker M, Meleo-Meyer F, Koerbel L. Mindfulness-based stress reduction (MBSR) authorized curriculum guide. Cent Mindfulness Med Health Care Soc CFM Univ Mass Med Sch. 2017;
31. Perich T, Manicavasagar V, Mitchell PB, Ball JR. The association between meditation practice and treatment outcome in Mindfulness-based Cognitive Therapy for bipolar disorder. Behav Res Ther. 2013 Jul;51(7):338–43.
32. Crane C, Crane RS, Eames C, Fennell MJV, Silverton S, Williams JMG, et al. The effects of amount of home meditation practice in Mindfulness Based Cognitive Therapy on hazard of relapse to depression in the Staying Well after Depression Trial. Behav Res Ther. 2014 Dec 1;63:17–24.
33. Hawley L, Schwartz D, Bieling P, Irving J, Corcoran K, Farb N, et al. Mindfulness Practice, Rumination and Clinical Outcome in Mindfulness-Based Treatment. Cogn Ther Res. 2014 Feb 1;38:1–9.
34. Greenberg MT, Abenavoli R. Universal Interventions: Fully Exploring Their Impacts and Potential to Produce Population-Level Impacts. J Res Educ Eff. 2017 Jan 2;10(1):40–67.

35. Muñoz RF, Mrazek PJ, Haggerty RJ. Institute of Medicine report on prevention of mental disorders: Summary and commentary. Am Psychol. 1996;51:1116–22.

Figure 1. A conceptual example of a population distribution of mental well-being before and after taking part in a MBP.

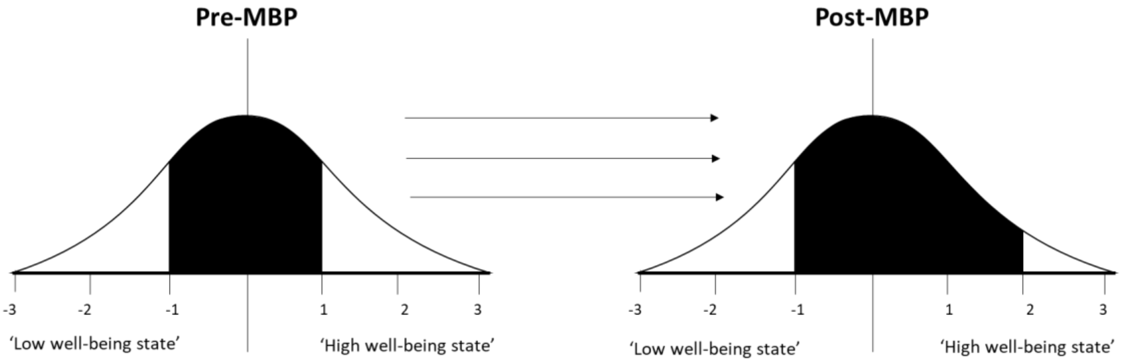

Figure 1 illustrates a conceptual example of a population distribution of mental well-being pre-post a MBP. This figure is inspired by Geoffrey Rose's population-based strategy and the findings of Melzer et al. (2002). The authors of this scoping review consider the population as the full distribution curve rather than the largest proportion of the population (e.g. the shaded region). The population may include a smaller proportion of 'lows' and 'highs' in terms of individuals that have a low mental well-being and high mental well-being. The largest proportion of the population may include individuals with state values that range between these highs and lows. It should be noted that this figure is not based on actual data and is only used to help describe a more nuanced framework of this approach, which recognises that mental well-being may fall on a continuum and that, through increased mindfulness training, individuals may experience increased well-being.

Figure 2. Conceptual model of simple mediation

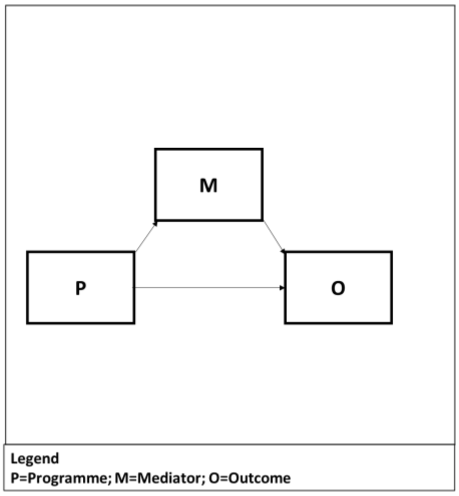

Figure 2 illustrates a conceptual model of simple mediation whereby the programme (P) effects change in outcome (O) through the proposed mediator (M).

Supplement 2. Flow chart of changes made since initial review registration.

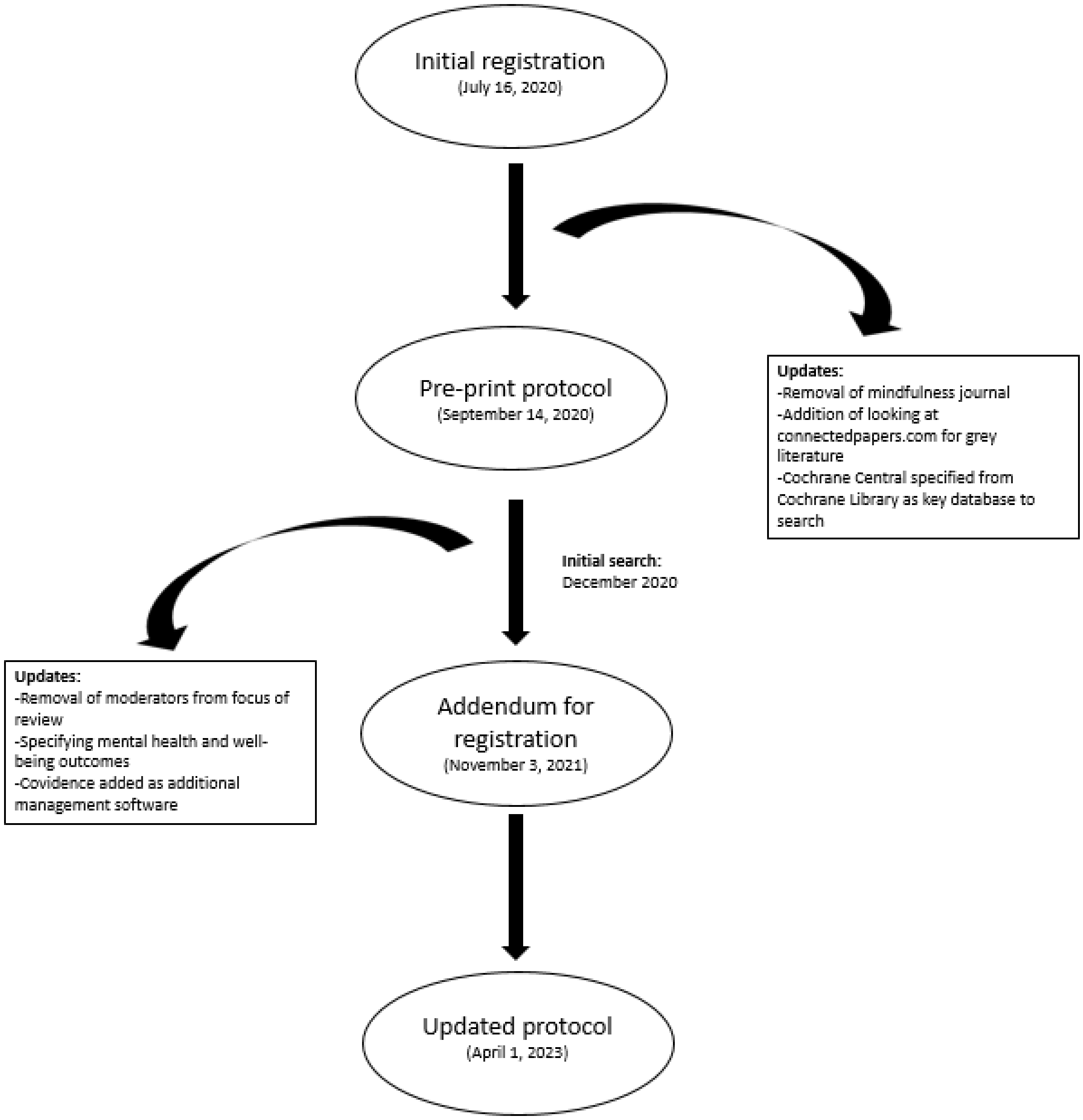

This figure includes a flow chart of the major changes made from the initial review registration through Open Science Framework (OSF) in July 2020 to uploading the updated protocol in April 2023. Please note that the decision to remove *Mindfulness*, as a journal to search, was based on the rationale that one of our other included databases included this journal.

Supplement 3. Search string used for each database.

(1) PubMed

Search: (((((((((mechanism*) OR (mediat*)) OR (moderat*)) OR (practice-specific)) OR (training-specific)) OR (dismant*)) OR ("active ingredient")) OR ("active ingredients")) OR ("indirect effect")) OR ("indirect effects")) AND ((mindful*) OR (Mindfulness[MeSH Terms])) OR (mindfulness-based)) OR (MBI)) OR (MBP)) OR (MBSR)) OR (MBCT)) OR (meditation*)) OR ("sitting meditation")) OR ("formal practice")) OR ("home practice")) OR ("home-based practice")) OR ("formal practices")) OR ("home practices")) OR ("home-based practices")) OR ("body scan")) OR ("diaphragmatic breathing")) OR ("abdominal breathing")) OR ("belly breathing")) OR ("mindfulness of the breath")) OR ("mindfulness of breathing")) OR ("mindfulness of the breath and body")) OR ("mindfulness of the body and breath")) OR ("awareness of breathing")) OR ("breath and body")) OR ("breath meditation")) OR ("breathing-space")) OR ("stretch and breath")) OR ("mindful movement")) OR ("mindful walking")) OR ("walking meditation")) OR ("mindful stretching")) OR ("yoga")) OR ("standing yoga")) OR ("mindful yoga")) OR ("hatha yoga")) OR ("mindful lying down yoga")) OR ("lying down yoga")) OR ("sounds and thoughts")) OR ("mindfulness of sounds and thoughts")) OR ("working with difficulty meditation")) OR ("exploring difficulty meditation")) OR ("befriending")) OR ("mindful breathing")) OR ("breath awareness")) OR ("observing thought"))

(2) PsycINFO & EMBASE

Search:

1	mechanism*.mp. [mp=ti, ab, hw, tn, ot, dm, mf, dv, kw, fx, dq, tc, id, tm, mh]	3383081	Advanced	Display Results More
2	mediat*.mp. [mp=ti, ab, hw, tn, ot, dm, mf, dv, kw, fx, dq, tc, id, tm, mh]	1936804	Advanced	Display Results More
3	mediat*.mp. [mp=ti, ab, hw, tn, ot, dm, mf, dv, kw, fx, dq, tc, id, tm, mh]	1936804	Advanced	Display Results More
4	moderat*.mp. [mp=ti, ab, hw, tn, ot, dm, mf, dv, kw, fx, dq, tc, id, tm, mh]	1029391	Advanced	Display Results More
5	practice-specific.mp. [mp=ti, ab, hw, tn, ot, dm, mf, dv, kw, fx, dq, tc, id, tm, mh]	632	Advanced	Display Results More
6	training-specific.mp. [mp=ti, ab, hw, tn, ot, dm, mf, dv, kw, fx, dq, tc, id, tm, mh]	612	Advanced	Display Results More
7	dismant*.mp. [mp=ti, ab, hw, tn, ot, dm, mf, dv, kw, fx, dq, tc, id, tm, mh]	4852	Advanced	Display Results More
8	"active ingredient".mp. [mp=ti, ab, hw, tn, ot, dm, mf, dv, kw, fx, dq, tc, id, tm, mh]	10439	Advanced	Display Results More
9	"active ingredients".mp. [mp=ti, ab, hw, tn, ot, dm, mf, dv, kw, fx, dq, tc, id, tm, mh]	11143	Advanced	Display Results More
10	"indirect effect".mp. [mp=ti, ab, hw, tn, ot, dm, mf, dv, kw, fx, dq, tc, id, tm, mh]	15222	Advanced	Display Results More
11	"indirect effects".mp. [mp=ti, ab, hw, tn, ot, dm, mf, dv, kw, fx, dq, tc, id, tm, mh]	19645	Advanced	Display Results More
12	mindful*.mp. [mp=ti, ab, hw, tn, ot, dm, mf, dv, kw, fx, dq, tc, id, tm, mh]	34628	Advanced	Display Results More
13	Mindfulness.mp. [mp=ti, ab, hw, tn, ot, dm, mf, dv, kw, fx, dq, tc, id, tm, mh]	28330	Advanced	Display Results More
14	mindfulness-based.mp. [mp=ti, ab, hw, tn, ot, dm, mf, dv, kw, fx, dq, tc, id, tm, mh]	9345	Advanced	Display Results More
15	MBI.mp. [mp=ti, ab, hw, tn, ot, dm, mf, dv, kw, fx, dq, tc, id, tm, mh]	4817	Advanced	Display Results More
16	MBP.mp. [mp=ti, ab, hw, tn, ot, dm, mf, dv, kw, fx, dq, tc, id, tm, mh]	17378	Advanced	Display Results More
17	MBSR.mp. [mp=ti, ab, hw, tn, ot, dm, mf, dv, kw, fx, dq, tc, id, tm, mh]	1942	Advanced	Display Results More
18	MBCT.mp. [mp=ti, ab, hw, tn, ot, dm, mf, dv, kw, fx, dq, tc, id, tm, mh]	1386	Advanced	Display Results More
19	meditation*.mp. [mp=ti, ab, hw, tn, ot, dm, mf, dv, kw, fx, dq, tc, id, tm, mh]	20263	Advanced	Display Results More
20	"sitting meditation".mp. [mp=ti, ab, hw, tn, ot, dm, mf, dv, kw, fx, dq, tc, id, tm, mh]	92	Advanced	Display Results More
21	"formal practice".mp. [mp=ti, ab, hw, tn, ot, dm, mf, dv, kw, fx, dq, tc, id, tm, mh]	121	Advanced	Display Results More
22	"home practice".mp. [mp=ti, ab, hw, tn, ot, dm, mf, dv, kw, fx, dq, tc, id, tm, mh]	1208	Advanced	Display Results More
23	"home-based practice".mp. [mp=ti, ab, hw, tn, ot, dm, mf, dv, kw, fx, dq, tc, id, tm, mh]	74	Advanced	Display Results More
24	"formal practices".mp. [mp=ti, ab, hw, tn, ot, dm, mf, dv, kw, fx, dq, tc, id, tm, mh]	50	Advanced	Display Results More
25	"home practices".mp. [mp=ti, ab, hw, tn, ot, dm, mf, dv, kw, fx, dq, tc, id, tm, mh]	175	Advanced	Display Results More
26	"home-based practices".mp. [mp=ti, ab, hw, tn, ot, dm, mf, dv, kw, fx, dq, tc, id, tm, mh]	18	Advanced	Display Results More
27	"body scan".mp. [mp=ti, ab, hw, tn, ot, dm, mf, dv, kw, fx, dq, tc, id, tm, mh]	2762	Advanced	Display Results More
28	"diaphragmatic breathing".mp. [mp=ti, ab, hw, tn, ot, dm, mf, dv, kw, fx, dq, tc, id, tm, mh]	667	Advanced	Display Results More
29	"abdominal breathing".mp. [mp=ti, ab, hw, tn, ot, dm, mf, dv, kw, fx, dq, tc, id, tm, mh]	351	Advanced	Display Results More
30	"belly breathing".mp. [mp=ti, ab, hw, tn, ot, dm, mf, dv, kw, fx, dq, tc, id, tm, mh]	11	Advanced	Display Results More
31	"mindfulness of the breath".mp. [mp=ti, ab, hw, tn, ot, dm, mf, dv, kw, fx, dq, tc, id, tm, mh]	31	Advanced	Display Results More
32	"mindfulness of breathing".mp. [mp=ti, ab, hw, tn, ot, dm, mf, dv, kw, fx, dq, tc, id, tm, mh]	75	Advanced	Display Results More
33	"mindfulness of breath and body".mp. [mp=ti, ab, hw, tn, ot, dm, mf, dv, kw, fx, dq, tc, id, tm, mh]	1	Advanced	Display Results More
34	"mindfulness of body and breath".mp. [mp=ti, ab, hw, tn, ot, dm, mf, dv, kw, fx, dq, tc, id, tm, mh]	1	Advanced	Display Results More
35	"awareness of breathing".mp. [mp=ti, ab, hw, tn, ot, dm, mf, dv, kw, fx, dq, tc, id, tm, mh]	87	Advanced	Display Results More
36	"breath and body".mp. [mp=ti, ab, hw, tn, ot, dm, mf, dv, kw, fx, dq, tc, id, tm, mh]	83	Advanced	Display Results More
37	"breath meditation".mp. [mp=ti, ab, hw, tn, ot, dm, mf, dv, kw, fx, dq, tc, id, tm, mh]	28	Advanced	Display Results More
38	"breathing-space".mp. [mp=ti, ab, hw, tn, ot, dm, mf, dv, kw, fx, dq, tc, id, tm, mh]	113	Advanced	Display Results More
39	"stretch and breath".mp. [mp=ti, ab, hw, tn, ot, dm, mf, dv, kw, fx, dq, tc, id, tm, mh]	0	Advanced	Save More
40	"mindful movement".mp. [mp=ti, ab, hw, tn, ot, dm, mf, dv, kw, fx, dq, tc, id, tm, mh]	105	Advanced	Display Results More

41	"mindful walking".mp. [mp=ti, ab, hw, tn, ot, dm, mf, dv, kw, fx, dq, tc, id, tm, mh]	41	Advanced	Display Results | More
42	"walking meditation".mp. [mp=ti, ab, hw, tn, ot, dm, mf, dv, kw, fx, dq, tc, id, tm, mh]	60	Advanced	Display Results | More
43	"mindful stretching".mp. [mp=ti, ab, hw, tn, ot, dm, mf, dv, kw, fx, dq, tc, id, tm, mh]	2	Advanced	Display Results | More
44	"yoga".mp. [mp=ti, ab, hw, tn, ot, dm, mf, dv, kw, fx, dq, tc, id, tm, mh]	13050	Advanced	Display Results | More
45	"standing yoga".mp. [mp=ti, ab, hw, tn, ot, dm, mf, dv, kw, fx, dq, tc, id, tm, mh]	6	Advanced	Display Results | More
46	"mindful yoga".mp. [mp=ti, ab, hw, tn, ot, dm, mf, dv, kw, fx, dq, tc, id, tm, mh]	55	Advanced	Display Results | More
47	"hatha yoga".mp. [mp=ti, ab, hw, tn, ot, dm, mf, dv, kw, fx, dq, tc, id, tm, mh]	458	Advanced	Display Results | More
48	"mindful lying down yoga".mp. [mp=ti, ab, hw, tn, ot, dm, mf, dv, kw, fx, dq, tc, id, tm, mh]	0	Advanced	Save | More
49	"lying down yoga".mp. [mp=ti, ab, hw, tn, ot, dm, mf, dv, kw, fx, dq, tc, id, tm, mh]	1	Advanced	Display Results | More
50	"sounds and thoughts".mp. [mp=ti, ab, hw, tn, ot, dm, mf, dv, kw, fx, dq, tc, id, tm, mh]	5	Advanced	Display Results | More
51	"mindfulness of sounds and thoughts".mp. [mp=ti, ab, hw, tn, ot, dm, mf, dv, kw, fx, dq, tc, id, tm, mh]	0	Advanced	Save | More
52	"working with difficulty meditation".mp. [mp=ti, ab, hw, tn, ot, dm, mf, dv, kw, fx, dq, tc, id, tm, mh]	0	Advanced	Save | More
53	"exploring difficulty meditation".mp. [mp=ti, ab, hw, tn, ot, dm, mf, dv, kw, fx, dq, tc, id, tm, mh]	0	Advanced	Save | More
54	"befriending".mp. [mp=ti, ab, hw, tn, ot, dm, mf, dv, kw, fx, dq, tc, id, tm, mh]	542	Advanced	Display Results | More
55	"mindful breathing".mp. [mp=ti, ab, hw, tn, ot, dm, mf, dv, kw, fx, dq, tc, id, tm, mh]	126	Advanced	Display Results | More
56	"breath awareness".mp. [mp=ti, ab, hw, tn, ot, dm, mf, dv, kw, fx, dq, tc, id, tm, mh]	105	Advanced	Display Results | More
57	"observing thought".mp. [mp=ti, ab, hw, tn, ot, dm, mf, dv, kw, fx, dq, tc, id, tm, mh]	5	Advanced	Display Results | More
58	1 or 2 or 3 or 4 or 5 or 6 or 7 or 8 or 9 or 10 or 11	5648410	Advanced	Display Results | More
59	12 or 13 or 14 or 15 or 16 or 17 or 18 or 19 or 20 or 21 or 22 or 23 or 24 or 25 or 26 or 27 or 28 or 29 or 30 or 31 or 32 or 33 or 34 or 35 or 36 or 37 or 38 or 39 or 40 or 41 or 42 or 43 or 44 or 45 or 46 or 47 or 48 or 49 or 50 or 51 or 52 or 53 or 54 or 55 or 56 or 57	83102	Advanced	Display Results | More
60	58 and 59	17105	Advanced	Display Results | More
61	limit 60 to human	13269	Advanced	Display Results | More
62	limit 61 to yr="1979 -Current"	13215	Advanced	Display Results | More

(3) Scopus

Search: TITLE-ABS (*mechanism**) OR TITLE-ABS (*mediat**) OR TITLE-ABS (*moderat**) OR TITLE-ABS (*practice-specific*) OR TITLE-ABS (*training-specific*) OR TITLE-ABS (*dismant**) OR TITLE-ABS (*"active ingredient"*) OR TITLE-ABS (*"active ingredients"*) OR TITLE-ABS (*"indirect effect"*) OR TITLE-ABS (*"indirect effects"*) AND TITLE-ABS (*mindful**) OR TITLE-ABS (*mindfulness*) OR TITLE-ABS (*mindfulness-based*) OR TITLE-ABS (*mbi*) OR TITLE-ABS (*mbp*) OR TITLE-ABS (*mbsr*) OR TITLE-ABS (*mbct*) OR TITLE-ABS (*meditation**) OR TITLE-ABS (*"sitting meditation"*) OR TITLE-ABS (*"formal practice"*) OR TITLE-ABS (*"home practice"*) OR TITLE-ABS (*"home-based practice"*) OR TITLE-ABS (*"formal practices"*) OR TITLE-ABS (*"home practices"*) OR TITLE-ABS (*"home-based practices"*) OR TITLE-ABS (*"body scan"*) OR TITLE-ABS (*"diaphragmatic breathing"*) OR TITLE-ABS (*"abdominal breathing"*) OR TITLE-ABS (*"belly breathing"*) OR TITLE-ABS (*"mindfulness of the breath"*) OR TITLE-ABS (*"mindfulness of breathing"*) OR TITLE-ABS (*"mindfulness of breath and body"*) OR TITLE-ABS (*"mindfulness of body and breath"*) OR TITLE-ABS (*"awareness of breathing"*) OR TITLE-ABS (*"breath and body"*) OR TITLE-ABS (*"breath meditation"*) OR TITLE-ABS (*"breathing-space"*) OR TITLE-ABS (*"stretch and breath"*) OR TITLE-ABS (*"mindful movement"*) OR TITLE-ABS (*"mindful walking"*) OR TITLE-ABS (*"walking meditation"*) OR TITLE-ABS (*"mindful stretching"*) OR TITLE-ABS (*"yoga"*) OR TITLE-ABS (*"standing yoga"*) OR TITLE-ABS (*"mindful yoga"*) OR TITLE-ABS (*"hatha yoga"*) OR TITLE-ABS (*"mindful lying down yoga"*) OR TITLE-ABS (*"lying down yoga"*) OR TITLE-ABS (*"sounds and thoughts"*) OR TITLE-ABS (*"mindfulness of sounds and thoughts"*) OR TITLE-ABS (*"working with difficulty meditation"*) OR TITLE-ABS (*"exploring difficulty meditation"*) OR TITLE-ABS (*"befriending"*) OR TITLE-ABS (*"mindful breathing"*) OR TITLE-ABS (*"breath awareness"*) OR TITLE-ABS (*"observing thought"*) AND (LIMIT-TO (EXACTKEYWORD , *"Human"*)) View less

(4) Cochrane Central

View fewer lines | Print

#1	MeSH descriptor: [Mindfulness] explode all trees	866
#2	mechanism*	60166
#3	mediat*	38971
#4	moderat*	117489
#5	practice-specific	56
#6	training-specific	95
#7	dismant*	200
#8	"active ingredient"	1250
#9	"active ingredients"	926
#10	"indirect effect"	607
#11	"indirect effects"	605
#12	mindful*	5965
#13	mindfulness-based	2765
#14	MBI	640
#15	MBP	725
#16	MBSR	778
#17	MBCT	517
#18	meditation*	3116
#19	"sitting meditation"	44
#20	"formal practice"	18
#21	"home practice"	509
#22	"home-based practice"	37
#23	"formal practices"	1
#24	"home practices"	24
#25	"home-based practices"	2
#26	"body scan"	211
#27	"diaphragmatic breathing"	303
#28	"abdominal breathing"	97
#29	"belly breathing"	2
#30	"mindfulness of the breath"	8
#31	"mindfulness of breathing"	8
#32	"mindfulness of the breath and body"	0
#33	"mindfulness of the body and breath"	0
#34	"awareness of breathing"	11
#35	"breath and body"	9
#36	"breath meditation"	11
#37	"breathing-space"	18
#38	"stretch and breath"	0
#39	"mindful movement"	35
#40	"mindful walking"	19

#41	"walking meditation"	30
#42	"mindful stretching"	5
#43	"yoga"	3668
#44	"standing yoga"	2
#45	"mindful yoga"	18
#46	"hatha yoga"	235
#47	"mindful lying down yoga"	0
#48	"lying down yoga"	0
#49	"sounds and thoughts"	4
#50	"mindfulness of sounds and thoughts"	0
#51	"working with difficulty meditation"	0
#52	"exploring difficulty meditation"	0
#53	"befriending"	112
#54	"mindful breathing"	50
#55	"breath awareness"	31
#56	"observing thought"	0
#57	#2 OR #3 OR #4 OR #5 OR #6 OR #7 OR #8 OR #9 OR #10 OR #11	198011
#58	OR #32 OR #33 OR #34 OR #35 OR #36 OR #37 OR #38 OR #39 OR #40 OR #41 OR #42 OR #43 OR #44 OR #45 OR #46 OR #47 OR #48 OR #49 OR #50 OR #51 OR #52 OR #53 OR #54 OR #55 OR #56	12189
#59	#57 AND #58	2848
#60	Type a search term or use the S or MeSH buttons to compose	N/A

Clear all

Highlight orphan lines

Supplement 4. References for included papers.

Cladder-Micus, M. B., van Aalderen, J., Donders, A. R. T., Spijker, J., Vrijsen, J. N., & Speckens, A. E. M. (2018). Cognitive reactivity as outcome and working mechanism of mindfulness-based cognitive therapy for recurrently depressed patients in remission. *Cognition and Emotion*, *32*(2), 371–378. https://doi.org/10.1080/02699931.2017.1285753

Collado-Navarro, C., Navarro-Gil, M., Pérez-Aranda, A., López-del-Hoyo, Y., Garcia-Campayo, J., & Montero-Marin, J. (2021). Effectiveness of mindfulness-based stress reduction and attachment-based compassion therapy for the treatment of depressive, anxious, and adjustment disorders in mental health settings: A randomized controlled trial. *Depression and Anxiety*, *38*(11), 1138–1151. https://doi.org/10.1002/da.23198

Dambrun, M., Berniard, A., Didelot, T., Chaulet, M., Droit-Volet, S., Corman, M., Juneau, C., & Martinon, L. M. (2019). Unified Consciousness and the Effect of Body Scan Meditation on Happiness: Alteration of Inner-Body Experience and Feeling of Harmony as Central Processes. *Mindfulness*, *10*(8), 1530–1544. https://doi.org/10.1007/s12671-019-01104-y

Dimidjian, S., Gallop, R., Levy, J., Beck, A., & Segal, Z. V. (2023). Mediators of change in online mindfulness-based cognitive therapy: A secondary analysis of a randomized trial of mindful mood balance. *Journal of Consulting and Clinical Psychology*, *91*(8), 496.

Fissler, M., Winnebeck, E., Schroeter, T., Gummersbach, M., Huntenburg, J. M., Gaertner, M., & Barnhofer, T. (2016). An Investigation of the Effects of Brief Mindfulness Training on Self-Reported Interoceptive Awareness, the Ability to Decenter, and Their Role in the Reduction of Depressive Symptoms. *Mindfulness*, *7*(5), 1170–1181. https://doi.org/10.1007/s12671-016-0559-z

Geurts, D. E. M., Schellekens, M. P. J., Janssen, L., & Speckens, A. E. M. (2021). Mechanisms of Change in Mindfulness-Based Cognitive Therapy in Adults With ADHD. *Journal of Attention Disorders*, *25*(9), 1331–1342. https://doi.org/10.1177/1087054719896865

Goldin, P. R., Morrison, A., Jazaieri, H., Brozovich, F., Heimberg, R., & Gross, J. J. (2016). Group CBT versus MBSR for social anxiety disorder: A randomized controlled trial. *Journal of Consulting and Clinical Psychology*, *84*, 427–437. https://doi.org/10.1037/ccp0000092

Hayes-Skelton, S. A., & Lee, C. S. (2020). Decentering in Mindfulness and Cognitive Restructuring for Social Anxiety: An Experimental Study of a Potential Common Mechanism. *Behavior Modification*, *44*(6), 817–840. https://doi.org/10.1177/0145445519850744

Hofheinz, C., Reder, M., & Michalak, J. (2020). How specific is cognitive change? A randomized controlled trial comparing brief cognitive and mindfulness interventions for depression. *Psychotherapy Research*, *30*(5), 675–691. https://doi.org/10.1080/10503307.2019.1685138

Hoge, E. A., Bui, E., Goetter, E., Robinaugh, D. J., Ojserkis, R. A., Fresco, D. M., & Simon, N. M. (2015). Change in Decentering Mediates Improvement in Anxiety in Mindfulness-Based Stress Reduction for Generalized Anxiety Disorder. *Cognitive Therapy and Research*, *39*(2), 228–235. https://doi.org/10.1007/s10608-014-9646-4

Lengacher, C. A. (2021). Mindfulness-based stress reduction for breast cancer survivors (MBSR(BC)): Evaluating mediators of psychological and physical outcomes in a large randomized controlled trial. *J Behav Med*, 14. https://doi.org/10.1007/s10865-021-00214-0

Lengacher, C. A., Shelton, M. M., Reich, R. R., Barta, M. K., Johnson-Mallard, V., Moscoso, M. S., Paterson, C., Ramesar, S., Budhrani, P., Carranza, I., Lucas, J., Jacobsen, P. B., Goodman, M. J., & Kip, K. E. (2014). Mindfulness based stress reduction (MBSR(BC)) in breast cancer: Evaluating fear of recurrence (FOR) as a mediator of psychological and physical symptoms in a randomized control trial (RCT). *J Behav Med*, 11. https://doi.org/10.1007/s10865-012-9473-6

Li, Y., Zhang, A. J., Meng, Y., Hofmann, S. G., Zhou, A. Y., & Liu, X. (2023). A randomized controlled trial of an online self-help mindfulness intervention for emotional distress: serial mediating effects of mindfulness and experiential avoidance. *Mindfulness*, *14*(3), 510-523.

Lönnberg, G., Jonas, W., Unternaehrer, E., Bränström, R., Nissen, E., & Niemi, M. (2020). Effects of a mindfulness based childbirth and parenting program on pregnant women's perceived stress and risk of perinatal depression–Results from a randomized controlled trial. *Journal of Affective Disorders*, *262*, 133–142. https://doi.org/10.1016/j.jad.2019.10.048

López-del-Hoyo, Y., Collado-Navarro, C., Pérez-Aranda, A., García-Campayo, J., López-Montoyo, A., Feliu-Soler, A., Luciano, J. V., & Montero-Marin, J. (2022). Assessing mindfulness and self-compassion facets as mediators of change in patients with depressive, anxious and adjustment disorders: Secondary data analysis of a randomized controlled trial. *Journal of Contextual Behavioral Science*, *24*, 171–178. https://doi.org/10.1016/j.jcbs.2022.05.007

Maloney, S., Montero-Marin, J., & Kuyken, W. (2023). Pathways to mental well-being for graduates of mindfulness-based cognitive therapy (MBCT) and mindfulness-based stress reduction (MBSR): A mediation analysis of an RCT. *Psychotherapy Research*, 1-12.

McManus, F., Surawy, C., Muse, K., Vazquez-Montes, M., & Williams, J. M. G. (2012). A randomized clinical trial of mindfulness-based cognitive therapy versus unrestricted services for health anxiety (hypochondriasis). *Journal of Consulting and Clinical Psychology*, *80*(5), 817. https://doi.org/10.1037/a0028782

Montero-Marin, J., Taylor, L., Crane, C., Greenberg, M. T., Ford, T. J., Williams, J. M. G., García-Campayo, J., Sonley, A., Lord, L., Dalgleish, T., Blakemore, S.-J., Team, M., & Kuyken, W. (2021). Teachers "finding peace in a frantic world": An experimental study of self-taught and instructor-led mindfulness program formats on acceptability, effectiveness, and mechanisms. *Journal of Educational Psychology*, *113*(8), 1689. https://doi.org/10.1037/edu0000542

Montero-Marin, J., Hinze, V., Maloney, S., van der Velden, AM., Hayes, R., Watkins, E., Byford, S., Dalgleish, T., & Kuyken, W. (under review). Examining What Works for Whom and How in Mindfulness-Based Cognitive Therapy (MBCT) for Recurrent Depression: A Moderated-Mediation Analysis in the PREVENT Trial.

Moreira, M. D. F., Gamboa, O. L., & Oliveira, M. A. P. (2023)[a]. Mindfulness intervention effect on endometriosis-related pain dimensions and its mediator role on stress and vitality: a path analysis approach. *Archives of Women's Mental Health*, 1-11.

Moreira, M. D. F., Gamboa, O. L., & Pinho Oliveira, M. A. (2023)[b]. Cognitive-affective changes mediate the mindfulness-based intervention effect on endometriosis-related pain and mental health: A path analysis approach. *European Journal of Pain*, *27*(10), 1187-1202.

Morrison, A. S., Mateen, M. A., Brozovich, F. A., Zaki, J., Goldin, P. R., Heimberg, R. G., & Gross, J. J. (2019). Changes in Empathy Mediate the Effects of Cognitive-Behavioral Group Therapy but Not Mindfulness-Based Stress Reduction for Social Anxiety Disorder. *Behavior Therapy*, *50*(6), 1098–1111. https://doi.org/10.1016/j.beth.2019.05.005

Nyklíček, I., Dijksman, S. C., Lenders, P. J., Fonteijn, W. A., & Koolen, J. J. (2014). A brief mindfulness based intervention for increase in emotional well-being and quality of life in percutaneous coronary intervention (PCI) patients: The MindfulHeart randomized controlled trial. *Journal of Behavioral Medicine*, *37*(1), 135–144. https://doi.org/10.1007/s10865-012-9475-4

Pérez-Aranda, A., Feliu-Soler, A., Montero-Marín, J., García-Campayo, J., Andrés-Rodríguez, L., Borràs, X., Rozadilla-Sacanell, A., Peñarrubia-Maria, M. T., Angarita-Osorio, N., McCracken, L. M., & Luciano, J. V. (2019). A randomized controlled efficacy trial of mindfulness-based stress reduction compared with an active control group and usual care for fibromyalgia: The EUDAIMON study. *PAIN*, *160*(11), 2508–2523. https://doi.org/10.1097/j.pain.0000000000001655

Sousa, G. M. D., Lima-Araújo, G. L. D., Araújo, D. B. D., & Sousa, M. B. C. D. (2021). Brief mindfulness-based training and mindfulness trait attenuate psychological stress in university students: a randomized controlled trial. *BMC psychology*, *9*, 1-14.

Spinhoven, P., Hoogerwerf, E., van Giezen, A., & Greeven, A. (2022). Mindfulness-based cognitive group therapy for treatment-refractory anxiety disorder: A pragmatic randomized controlled trial. *Journal of Anxiety Disorders*, *90*, 102599. https://doi.org/10.1016/j.janxdis.2022.102599

Sun, Y., Wang, J., Mao, F., Sun, J., Zhang, X., & Cao, F. (2022). Personalized exploration of mindfulness-based intervention on antenatal depression: Moderated mediation analyses of a randomized controlled trial. *Current Psychology*. https://doi.org/10.1007/s12144-022-03231-7

van Aalderen, J. R., Donders, A. R. T., Giommi, F., Spinhoven, P., Barendregt, H. P., & Speckens, A. E. M. (2012). The efficacy of mindfulness-based cognitive therapy in recurrent depressed patients with and without a current depressive episode: A randomized controlled trial. *Psychological Medicine*, *42*(5), 989–1001. https://doi.org/10.1017/S0033291711002054

van der Velden, A. M., Kuyken, W., O'Toole, M., Montero-Marin, J., Fjorback, L., & Roepstorff, A. (in press). *Change in decentering and mindfulness mediates the effect of Mindfulness-based cognitive therapy in the treatment of recurrent depression: A randomized controlled design*.

Villa, C. D., & Hilt, L. M. (2014). Brief Instruction in Mindfulness and Relaxation Reduce Rumination Differently for Men and Women. *International Journal of Cognitive Therapy*, *7*(4), 320–333. https://doi.org/10.1521/ijct_2014_07_02

Supplement 5. Content analysis of key MBP curricula for practice terms.

Practice Name	MBSR-FCL	Key Information	MBSR (2017)	Key Information	MBCT-D	Key Information	MBCT-FP	Key Information	Characteristics of Practice across curricula
**Body Scan**	√	(1)Session 1, 2, 3, 4, 5, 6 and 8<br><br>(2) Once a day for at least 6 days per week; 45 minutes each practice (Session 1 and 2) & At least 3 days per week; 45 minutes each practice (Session 3 and 4) & At least 3 days per week; 45 minutes each practice (Alternative to yoga; Session 5 and 6) & Two times during week; 45 minutes each practice (Session 8)<br><br>(3) Re-establishing contact with the body;	√	(1) Session 1, 2, 3, 4 and 6<br><br>(2) Once a day for at least 6 days per week; 45 minutes each practice (Session 1 and 2) & 3 out of 6 days per week; 45 minutes each practice (Session 3 and 4); 3 out of 6 days per week; 45 minutes each practice (Session 6; Alternating with yoga)<br><br>(3) Applying mindfulness to our life experientially; Opening and starting to explore; Acknowledging alternative perspectives;	√	(1)Session 1 and 2<br><br>(2) Once a day for 6 days per week; 40 minutes each practice<br><br>(3) Attention; Allowing things to be as they are; Experiential knowing, noticing and relating differently to mental states<br><br>(4) None	√	(1) Session 2<br><br>(2) Twice a day for 6 days per week; 15 minutes each practice<br><br>(3) Becoming aware of the 'doing mode'; Embracing the 'being mode'; Attention; Awareness of the body<br><br>(4) None	-Home-based<br><br>-Introduced at beginning of course for MBSR and MBCT<br><br>-Practised at least 3 days during week for sessions that implement this practice<br><br>-Scheduled with CD Guidance<br><br>-Dosage ranges from 15 minutes to 45 minutes

		Concentrating and flexibility of attention; Feeling and inhabiting each region of the body<br><br>(4) None		Trusting the possibility to see oneself with fresh eyes (Session 1 Theme); Perception and creative responding (Session 2 Theme); Being present (Session 3 Theme); Learning new ways to relate to our experience; Flexible attention (Session 4 Theme); Interpersonal mindfulness (Session 6 Theme)<br><br>4) None					
**<u>Breath: Sitting Meditation</u>**	√	(1)Session 1, 2, 3 and 4<br><br>(2) Once a day for at least 6 days per week; 10 minutes each practice (Sessions 1 and 2) & Once	√	(1)Session 2, 3, 4<br><br>(2) Once a day for at least 6 days per week; 10-15 minutes each practice (Session 2); Once a day for	√	1)Session 2 and 3<br><br>(2) Once a day for 6 days per week; 10 minutes each practice [*Mindfulness*	√	(1)Session 1, 3, 4 and 5<br><br>Important Note: In Session 1, *Mindfulness of Body and Breath* is introduced and	-Home-based<br><br>-Introduced at beginning of course for MBSR and MBCT<br><br>-Practised once a day during

		a day for at least 6 days per week; 15-20 minutes each practice (Sessions 3) & Once a day for at least 6 days per week; 30 minutes each practice<br><br>(3) Tuning into the here and now; Using the breath as an anchor of awareness; Calmness<br><br>(4) Awareness of breathing; Diaphragmatic breathing; Abdominal breathing; belly breathing		at least 6 days per week; 15-20 minutes each practice (Session 3); Once a day at least 6 days per week; 20 minutes each practice (Session 4)<br><br>(3) Perception and creative responding (Session 2 Theme); Being present (Session 3 Theme); Learning new ways to relate to our experience; Flexible attention (Session 4);<br><br>(4) Abdominal breathing; Sitting meditation; Awareness of breathing; AOB		*of Breath; Session 2*] & Once a day for 6 days per week; 30 minutes each practice [*Breath and Body; Session 3]* Important Note: First 10 minutes is *Mindfulness of Breath* from Session 2 & Once a day for 3 days per week; 33-40 minutes each practice [*Stretch and Breath*; Session 3] Important Note: The first 3-10 minutes are stretching and then 30 minutes of *Breath and Body.*<br><br>(3) Living in our heads; Single focus; Attention (Session 2) & Here and now; Anchoring;		practised and in Session 3, 4 and 5 *Breathe and Body* is introduced and practised. Both practices use the breath and have similar intentions.<br><br>(2) 2 times a day for 6 days per week; 8 minutes each practice (Session 1 and 4) & Once a day for 6 days per week; 8 minutes each practice (Session 3 and Session 5)<br><br>(3) Waking up to the auto-pilot<br><br>(4) Mindfulness of Body and Breath; Breathe and Body	week for sessions that implemented this practice<br><br>-Scheduled with CD guidance<br><br>-Dosage ranges from 8 minutes to 30 minutes

						Connecting to a broader perspective (Session 3) & Becoming more intimate with thought patterns; Relating to experience in different way (Session 3; *Stretch and Breath*)<br><br>(4) Sitting meditation; Mindfulness of breath(ing); Breath meditation; Stretch and Breath; Breath and Body			
**<u>Mindful Movement/Yoga</u>**	√	(1) 3, 4, 5, and 6<br><br>(2) Once a day for 3 days per week; 45 minutes each practice (Session 3, 4, 5 and 6);<br><br>(3) Being present	√	(1)Session 3, 4, 5 and 6<br><br>(2) At least 3 days per week; 45 minutes each practice (Session 3 and 4) & At least 3 days per week; 45 minutes each practice (Alternative to	√	(1)Session 3<br><br>(2) Once a day for 3 days per week; 40 minutes each practice<br><br>(3) Bringing awareness to the body; Recognizing striving;	√	(1)Session 3<br><br>(2) Once a day for 6 days per week; 8 minutes each practice<br><br>(3) Recognizing striving; Noticing limits	-Home-based<br><br>-Introduced in middle of course for MBSR and MBCT<br><br>-Practised at least 3 days per week for sessions that

		(Session 3 Theme); Learning new ways to relate to our experience; Flexible attention (Session 4); Responding versus reacting; Recognizing conditioned patterns (Session 5 Theme); Interpersonal mindfulness (Session 6 Theme)<br><br>(4) Standing mountain pose; Standing poses; Standing yoga; Mindful lying down yoga; Lying down yoga; Mindful yoga; Hatha yoga		*Body Scan*; Session 5 and 6) & New standing yoga and other postures introduced in Session 6<br><br>(3) Becoming aware of striving and forcing ; Accepting the body as we find it; Understanding and befriending our limits; Unifying body and mind<br><br>(4) Lying down yoga (Session 3 and 4); Standing yoga (Session 5)		Working with physical boundaries and accepting limits; Learning a new way to take care of ourselves<br><br>(4) Mindful stretching		(4) None	implemented this practice<br><br>-Scheduled with CD guidance<br><br>-Dosage ranges from 8 minutes to 45 minutes
**<u>Sounds and Thoughts: Sitting Meditation</u>**		(1)Session 5 and 6		(1)Session 5 and 6		(1)Session 4<br><br>(2) Once a day for 6 days		(1)Session 4 and 5	-Home-based<br><br>-Introduced towards the

	√	(2) Once a day for at least 3 days per week; 45 minutes each practice (Session 5 and 6)<br><br>(3) 'Paying attention to an expanding field of objects of attention: the breath, other body sensations; a sense of the body as a whole sitting and breathing, sounds, thoughts, and emotions and then a choiceless awareness of whatever is most vivid in your experience in the present moment' (pg. 54-74)<br><br>(4) Sitting meditation	√	(2) Once a day for at least 3 days per week; 45 minutes each practice (Session 5 and 6)<br><br>(3) Responding versus reacting; Recognizing conditioned patterns (Session 5 Theme); Interpersonal mindfulness (Session 6 Theme)<br><br>(4) Sitting meditation	√	per week; 30-40 minutes each practice<br><br>(3) Focusing attention; Expanding attention; Experiencing a more spacious awareness<br><br>(4) Sitting meditation	√	(2) Twice a day for 6 days per week; 8-minutes each practice (Session 4) & Once a day for 6 days per week; 8 minutes each practice (Session 5)<br><br>(3) Receiving and noticing; Attention<br><br>(4) Sounds and Thoughts Meditation; Mindfulness of Sounds and Thoughts	second half of the course in both MBSR and MBCT<br><br>-Practised at least 3 days during week for sessions that implemented this practice<br><br>-Scheduled with CD guidance<br><br>-Dosage ranges from 8 minutes to 45 minutes
**Working with Difficulty:**						(1)Session 5		(1)Session 5	-Home-based

**Sitting Meditation**	X	n/a	X	n/a	√	(2) Once a day for 3 days per week [guided with CD; running time 26 minutes each practice] and Once a day for 3 days per week [self-guided if preferable]; 30-40 minutes each practice<br><br>(3) Adopting a new relationship with unpleasant thoughts, feelings, and sensations<br><br>(4) Sitting meditation; Working with difficulty	√	(2) Once a day for 6 days per week; 10 minutes each practice<br><br>(3) Using the body to turn towards negativity<br><br>(4) Exploring Difficulty	-Scheduled with CD guidance<br><br>-Practised once a day for at least three days per week<br><br>-Dosage ranges from 10 to 40 minutes each practice.
**Scheduled Breathing Spaces**	X	n/a	X	n/a	√	(1)Session 3, 4, 5, 6 and 7<br><br>(2) Three times a day at regular times for 6 days per week; 3	√	(1)Session 3, 4, 5, 6 and 7<br><br>(2) Two to three times a day at regular times for 6 days per week; 3	-Home-based<br><br>-Scheduled with CD guidance<br><br>-Repeated throughout the course

						minutes [5 minutes running time] each practice (Session 3, 4, 5 and 6)<br><br>*Breathing space-Basic version (Sessions 3, 4, 5, 6)*<br><br>*Breathing space-Extra guidance (Session 5)*<br><br>(3) Learning how to apply what we learn in the formal practices in everyday life<br><br>(4) None		minutes each practice (Session 3, 4, 5, 6, 7)<br><br>(3) Helps bridge what we learn in formal practice to everyday; Helps punctuate the day; Maintain a compassionate and mindful stance; Allows you to see as things arise<br><br>(4) None	-Involves applying mindfulness to everyday activities<br><br>-Dosage ranges from 3-5 minutes<br><br>-Practised two to three times a day for 6 days per week
**Befriending: Sitting Meditation**	X	n/a	X	n/a	X	n/a	√	(1)Session 6<br><br>(2) Once a day for 6 days per week; 10 minutes each practice<br><br>(3) How to be kind to yourself	-Home-based<br><br>-Scheduled with CD guidance<br><br>-Practised once a day for 6 days per week

								(4) None	-Dosage is 10 minutes per practice

Key Information: (1) = Introduced When? (2) = Frequency/Duration (3) = Themes/Ingredients and (4) = Other names used to describe this practice. '√' indicates that the practice is present within this specific manual whereas 'X' indicated that the practice is not. 'MBSR-Full Catastrophe Living.' The book Full Catastrophe Living written by Jon Kabat-Zinn (2013; Second edition). 'MBSR (2017)'—Mindfulness-based Stress Reduction. The authorized curriculum guide was used (Santorelli, Meleo-Meyer, Koerbel, and Kabat-Zinn, 2017). 'MBCT-D'—Mindfulness-based Cognitive Therapy for Depression. The Manual used was written by Segal, Williams, and Teasdale (2018; second edition). 'MBCT-FP'—Mindfulness-based Cognitive Therapy-Finding Peace in a Frantic World. The Manual used was written by Williams and Penman (2011).

References:

Kabat-Zinn J. Full catastrophe living: using the wisdom of your body and mind to face stress, pain, and illness. New York: Bantam Books; 2013.

Santorelli, S. F., Kabat-Zinn, J., Blacker, M., Meleo-Meyer, F., & Koerbel, L. (2017). Mindfulness-based stress reduction (MBSR) authorized curriculum guide. *Center for mindfulness in medicine, health care, and society (CFM). University of Massachusetts Medical School*.

Segal Z, Williams M, Teasdale J. Mindfulness-Based Cognitive Therapy for Depression, Second Edition. Guilford Publications; 2018. 473 p.

Williams, M., & Penman, D. (2011). *Mindfulness: A practical guide to finding peace in a frantic world*. Hachette UK.

Supplement 6. Visual depiction of indirect effect and relevant paths (a, b, c, and c').

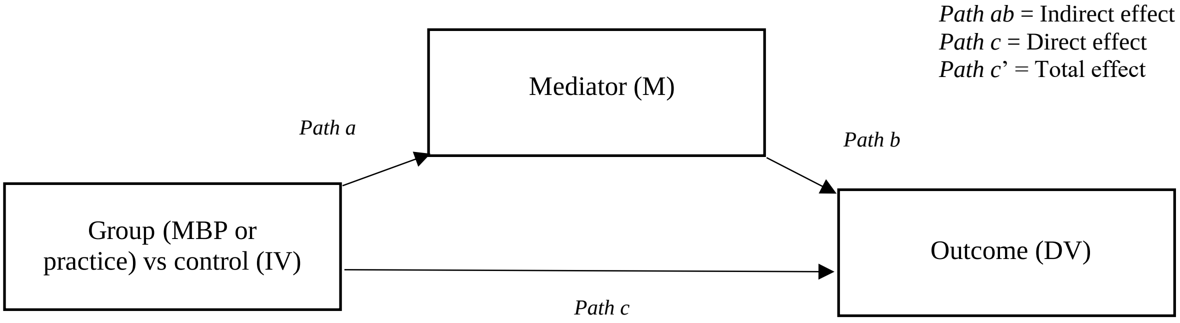

This figure aims to depict how to test the indirect effect (the product of path a and b; path ab). Path c is the direct effect between the IV (MBP or practice versus control group) and the DV (outcome). Path c' is the total effect and represents the product of paths a, b, and c. Note. IV = independent variable; DV = dependent variable; MBP = mindfulness-based programme; M = mediator.

Supplement 7. Zhao et al. (2010) and Kazdin (2007) recommendations for establishing a mechanism of change.

All included papers reported *path ab* (the IE), since this was a key inclusion criterion for establishing mediation. However, to further address the quality of mediation reporting, we examined additional criteria: (1) reporting the significance of the direct effect (DE; *path c*) after controlling for the IE; and (2) reporting the signs of the IE, and DE after controlling for the IE, independently of the sign of the total effect (*path c'*). This information is required to classify the type of mediation as complementary, competitive, indirect-only, direct-only effect, or no effect. In addition to addressing quality of testing and reporting following the Zhao et al. (2010) criteria, additional criteria was assessed (Kazdin, 2007). The '*plausibility or coherence*', which looks at the extent to which the broader literature supports the likelihood of the proposed mechanism was addressed by determining whether the included study used theory to articulate and operationalize hypotheses. The '*timeline*', which argues that change in the proposed mechanism should occur before change in outcome was explored by considering whether the included study implemented different time-point assessments for the proposed mediator and outcome. In the context of MBPs, given that the amount of mindfulness practice (dosage) is arguably one medium through which change occurs (Parsons et al., 2017), the extent to which the study established (a) an association between the amount of practice completed and change in the proposed mediator and (b) an association between the amount of practice completed and change in outcome ['*gradient' criterion*] was also examined.

References:

Kazdin, A. E. (2007). Mediators and mechanisms of change in psychotherapy research. *Annu. Rev. Clin. Psychol.*, *3*, 1-27.

Parsons, C. E., Crane, C., Parsons, L. J., Fjorback, L. O., & Kuyken, W. (2017). Home practice in mindfulness-based cognitive therapy and mindfulness-based stress reduction: a systematic review and meta-analysis of participants' mindfulness practice and its association with outcomes. *Behaviour research and therapy*, *95*, 29-41.

Zhao, X., Lynch Jr, J. G., & Chen, Q. (2010). Reconsidering Baron and Kenny: Myths and truths about mediation analysis. *Journal of consumer research*, *37*(2), 197-206.

Supplement 8. Further details on inclusion and exclusion criteria.

A specific year range (1979 and onwards), which coincides with the introduction of MBPs in mainstream settings, was considered to help isolate studies that concern an MBP or a formal mindfulness practice. Informal mindfulness practices (e.g., used to cultivate mindfulness in daily activities) or practices that did not tap into the terms identified from the content analysis (Supplement 5) were excluded. Due to the wide range of yoga terms, only studies that specified hatha yoga were included since this type of yoga was specifically used in MBSR curricula. Other mindfulness-informed programmes (e.g., Acceptance and Commitment Therapy, Dialectical Behaviour Therapy, or Mindful Self-Compassion) were excluded because sustained mindfulness training is not a core ingredient, and second-generation mindfulness programmes, which focus on active awareness of phenomena that is spiritual in nature, were excluded because the evidence-base is still in its very early stages. Formal practices were prioritized over informal practices and other practice-related components (e.g., silent retreats, orientation sessions, in-class practices) because they are easier to operationally define in terms of the dosage and guidance. In an effort to obtain evidence from different geographical locations and to optimise the primary languages of the research team, both English and Spanish publications were prioritized.

Supplement 9. Further details on methodology for evidence gap map development.

These evidence gap maps were developed by first identifying all proposed psychological mechanisms and outcomes from the included studies. One reviewer (SM) first identified the measurements used to assess each mechanism and outcome and then the items within each measurement were reviewed and mapped onto a higher-order category. Using the constant comparative method (Glaser & Strauss, 2017), a second reviewer (YS) made their classification for each measure and then any discrepancies were discussed until a common conceptual denomination for each measure was reached. Any conflicts were resolved by a third reviewer (JMM, MK, and WK) and additional reviewers outside the internal research team were consulted to evaluate the final list of measures, in relation to each higher-order category, to help reach a consensus (RB and AV). Six unique categories for the proposed psychological mechanisms were identified: mindfulness skills; attention and awareness; decentering; attitudes of mindfulness; emotional regulation; and dysfunctional thoughts, attitudes, and behaviours. Three higher-order categories were identified for the outcomes: mental health conditions; mental languishing; and well-being.

Existing theory (Feldman & Kuyken, 2019; Greco et al., 2011; Segal et al., 2018; Shapiro et al., 2006) and past research (Alsubaie et al., 2017; Gu et al., 2015; Maddock & Blair, 2021; van der Velden et al., 2015) on the mechanisms of MBPs helped inform how these categories were defined. The outcomes under mental health conditions include: symptoms of anxious, depressive, and adjustment disorders; psychosomatic disorders; and behavioural disorders. The outcomes pertaining to mental health conditions concerned symptoms for individuals with a diagnosed mental health condition. The mental languishing outcomes included: mental health symptoms more broadly (e.g., depression and anxiety); stress and burnout. To be distinguished from mental health conditions, mental health symptoms can be experienced by the entire population distribution. Stress and burnout were also included under this category as they tend to be associated with mental health symptoms (Maslach, 1996). The well-being category included outcomes relating to the emotional and psychological dimensions of well-being and related constructs (e.g., positive affect and states of mind). However, other dimensions of well-being and quality of life were explored that tapped into social, physical, and environmental dimensions. The extant literature (Huppert & So, 2013; Patel et al., 2018; Rose, 2008; Salvador-Carulla et al., 2014) on mental health and public health helped inform how these terms were defined. Existing theory (Arango et al., 2018; Greenberg & Abenavoli, 2017; World Health Organization, 2002) was used to help categorize and define different mental health strategies (i.e., treatment, prevention, and promotion). A random sample (n = 4) of corresponding authors (15% of total) were contacted to determine the extent to which they agreed with the category chosen for the type of mental health strategy and all contacted authors agreed with the proposed categorization. For the types of active comparators, five higher-order categories were identified: (1) treatment-as-usual (TAU); (2) stress

management/relaxation [SM/R]; (3) cognitive (behavioural) intervention [CBI]; (4) attentional control [AC]; and (5) self-help mindfulness [SH-M]. To help draw a conceptual boundary between the SH-M comparators and the papers that evaluated one type of mindfulness practice, as one key subcomponent of an MBP, the SH-M comparators included a range of practice types and included additional components (e.g., psychoeducation) whereas the papers that evaluated one type of mindfulness practice did not. The SH-M comparators are generally examined to understand the role of a teacher-led versus self-led format (i.e., delivery mode) whereas the papers that evaluate one type of mindfulness practice aim to understand its role in targeting key mechanisms of change outside the framework of the entire MBP. The conceptual mapping of the measures are described below in Supplement 10, along with definitions for the different types of mental health strategies in Supplement 11 .

References

Alsubaie, M., Abbott, R., Dunn, B., Dickens, C., Keil, T. F., Henley, W., & Kuyken, W. (2017). Mechanisms of action in mindfulness-based cognitive therapy (MBCT) and mindfulness-based stress reduction (MBSR) in people with physical and/or psychological conditions: A systematic review. *Clinical Psychology Review*, *55*, 74–91. https://doi.org/10.1016/j.cpr.2017.04.008

Arango, C., Díaz-Caneja, C. M., McGorry, P. D., Rapoport, J., Sommer, I. E., Vorstman, J. A., McDaid, D., Marín, O., Serrano-Drozdowskyj, E., Freedman, R., & Carpenter, W. (2018). Preventive strategies for mental health. *The Lancet Psychiatry*, *5*(7), 591–604. https://doi.org/10.1016/S2215-0366(18)30057-9

Feldman, C., & Kuyken, W. (2019). *Mindfulness: Ancient wisdom meets modern psychology*. The Guilford Press.

Glaser, B. G., & Strauss, A. L. (2017). *The discovery of grounded theory: Strategies for qualitative research*. Routledge.

Greco, L. A., Baer, R. A., & Smith, G. T. (2011). Assessing mindfulness in children and adolescents: Development and validation of the Child and Adolescent Mindfulness Measure (CAMM). *Psychological Assessment*, *23*(3), 606.

Greenberg, M. T., & Abenavoli, R. (2017). Universal Interventions: Fully Exploring Their Impacts and Potential to Produce Population-Level Impacts. *Journal of Research on Educational Effectiveness*, *10*(1), 40–67. https://doi.org/10.1080/19345747.2016.1246632

Gu, J., Strauss, C., Bond, R., & Cavanagh, K. (2015). How do mindfulness-based cognitive therapy and mindfulness-based stress reduction improve mental health and wellbeing? A systematic review and meta-analysis of mediation studies. *Clinical Psychology Review*, *37*, 1–12. https://doi.org/10.1016/j.cpr.2015.01.006

Huppert, F. A., & So, T. T. C. (2013). Flourishing Across Europe: Application of a New Conceptual Framework for Defining Well-Being. *Social Indicators Research*, *110*(3), 837–861. https://doi.org/10.1007/s11205-011-9966-7

Maddock, A., & Blair, C. (2021). How do mindfulness-based programmes improve anxiety, depression and psychological distress? A systematic review. *Current Psychology*. https://doi.org/10.1007/s12144-021-02082-y

Maslach, C. (1996). Maslach burnout inventory-human services survey (MBI-HSS). *MBI Manual*, 192–198.

Patel, V., Saxena, S., Lund, C., Thornicroft, G., Baingana, F., Bolton, P., Chisholm, D., Collins, P. Y., Cooper, J. L., Eaton, J., Herrman, H., Herzallah, M. M., Huang, Y., Jordans, M. J. D., Kleinman, A., Medina-Mora, M. E., Morgan, E., Niaz, U., Omigbodun, O., … UnÜtzer, Jü. (2018). The Lancet Commission on global mental health and sustainable development. *The Lancet*, *392*(10157), 1553–1598. https://doi.org/10.1016/S0140-6736(18)31612-X

Rose, G. A. (2008). *Rose's strategy of preventive medicine: The complete original text*. Oxford University Press.

Salvador-Carulla, L., Lucas, R., Ayuso-Mateos, J. L., & Miret, M. (2014). Use of the terms" Wellbeing" and" Quality of Life" in health sciences: A conceptual framework. *The European Journal of Psychiatry*, *28*(1), 50–65.

Segal, Z., Williams, M., & Teasdale, J. (2018). *Mindfulness-Based Cognitive Therapy for Depression, Second Edition*. Guilford Publications.

Shapiro, S. L., Carlson, L. E., Astin, J. A., & Freedman, B. (2006). Mechanisms of mindfulness. *Journal of Clinical Psychology*, *62*(3), 373–386. https://doi.org/10.1002/jclp.20237

van der Velden, A. M., Kuyken, W., Wattar, U., Crane, C., Pallesen, K. J., Dahlgaard, J., Fjorback, L. O., & Piet, J. (2015). A systematic review of mechanisms of change in mindfulness-based cognitive therapy in the treatment of recurrent major depressive disorder. *Clinical Psychology Review*, *37*, 26–39. https://doi.org/10.1016/j.cpr.2015.02.001

World Health Organization. (2002). *Prevention and promotion in mental health* (What Is Prevention and Promotion in Mental Health?).

Supplement 10. Conceptual mapping of measures into categories of mechanisms and outcomes.

Mechanisms		
**Proposed category**	**Operational definition of category**	**Measures included in review that map onto category**
Mindfulness	Mindfulness can be described in general as a multidimensional construct that refers to a range of interrelated psychological, behavioural, and social skills (Feldman & Kuyken, 2019; Greco, Baer, & Smith, 2011). There is generally a lack of consensus about how the dimensions of mindfulness should be defined and operationalized (Baer et al., 2008). In the current review, mindfulness will be defined as a multidimensional construct that includes dimensions or interrelated skills (e.g., attention and awareness, attitudes of mindfulness, and decentering) which can be appreciated as overlapping yet distinct processes.<br><br>Theoretical models (Feldman & Kuyken, 2019; Z. Segal et al., 2018; Shapiro et al., 2006) argue that mindfulness involves bringing attention and awareness intentionally to the present-moment experience (e.g., thoughts, emotions, bodily sensations, behaviours) with attitudes such as acceptance, compassion, and curiosity. In the process of increasing one's attention and awareness, along with these attitudes, this then allows the individual to take a wider perspective (increasing one's decentering or meta-cognitive awareness) which increases one's discernment to make an empowered choice on how to respond to both internal and external stimuli.	Measures we believe map onto this multidimensional definition of mindfulness include the total score of **The Five Facet Mindfulness Questionnaire** [FFMQ], **The Five Facet Mindfulness Questionnaire-Short Form** [FFMQ-SF], **The Kentucky Inventory of Mindfulness** [KIMS], and **The Freiburg Mindfulness Inventory** [FMI], and the mindfulness subscale score of the **Self-Compassion Scale** [SCS]. The FFMQ, FFMQ-SF, and KIMS include four to five facets of mindfulness whereas the FMI-s includes a general factor of mindfulness. The mindfulness subscale of the SCS looks at mindfulness as a facet of self-compassion. The FFMQ and FFMQ-SF included five facets (observing, acting with awareness, describing, non-reactivity, and non-judgement), whereas the KIMS only includes four (observe, describe, act with awareness, and accept without judgement). The FFMQ-SF is a shorter version of the FFMQ. An item example for the **observing subscale of the FFMQ/FFMQ-SF** includes: 'When I take a shower or a bath, I stay alert to the sensations of water on my body'. An item example for the **acting with awareness subscale of the FFMQ/FFMQ-SF** includes: 'I don't pay attention to what I'm doing because I'm daydreaming, worrying or otherwise distracted' [reverse scored]. An item example for the **describing subscale of the FFMQ/ FFMQ-SF** includes: 'I'm good at finding the words to describe my feelings.' An item example for the **non-reactivity subscale for the FFMQ/FFMQ-SF** includes: 'When I have distressing thoughts or images, I 'step back' and am aware of the though or image without getting taken over by it'. An item example for the **non-judgement subscale of the FFMQ/ FFMQ-SF** includes: 'I believe that some of my thoughts are abnormal or bad and I shouldn't think that way' [reverse scored]. An item example for the **observe subscale of the KIMS** includes: 'I notice changes in my body, such as whether my breathing slows down or speeds up.' An item example for the **act with awareness subscale of the KIMS** includes: 'When I do things, my mind wanders off and I'm easily distracted'. An item example for the **describe subscale of the KIMS** includes: 'I'm good at finding the words to describe my feelings'. An item example of the **accept without judgement subscale of the KIMS** includes: 'I criticize myself for having irrational or inappropriate emotions' [reverse scored]. Item example of the **FMI-s** include: 'I am open to the experience of the present moment' and 'I am able to appreciate myself'. Item examples of the **mindfulness subscale of the SCS** include: 'When something upsets me I try to keep

		my emotions in balance' and 'When something painful happens I try to take a balanced view of the situation'.
Attention and awareness	Attention and awareness can be viewed as a dimension of mindfulness but also as distinct processes that broadly refer to our capacity to choose what we focus on. Attention and awareness is the doorway to experience (which can be broken down into the experience of bodily sensations, emotions, thoughts, and behaviours). There are different types of attention (e.g., alerting [scanning the environment to see what is there], orienting [turning our attention towards something in particular], executive attention [making a choice of what to do with what you have decided to attend to], and attentional control [a key feature of self-control which allows us to switch tasks]) (Feldman & Kuyken, 2019).	The measures that were considered related to attention and awareness include the acting with awareness subscale of the **FFMQ,** the Body/Self Phenomenology [**B/SP**] questions from Dambrun et al. (2019), the attention focusing and shifting subscales of **the Attentional Control Scale** [ACS], and the attention regulation, self-regulation, body listening, and trusting subscales of the **Multidimensional Assessment of Interoceptive Awareness** [MAIA], the total score of the **BRIEF-A**, and the **mind wandering** questions from Dambrun et al. (2019). An item example for the **acting with awareness subscale of the FFMQ** includes: 'I don't pay attention to what I'm doing because I'm daydreaming, worrying or otherwise distracted' [reverse scored]. The **B/SP** questions from Dambrun et al. (2019) includes items such as: 'I noticed that my body sensations changed at every moment', which focuses on the awareness of internal states specifically related to the body. The **attention focusing and shifting subscales of the ACS** include items such as: 'It's very hard for me to concentrate on a difficult task when there are noises around' (focusing) and 'I can quickly switch from one task to another' (shifting). The **attention regulation, self-regulation, body listening, and trusting subscales of the MAIA** include items such as: 'I can pay attention to my breath without being distracted by things happening around me' (attention regulation); 'When I feel overwhelmed I can find a calm place inside' (self-regulation); 'I listen for information from my body about my emotional state' (body listening); and 'I am at

		home in my body' (trusting). All subscales of the MAIA aim to capture aspects of interoceptive awareness, which relates to the ability to consciously sense, interpret, and integrate signals from within the body and providing a moment-to-moment mapping of the internal environment. The **BRIEF-A** is a measure of executive function and includes nine subscales (inhibit, shift, emotional control, self-monitor, initiate, working memory, plan/organise, task monitor, and organisation of materials. All nine subscales are summed up to calculate a total composite score. Item examples include: 'I need to be reminded to begin a task even when I am willing' (initiate); 'I have trouble concentrating on tasks such as chores, reading, or work' (working memory); and 'I get overwhelmed by large tasks' (plan/organize). Higher scores indicate poor executive function. For the **mind wandering** questions from Dambrun et al. (2019), participants were asked to indicate the extent (scale of 0-100) in which they were preoccupied with past things, future things, and things from the present. Mind-wandering occurs when individuals are distracted by their present moment experience and, therefore, their attention is elsewhere. Given that some items are phrased in the positive direction and others are phrased in the negative direction (e.g., the mind wandering questions), the sign of these relationships (negative vs. positive) are tracked in the main table summarizing the eligible studies to help manage this.
Attitudes	Attitudes can also be viewed as a dimension of mindfulness and also as a distinct process. For this review, attitudes of mindfulness refer to the attitudinal landscape which allows individuals to turn towards reactivity and work with it. Attitudes such as befriending, compassion, joy, and equanimity can be viewed as a dimension of mindfulness (Feldman & Kuyken, 2019). However, these attitudes can also be viewed as distinct from mindfulness. Befriending refers to the tendency to approach one's experience with a sense of curiosity, friendliness, and kindness. Compassion refers to the ability to recognize pain and the universality of pain in the human experience and to meet this with kindness, empathy, equanimity, and patience. Joy refers to the ability to appreciate and to experience gratitude and contentment. Equanimity refers to the experience of inner balance with a sense of awareness, care, and compassion (Feldman & Kuyken, 2019). Ultimately, these attitudes of mindfulness are also inherently overlapped.	The measures that were considered related to this attitudinal landscape include the accept without judgement subscale of the **KIMS**, the total score of the **Self-Compassion Scale** [SCS] **and the Self-Compassion Scale-Short Form** [SCS-SF], the self-kindness and common humanity subscales of the **SCS**, and the **Harmony** questions from Dambrun et al. (2019), and the **positive affective empathy** questions from Morrison et al. (2019). Item examples of the **accept without judgement subscale of the KIMS** include: 'I criticize myself for having irrational or inappropriate emotions' [reverse scored]. The SCS/SCS-SF include six subscales (self-kindness, self-judgement, common humanity, isolation, mindfulness, and over-identification). The self-judgement, isolation, and over-identification subscales serve as reverse-coded items of the self-kindness, common humanity, and mindfulness subscales. Item examples of the **self-kindness/self-judgement subscales of the SCS/SCS-SF** include: 'I try to be understanding and patient towards those aspects of my personality I don't like' (self-kindness) and 'I am disapproving and judgemental about my own flaws and inadequacies' (self-judgement). Item examples of the **common humanity/isolation subscales of the SCS/SCS-SF** include: 'I try to see my failings as part of the human condition' (common humanity) and 'When I'm feeling down, I tend to feel like most other people are probably happier than I am' (isolation). Item examples of the **mindfulness/over-identification subscales of the SCS/SCS-SF** include: 'When

		something painful happens I try to take a balanced view of the situation' (mindfulness) and 'When I fail at something important to me I become consumed by feelings of inadequacy' (over-identification). The total scores of the SCS/SCS-SF include all items of these six subscales. The **harmony** questions from Dambrun et al. (2019) included items such as: 'I feel that there is a certain harmony between what I am deep within me and my present life'. The **empathy task** [positive affective empathy] from Morrison et al. (2019) involves videotapes of participants describing positive and negative events and then participants rate these videos using a Likert scale [1 (very negative) to 9 (very positive) to assess their empathetic response.
Decentering	Decentering can also be viewed as a dimension of mindfulness and also as a distinct process. Decentering (or metacognition) allows an individual to see an experience from a different perspective and to view thoughts as merely mental events (Feldman & Kuyken, 2019; Fresco et al., 2007) and not necessarily reflective of reality.	Measures that were considered related to decentering include **The Toronto Mindfulness Scale** [TMS; decentering subscale] and the **Experiences Questionnaire** [EQ; decentering subscale]. The **TMS [decentering subscale]** includes items such as: 'I experienced myself as separate from my changing thoughts and feelings'. The **EQ [wider experiences/decentering subscale]** includes items such as: 'I can observe unpleasant feelings without being drawn into them'.
Dysfunctional thoughts, attitudes, and behaviours	Dysfunctional thoughts, attitudes, and behaviours refer to a wide range of maladaptive coping strategies or methods that individuals use to try to reduce their anxiety, depression, and stress. These negative coping strategies contrast with the mindfulness skills mentioned above, which can fall under positive coping strategies more broadly. Dysfunctional thoughts, attitudes, and behaviours may be viewed as a more 'distal' mechanisms that may decrease as a result of increased mindfulness skills (i.e., proximal mechanisms). In other words, these processes may change later along the temporal pathway whereas processes relating to mindfulness and interrelated skills (e.g., self-compassion, decentering) may change earlier on the temporal pathway and, therefore, may represent more 'proximal' mechanisms. However, this is currently an underexplored area of research and outside the scope of this review and, ultimately, warrants further investigation.	The measures that were considered related to dysfunctional thoughts, attitudes, and behaviours include the **Penn State Worry Questionnaire** [PSWQ], the brooding subscale of **The Ruminative Response Scale** [RRS], the **Leiden Index of Depression Sensitivity Revised** [LEIDS-R], the **negative self-statement subscale of the Automatic Thoughts Questionnaire Revised** [ATQ-R], the performance evaluation and approval by others subscales of the **Dysfunctional Attitude Scale** [DAS], **state rumination** [SR] questions in Villa & Hilt (2014), the **Subtle Avoidance Frequency Examination** [SAFE], the **Rumination on Sadness Scale** [RSS], the fear of recurrence problems and fear of recurrence concerns items of the **Concerns about Recurrence Scale** [CARS], the **Psychological Inflexibility in Pain Scale** [PIPS], the **Cognitive Distortions Questionnaire** [CD-Quest], the **Acceptance and Action Questionnaire ([AAQ-II], Pain Catastrophizing Scale [PCS], the McGill Pain Questionnaire [MPQ; affective dimension], and the PANAS-SF [negative affect ; NA] .** The **PSWQ** evaluates worry in the context of Generalized Anxiety Disorder (GAD) and includes items such as: 'My worries overwhelm me'. The **brooding subscale of the RRS** includes items such as 'Think about how alone you feel'. The **LEIDS-R** aims to evaluate cognitive reactivity and an item example includes: 'I can only think positive when I am in a good mood'. The **negative self-statement subscales of the ATQ-R** includes items such as 'Why can't I ever succeed'. The **performance evaluation and approval by others subscales of the DAS** include items such as 'People will probably think less of me if I make a mistake' (performance evaluation) and 'If others dislike you, you cannot

		be happy' (approval by others). The **SR** questions from Villa & Hilt (2014) ask respondents to rate to what extent they were focusing on their feelings and problems on a scale from 0 (not at all) to 10 (extremely). The **SAFE** questionnaire assesses the frequency of safety behaviours such as 'rehearse sentences in your mind''. The **RSS** includes items such as 'repeatedly thinking about what sadness is by concentrating on feelings and try to understand them'. An item example of the **CRS** includes: 'my sense of sexuality would be threatened by a recurrence of breast cancer'. The **PIPS** aims to assess psychological inflexibility in the context of chronic pain and includes items such as: 'I would do almost anything to get rid of my pain'. The **CD-Quest** assesses the frequency and intensity of maladaptive cognitions. An item example includes: 'I disqualify positive experiences or events insisting that they do not count'. An item example for the **AAQ-II** includes: "I am afraid of my feelings." An item from **PCS** includes: "When I feel pain, I feel like I can't go on." The questions relating to **MPQ [affective dimension]** relate to the fear, depression, and anxiety related to pain. An item example from **PANAS-SF [negative affect; NA]** includes: In the last two weeks have you felt "nervous".
Emotional regulation	Emotional regulation refers to a broad range of psychological processes responsible for monitoring, evaluating, and modifying emotional reactions (Thompson, 1991). These positive coping strategies may overlap with the mindfulness skills mentioned above. However, for this review, we have appreciated these processes as distinct. Emotional regulation may also be viewed as a more distal mechanism that may increase as a result of increased mindfulness skills (i.e., more proximal mechanisms).	The measures that were considered related to emotional regulation include the **emotional stability** questions from Dambrun et al. (2019), the reappraisal frequency and self-efficacy subscales of the **Emotion Regulation Questionnaire** [ERQ], the **Difficulties in Emotion Regulation Scale** [DERS], the **positive subscales of the Automatic Thoughts Questionnaire Revised [ATQ-R**], and **PANAS-SF [positive affect; PA].** The **emotional stability** questions from Dambrun et al. (2019) were taken from the neuroticism factor from the French Big Five Inventory and were scored in the reverse direction to measure emotional stability. Items from the neuroticism factor include: 'Is easily anxious'. An extended version of the **ERQ** was implemented by Goldin et al. (2016) to examine cognitive reappraisal frequency and cognitive reappraisal self-efficacy. An item example include: 'I control my emotions by changing the way I think about the situation I am in'. Higher scores on the ERQ were related to higher levels of emotional regulation. The **DERS** includes items such as: 'I experience my emotions as overwhelming and out of control' and 'I have no idea how I am feeling.' The **positive subscales (well-being and self-confidence) of the ATQ-R** include items such as 'I am proud of myself'. Given that some items are phrased in the positive direction and others are phrased in the negative direction, the signs (positive vs negative) are tracked in the main table summarizing the eligible studies. An item example for **PANAS [PA]** includes: In the last two weeks have you felt "alert".
Outcomes		

Proposed category	Operational definition of category	Measures included in review that map onto category
Mental health conditions	According to the World Health Organization (WHO), mental disorders refer to a 'clinically significant disturbance in an individual's cognition, emotional regulation, or behaviour' (WHO, 2022). It was estimated in 2019 that around 1 in every 8 individuals had a mental disorder. Common mental health disorders include anxiety and depression. Anxiety disorders are associated with 'excessive fear and worry and behavioural disturbances related to this' (WHO, 2022). Depression includes feelings of sadness, irritability, and emptiness. However, these symptoms differ from non-clinical levels of low mood which can be experienced by a wider distribution of the population. For this review, we have included constructs that relate to symptoms of mental disorder and are therefore evaluated in the context of those that currently have a diagnosis. In addition to mental health disorders (e.g., depression and anxiety), this review also includes constructs relating to symptoms underlying other mental health conditions (e.g., fibromyalgia and ADHD). According to the WHO, mental health conditions is a term that broadly captures 'mental disorders, psychosocial disabilities and other mental states associated with significant distress, impairment in functioning, or risk of self-harm' (WHO, 2022). Other potential synonyms to mental health conditions could be mental illness and mental ill health. However, for this review, we will use mental health conditions to capture mental disorders and other psychosocial disabilities and mental states more broadly.	The measures that were considered related to mental health conditions include: **The Liebowitz Social Anxiety Scale-Self-report** [LSAS-SR], **Subjective Units of Discomfort** [SUDS], **Beck Anxiety Inventory** [BAI], **The Short Health Anxiety Inventory** [SHAI], **Beck Depression Inventory II** [BDI-II], the **Revised Fibromyalgia Impact Questionnaire** [FIQR], the **Conners' Adult ADHD Rating Scale-Investigator Rated: Screening Version** [CAARS-INV: SCV], and **The Depression Anxiety Stress Scale** [DASS-21]. The **LSAS-SR** aims to assess the extent to which respondents fear and avoid particular situations e.g., 'using a telephone in public'. The **SUDS** asked respondents to rate their current anxiety from 'no anxiety to 'very severe anxiety, the worse ever encountered'. The **BAI** asks about specific anxiety symptoms e.g. 'numbness or tingling'. The **SHAI** includes items (e.g., 'worry about health') to assess symptoms of health anxiety. The **BDI-II** asks respondents about feeling 'sadness', 'pessimism', 'past failure', 'loss of interest', etc. as potential symptoms of depression. The **FIQR** aims to evaluate the extent to which symptoms of fibromyalgia make it difficult to complete certain tasks such as 'prepare a homemade meal' and also asks about a range of symptoms related to 'pain' and 'energy' and 'memory problems' and 'anxiety'. This questionnaire also asks participants about the extent to which symptoms prevent accomplishing goals and to what extent they are perceived as overwhelming [Bennett et al., 2009]. The **CAARS-INV: SV** aims to assess symptoms of ADHD such as 'loses things necessary for tasks or activities' and 'appears restless inside even when sitting still'. The **DASS-21** includes items such as: 'I found it hard to wind down'. The **CES-D** aims to measure a range of depressive symptoms such as 'I was bothered by things that usually don't bother me' and 'I did not feel like eating; my appetite was poor'.
Mental health and well-being on a continuum		
Mental languishing	In contrast to mental health conditions, which affects a proportion of the population, mental health concerns the entire population; falls on a complex continuum; and is fundamental human right for all (Patel et al., 2018). Mental health refers to the capacity of thought, emotion, and behaviour that allows an individual to realise their own potential in life, to cope with day-to-day stressors, and to positively contribute to their society (WHO, 2022; Patel et al. 2018). In an effort to capture mental health constructs related to symptoms of mental health (e.g., depression, anxiety) across a wider distribution of the population (that have not met clinical thresholds) and related constructs (e.g., stress and burnout), we	The measures that were considered related to mental languishing: include **The State-Trait Anxiety Inventory** [STAI], **The Hamilton Rating Scale for Depression** [HAMD], **The Edinburgh Postnatal Depression Scale** [EPDS], the **Patient Health Questionnaire** [PHQ-9], **Symptoms of Anxiety-Depression Index** [SAD-4], the **Generalized Anxiety Disorder** [GAD-7], the **Quick Inventory of Depressive Symptomatology** [QIDS_SR16], the **Center for Epidemiological Studies Depression Scale** [CES-D], the **PANAS-SF**, **The Perceived Stress Scale** [PSS or PSS-10], the **Maslach Burnout Inventory-Educators Survey** [MBI-ES], and the **Kessler Psychological Distress Scale [K10]**. The **STAI** asks respondents to describe themselves (e.g., 'I worry too much

	have included this broader construct of mental languishing. Stress can exacerbate mental health symptoms and vice versa. Stress is the feeling of being overwhelmed or experiencing emotional pressure. Burnout is a distinct construct but can be viewed as a sub-category of stress that is driven by the need to live a meaningful life. Burnout has been recognized by the WHO as a psychosocial risk syndrome resulting from situations of chronic work stress that have not been successfully managed, and includes emotional exhaustion (EE), i.e., feelings of energy depletion; depersonalization (DE), i.e., negative feelings towards the recipients of the work; and reduced personal accomplishment (PA), i.e., sense of failure. Therefore, burnout includes not only a crisis in values but also a disability (Maslach et al., 1996).	over something that really doesn't matter') to measure state and trait anxiety, with higher scores indicative of higher levels of anxiety. The **HAMD** looked at specific symptoms of depression (e.g., 'depressed mood', 'feelings of guilt', 'suicide', 'insomnia' etc.). The **EPDS** includes items such as 'In the past seven days, I have felt sad or miserable'. The **PHQ-9** includes items such as: 'Over the last two weeks, how often have you been bothered by little interest or pleasure in doing things?' The **SAD-4** includes two items about anxiety symptoms (e.g., 'feeling blue and hopeless') and two items about depressive symptoms (e.g., 'feeling tense and restless'). The **GAD-7** aims to assess symptoms of anxiety such as 'feeling nervous, anxious, or on edge'. The **QIDS_SR16** is a clinician-rated questionnaire for symptoms of depression, and it looks at a range of symptoms such as 'insomnia', 'mood', 'concentration' and 'suicide ideation'. The **CES-D** aims to measure a range of depressive symptoms such as 'I was bothered by things that usually don't bother me' and 'I did not feel like eating; my appetite was poor'. The **PANAS-SF** focused on the negative items, relating to mental health symptoms, which ask respondents about the extent to which they feel 'irritable', 'ashamed', distressed'. The **PSS/PSS-10** includes items such as 'in the last month, how often have you felt that you were unable to control the important things in your life? **The MBI-ES** includes items such as 'I feel emotionally drained from my work'. The **K10** included items such as: "In the past four weeks, how often did you feel nervous?"

Well-being	According to the WHO, well-being is experienced by individuals and societies and can be determined by macro-level factors (e.g., social, economic, and environmental conditions). According to this definition, well-being includes quality of life and the ability for individuals and societies to feel a sense of purpose and meaning in life. Well-being is a multidimensional construct and it covers both the state and the healthy functioning that enables people to live and lead fulfilling lives (Feldman & Kuyken, 2019). The conception of well-being is not limited to psychological and emotional well-being as it also relates to physical, social, and environmental well-being. Like mental health, well-being also falls on a continuum and concerns the entire population. Therefore, well-being (like mental health) does not exclude those that have a mental health condition. Individuals with a mental health condition experience mental health and well-being across this continuum (from low to high) (WHO, 2022; Westerhof & Keyes, 2010).	The measures that were considered to be related to well-being include the **Positive States of Mind** [PSOM], the **Subjective Authentic-Durable Happiness Scale** [SA-DHS], the **Warwick-Edinburgh Mental Well-being Scale** [WEMWBS], **The Mental Health Continuum-Short Form** [MHC-SF], the mental health items of the **Medical Outcomes Studies Short-Form General Health Survey** [MOS SF-36], the **World Health Organisation Quality of Life (Bref)** questionnaire [WHOQOL-BREF]. The **PSOM** scale includes items about satisfying states of mind including: focused attention ('feeling able to attend to a task you want or need to, without many distractions from within yourself'), productivity ('feeling of being able to stay at work until a task is finished, do something new to solve problems, or express yourself creatively'), responsible caretaking ('feeling that you are doing what you should do to take care of yourself or someone else'), restful repose ('feeling relaxed, without distractions or excessive tension'), sensuous nonsexual pleasure ('being able to enjoy bodily senses, enjoyable intellectual activity, doing things you ordinarily like, such as listening to music, enjoying the outdoors, lounging in a hot bath'), and sharing ('being able to commune with others in an empathetic, close way as in talking, walking, going out, or just being together'). The **MHC-SF** includes items about emotional (e.g., 'during the past month, how often do you feel satisfied with your life?'), psychological (e.g., 'during the past month, how often do you feel that you had experiences that challenged you to grow and to become a better person?'), and social (e.g., 'during the past month, how often do you feel that you had something important to contribute to society') well-being. The **SA-DHS** asks respondents about their regular level of items such as 'overall well-being', 'happiness' and 'pleasure.' The **MOS SF-26** assesses physical functioning, physical role functioning, bodily pain, general health, vitality, social functioning, emotional role functioning, and mental health. For the purpose of the review, we have focused on the mental health and vitality items, with higher scores related to a more favourable health status [Ware et al., 1993]. The mental health items relate to general mood or affect, including depression, anxiety, and psychological well-being. The **WEMWBS** includes items such as 'I am feeling optimistic about the future' and 'I've been feeling very useful'. The **WHOQOL-BREF** includes items about physical (e.g., 'to what extent do you feel that physical pain prevents you from doing what you need to do?'), psychological (e.g., 'how much do you enjoy life?'), social (e.g., 'how satisfied are you with your personal relationships?'), and environmental (e.g., 'how safe do you feel in your daily life?') quality of life and includes an overall health ('how satisfied are you with your health?') and quality of life ('how would you rate your quality of life?') item.'

This table includes the name of the category for the proposed mechanisms [(1) mindfulness skills; (2) attention and awareness; (3) attitudes of mindfulness; (4) decentering; (5) dysfunctional thoughts, attitudes, and behaviours; and (6) emotional regulation] and outcomes [(1) mental health conditions; (2) mental languishing; (3) well-being]. Operational definitions for each category along with a list of the measures, and item examples are included.

References

Baer, R. A., Smith, G. T., Lykins, E., Button, D., Krietemeyer, J., Sauer, S., Walsh, E., Duggan, D., & Williams, J. M. G. (2008). Construct Validity of the Five Facet Mindfulness Questionnaire in Meditating and Nonmeditating Samples. *Assessment*, *15*(3), 329–342. https://doi.org/10.1177/1073191107313003

Feldman, C., & Kuyken, W. (2019). *Mindfulness: Ancient wisdom meets modern psychology*. The Guilford Press.

Fresco, D. M., Moore, M. T., van Dulmen, M. H. M., Segal, Z. V., Ma, S. H., Teasdale, J. D., & Williams, J. M. G. (2007). Initial Psychometric Properties of the Experiences Questionnaire: Validation of a Self-Report Measure of Decentering. *Behavior Therapy*, *38*(3), 234–246. https://doi.org/10.1016/j.beth.2006.08.003

Greco, L. A., Baer, R. A., & Smith, G. T. (2011). Assessing mindfulness in children and adolescents: Development and validation of the Child and Adolescent Mindfulness Measure (CAMM). *Psychological Assessment*, *23*(3), 606.

Maslach, C. (1996). Maslach burnout inventory-human services survey (MBI-HSS). *MBI Manual*, 192–198.

Patel, V., Saxena, S., Lund, C., Thornicroft, G., Baingana, F., Bolton, P., Chisholm, D., Collins, P. Y., Cooper, J. L., Eaton, J., Herrman, H., Herzallah, M. M., Huang, Y., Jordans, M. J. D., Kleinman, A., Medina-Mora, M. E., Morgan, E., Niaz, U., Omigbodun, O., … UnÜtzer, Jü. (2018). The Lancet Commission on global mental health and sustainable development. *The Lancet*, *392*(10157), 1553–1598. https://doi.org/10.1016/S0140-6736(18)31612-X

Segal, Z., Williams, M., & Teasdale, J. (2018). *Mindfulness-Based Cognitive Therapy for Depression, Second Edition*. Guilford Publications.

Shapiro, S. L., Carlson, L. E., Astin, J. A., & Freedman, B. (2006). Mechanisms of mindfulness. *Journal of Clinical Psychology*, *62*(3), 373–386. https://doi.org/10.1002/jclp.20237

Thompson, R. A. (1991). Emotional regulation and emotional development. *Educational Psychology Review*, *3*(4), 269–307. https://doi.org/10.1007/BF01319934

Westerhof, G. J., & Keyes, C. L. M. (2010). Mental Illness and Mental Health: The Two Continua Model Across the Lifespan. *Journal of Adult Development*, *17*(2), 110–119. https://doi.org/10.1007/s10804-009-9082-y

World Health Organization. (2021). *Depression: Overview*. https://www.who.int/news-room/fact-sheets/detail/depression

World Health Organization. (2022a). *Mental Disorders*.

World Health Organization. (2022b). *Mental health: Strengthening our response*. https://www.who.int/news-room/fact-sheets/detail/mental-health-strengthening-our-response

Supplement 11. Operational definitions and examples of mental health strategies.

Intervention type	Definition	Operationalization	Example
Treatment	Interventions offered to a subgroup of the population currently suffering from a recognized disorder	(1) Participants identified as currently suffering from a diagnosable condition<br>(2) Primary aim of intervention is to reduce current symptoms	Participants with a formal diagnosis of depression taking part in a mindfulness programme aimed at reducing current depressive symptoms.
Prevention (indicated)	Interventions offered to targeted subgroups of the population that are already experiencing some level of difficulty but do not currently have a formal diagnosis.	(1) Participants identified as already experiencing some level of difficulty<br>(2) Primary aim of intervention is to prevent the development or onset of disorder	Participants who have reported lower mood compared to general population but do not currently have a formal diagnosis of depression who are taking part in a mindfulness programme with the aim of preventing depression
Prevention (selective)	Interventions offered to targeted subgroups of the population that are identified as being at an elevated risk for disorder but do not currently have diagnostic-level difficulties	(1) Participants identified as being at an elevated risk for disorder<br>(2) Primary aim of intervention is to prevent the development or onset of disorder	Participants without a diagnosis of depression but having a relevant risk-factor (e.g., having a parent with depression) taking part in a mindfulness programme with the aim of preventing depression
Prevention (universal)	Interventions offered to the general public or whole population groups with the aim of preventing onset of disorder	(1) Open sampling method and inclusion/exclusion criteria with participants from the general public or whole population groups<br>(2) Primary aim of intervention is to prevent the development or onset of disorder	Participants without a diagnosis of depression from the general population taking part in a mindfulness programme with the aim of preventing depression
Promotion	Interventions offered to the general public or whole population groups with the aim of promoting positive mental health or well-being	(1) Open sampling method and inclusion/exclusion criteria with participants from the general public or whole population groups<br>(2) Primary aim of intervention is to improve more positive mental health and well-being outcomes	Participants without a diagnosis of depression from the general population taking part in a mindfulness programme aimed at promoting subjective well-being and positive mental health

This table includes operational definitions of different types of mental health strategies (treatment, prevention [universal, selective, indicated], and promotion) based on previous work the Institute of Medicine (1994) (Munoz et al., 1996) report and other works (Arango et al., 2018; Greenberg & Abenavoli, 2017; Huppert & So, 2013; World Health Organization, 2002)

Referenes

Arango, C., Díaz-Caneja, C. M., McGorry, P. D., Rapoport, J., Sommer, I. E., Vorstman, J. A., McDaid, D., Marín, O., Serrano-Drozdowskyj, E., Freedman, R., & Carpenter, W. (2018). Preventive strategies for mental health. *The Lancet Psychiatry*, *5*(7), 591–604. https://doi.org/10.1016/S2215-0366(18)30057-9

Greenberg, M. T., & Abenavoli, R. (2017). Universal Interventions: Fully Exploring Their Impacts and Potential to Produce Population-Level Impacts. *Journal of Research on Educational Effectiveness*, *10*(1), 40–67. https://doi.org/10.1080/19345747.2016.1246632

Huppert, F. A., & So, T. T. C. (2013). Flourishing Across Europe: Application of a New Conceptual Framework for Defining Well-Being. *Social Indicators Research*, *110*(3), 837–861. https://doi.org/10.1007/s11205-011-9966-7

Muñoz, R. F., Mrazek, P. J., & Haggerty, R. J. (1996). Institute of Medicine report on prevention of mental disorders: Summary and commentary. *American Psychologist*, *51*, 1116–1122. https://doi.org/10.1037/0003-066X.51.11.1116

World Health Organization. (2002). *Prevention and promotion in mental health* (What Is Prevention and Promotion in Mental Health?).

Supplement 12. Quality assessment of included studies.

Quality criteria	Reporting of mediated effect	Plausibility or coherence	Timeline	Gradient
Cladder-Micus et al. (2018)	+	+	-	-
Van Aalderen et al. (2012)	+	+	-	/
Collado-Navarro et al. (2021)	+	/	+	-
Lopez-del-Hoyo et al. (2022)	+	+	+	-
Dambrun et al. (2019)	/	+	-	-
Dimidjian et al. (2023)	+	+	+	-
Fissler et al. (2016)	-	+	-	-
Geurts et al. (2021)	/	+	+	-
Goldin et al. (2016)	/	/	-	-
Morrison et al. (2019)	/	+	+	-
Hayes-Skelton & Lee (2020)	/	+	-	-
Hofheinz et al. (2020)	/	/	+	-
Hoge et al. (2015)	/	+	-	-
Lengacher et al. (2014)	-	/	-	-
Lengacher et al. (2021)	-	+	+	-
Li et al. (2023)	/	+	+	-
Lonnberg et al. (2020)	+	+	-	+
Maloney et al. (2023)	+	+	+	+
McManus et al. (2012)	/	+	-	-
Moeira et al. (2023)[a]	+	/	-	-
Moeira et al. (2023)[b]	/	/	-	-
Montero-Marin et al. (2021)	+	+	-	+
Montero-Marin et al. (under review)	+	+	/	+
Nyklicek et al. (2014)	/	/	-	+
Perez-Aranda et al. (2019)	+	+	+	/
Sousa et al. (2022)	+	+	-	-
Spinhoven et al. (2022)	+	+	+	-
Sun et al. (2022)	+	+	-	-
van der Velden et al. (under review)	/	/	+	-
Villa & Hilt (2014)	+	/	-	-
**Overall quality rating (scores 0-30)**	21	25.5	12.5	6
**Key:** ● = High (scores 21-30) ● = Intermediate (scores 11-20) ● = Low (scores 0-10)				

This table includes a quality assessment of the included records based on meeting key criteria informed by Alsubaie et al., 2017, Kazdin, 2007, and Zhao et al., 2010. To address the reporting of the mediated effect, first column indicates the extent to which the included record reported: 1) the significance of direct effect and 2) the sign of *path c'* (total effect) or *path a x b* and *path c* independently. To address the plausibility or coherence, second column indicates the extent to which the included record met the following conditions: 1) the paper uses theory to articulate the mechanism through which the programme is hypothesized to work, and 2) the hypotheses about the mechanisms were

articulated and operationalized. The timeline criterion (third column) was addresses by examining the extent to which the included records used different time-point assessments to examine the proposed mediator and outcome. The gradient criterion (fourth column) was met if the record establishes 1) a relationship between the amount of practice and the proposed mediator and 2) a relationship between the amount of practice and outcome. For all criteria, a '+' was allocated if the criterion was fully met, whereas a '/' was allocated if the criterion was partially met and a '-' if the criterion was not fully met. A total quality rating score was calculated by subtracting the '/' (0.5), and '-' (1.0) from the maximum score of 30 (+1 point for each study). Scores that ranged between 0-10 were interpreted as 'Low'; 11-20 as 'Intermediate'; and 21-30 as 'High.'

References

Alsubaie, M., Abbott, R., Dunn, B., Dickens, C., Keil, T. F., Henley, W., & Kuyken, W. (2017). Mechanisms of action in mindfulness-based cognitive therapy (MBCT) and mindfulness-based stress reduction (MBSR) in people with physical and/or psychological conditions: A systematic review. *Clinical Psychology Review*, *55*, 74–91. https://doi.org/10.1016/j.cpr.2017.04.008

Kazdin, A. E. (2007). Mediators and Mechanisms of Change in Psychotherapy Research. *Annual Review of Clinical Psychology*, *3*(1), 1–27. https://doi.org/10.1146/annurev.clinpsy.3.022806.091432

Zhao, X., Lynch, J. G., Jr., & Chen, Q. (2010). Reconsidering Baron and Kenny: Myths and Truths about Mediation Analysis. *Journal of Consumer Research*, *37*(2), 197–206. https://doi.org/10.1086/651257

Supplement 13. Description of included randomized controlled trials (RCTs) with active comparator(s).

	Sample (N)	*Mental health strategy*	*MBP/P*	*Mindfulness condition (n)*	*MBP protocol*	*Dose*	*Delivery*	*Active comparator (n)*	*Mediators Tested*	*Outcomes Tested*	*Data analytic method*	*Key findings*	*Additional findings*	*Notes*
Cladder-Micus et al. (2018)	Full or partial remitters for MDD<br><br>PP: 115<br>M: 115<br><br>[sub-sample of van Aalderen et al. (2012)]	Prevention (indicated, selective)	MBP	MBCT + TAU (63)	MBCT D	Eight weekly group sessions (2.5 hr each) + 6 hr retreat day + daily home practice (30-45 min)	Psychiatrist or therapist with MBCT training	TAU [WL + standard mental healthcare] (52)	**LEIDS-R**	**HAMD**, BDI	Univariate path analysis with non-parametric bootstrapping based on 1,000 bootstrap samples (with 95% CI)	A sig. negative IE of group (MBCT+TAU vs TAU) on HAMD through LEIDS-R and a sig. negative direct effect [**complementary mediation**].	Similar effects for BDI.	All proposed mediators were tested. Change scores were used for mediator and outcomes.
Van Aalderen et al. (2012)	Full or partial remitters for MDD<br><br>ITT: 219<br>M: 205<br>[MBCT + TAU = 102; TAU = 103]	Prevention (indicated, selective)	MBP	MBCT + TAU (111)	MBCT D	Eight weekly group sessions (2.5 hr each) + 6 hr retreat + daily home practice (30-45 min)	Psychiatrist or therapist with MBCT training	TAU [WL + standard mental healthcare] (108)	**RSS, PSWQ, KIMS [accept without judgement subscale]**	**HAMD**	Multivariate and univariate path analysis with bootstrapping based on 5,000 bootstrap samples (with 95% CI)	In univariate model, sig. negative IEs were reported for group (MBCT + TAU vs TAU) on HAMD through RSS, PSWQ, and KIMS [accept without judgement], with a sig. negative direct effect [**complementary mediation**].	In multivariate model, the total IE was significant and negative, when all mediators were included in model, but the direct effect was non-sig. and negative. RSS and PSWQ made sig. independent contributions, but KIMS [accept without judgement] did not.	Mediation analyses were only conducted for variables whereby there was a sig. relationship between outcomes and proposed mediators. Change scores used for mediators and DV used post-scores controlling for pre-scores. Paths a, b, and c were not clearly reported in text.
Collado-Navarro et al. (2021)	Individuals with depressive, anxious, or	Treatment	MBP	MBSR +TAU (30)	MBSR	Eight weekly group sessions (2	Clinical psychologist with MBSR training	ABCT + TAU (30) or **TAU alone**	**FFMQ, SCS**	**DASS-21 [total score** and anxiety, depression,	Univariate maximum likelihood-based path	A sig. negative IE of group (MBSR+TAU vs. TAU) on	A sig. negative IE of group (MBSR+TAU vs. TAU) on	No differences were found between

	adjustment disorders<br><br>ITT: 90<br>M: 60<br>[MBSR + TAU = 30, TAU alone = 30]					hr each) + daily home practice (30 min)		[usual psychological/psychiatric treatment] (30)		and stress subscales]	analysis with bootstrapping based on 10,000 bootstrap samples (with 95% CI)	DASS-21 total score through FFMQ and SCS and non-sig. negative direct effect [**indirect-only mediation**].	DASS-21 subscale scores through FFMQ and SCS and a non-sig. negative direct effect.	MBSR + TAU and ABCT + TAU on primary outcome and therefore the mediation analyses did not compare these groups. All proposed mediators were tested. Change scores for mediators and outcomes were used.
Lopez-del-Hoyo et al. (2022)	Individuals with depressive, anxious, or adjustment disorders<br><br>ITT: 90<br>M: 60<br>[MBSR + TAU = 30, TAU alone = 30]<br><br>[same sample as Collado-Navarro et al. (2021)]	Treatment	MBP	MBSR + TAU (30)	MBSR	Eight weekly group sessions (2 hr each) + daily home practice (30 min)	Clinical psychologist with MBSR training	ABCT + TAU (30) or **TAU alone** [usual psychological/psychiatric treatment] (30)	**FFMQ [acting with awareness subscale], SCS [self-kindness, common humanity, and mindfulness subscales]**	**DASS-21**	Univariate maximum likelihood path analysis with bootstrapping based on 10,000 bootstrap samples (with 95% CI)	A sig. negative IE for group (MBSR + TAU vs. TAU) on DASS-21 through self-kindness, common humanity, and mindfulness subscales of SCS and non-sig. negative direct effect [**indirect-only mediation**]. Non-sig. negative IE for group on DASS-21 through acting with awareness subscale (FFMQ) and non-sig negative direct effect [**no effect (non mediation)**].	The total effect between the condition and outcome was significant [p = .034].	Mediation analyses were only conducted for variables where there was a sig. relationship between outcomes and proposed mediators. Within-group mediation analyses were not reported to supplement non-sig. IE. Change scores for mediators and outcomes were used.
Dambrun et al. (2019)	Psychology students<br><br>ITT: 89*<br>M: 89*	Promotion	P	BS (31)	MBCT/MBSR	One-off practice (21 min)	Experimenter	Relaxing music (32) or active	**B/SP, Harmony, Emotional stability,**	**SA-DHS**	Multivariate and univariate path analysis with bootstrapping	In the univariate analyses, a non-sig. positive IE of group (BS vs relaxing music	In the multivariate analyses, a sig. positive IE of group (BS vs	The direct effect was not always reported. Mediation

								listening (25)	**mind wanderin g,** UC		based on 5,000 bootstrap samples (with 95% CI)	and active listening) on SA-DHS through mind-wandering was found (with no direct effect reported).	relaxing music and active listening) on SA-DHS through B/SP and harmony [with UC included in model] and a non-sig. positive direct effect. Non-sig. positive IE and direct effect of group on SA-DHS through emotional stability (when entered into the mediation model with UC and harmony). In the univariate model, a sig. positive IE of group on SA-DHS through UC with positive non-sig. direct effect [**indirect-only mediation**].	analyses were only conducted for variables where there was a sig. relationship between the independent and dependent variable and between the independent variable and proposed mediator. Within-group mediation analyses were not reported to supplement non-sig. IE. Unclear if change scores are used.
Dimidjian et al. (2023)	Individuals with a history of MDD with mild depressive symptoms (361)<br><br>Secondary analysis of Segal et al. (2020)<br><br>ITT: 460<br>M: 361	Prevention (indicated/selective)	MBP	Mindful Mood Balance (MMB) (164)	MBCT	12-week intervention with 8 sessions + 45 min orientation + two 10 min check-in calls and emails	Self-help & online	Usual Depression Care (UDC) (197)	**EQ, FFMQ, RRS**	**Depressive relapse (PHQ-9)**	Causal mediation model using natural IE	A positive sig. natural IE of group (MMB vs UDC) on PHQ-9 through EQ, FFMQ, and RRS. Non-sig. positive direct effects were reported [**indirect-only**].	Sensitivity analyses demonstrated a significant natural IE after handling missing data using multiple imputations.	All proposed mediators were tested.
Fissler et al. (2016)	Participants with MDD<br><br>ITT: 74	Treatment	MBP	Truncated MBCT (38)	MBCT D	Three weekly one-on-one sessions (1.5 hr each)	Trained clinical psychologist	Resting/relaxation (36)	**MAIA [attention regulation, self-regulatio**	**BDI-II**	Multivariate and univariate path analysis with bootstrapping	In the univariate model, a sig. IE of group (MBCT vs. resting/relaxatio	In the multivariate model, positive sig. IE of group on BDI-II	The direct effect was not always reported and the signs

	CC: 68<br>M: 68<br>[MBCT = 36, Resting/relaxation = 32]					+ daily home practice (25 min)			**n, body listening, and trusting subscales], EQ [decentering subscale]**		based on 1,000 bootstrap samples (with 95% CI)	n) on BDI-II through EQ in isolation, but direct effect is not reported. Non-sig. IEs of group on BDI-II through MAIA scales [attention regulation, self-regulation, body listening, and trusting], but direct effect is not reported.	through increased attention regulation, self-regulation, body listening, and trusting (MAIA) with decentering (EQ) included in the serial path model and sig. positive direct effect.	(negative or positive) for the IE were not always reported. Mediation analyses were only conducted for variables whereby there was a significant treatment effect. Change scores for mediators and outcome were used. Within-group mediation analyses were not reported to supplement non-sig. IE.
Geurts et al. (2021)	Adults with ADHD<br><br>PP: 120<br>M: 120<br><br>[secondary analysis of Janssen et al. (2019)]	Treatment/ Promotion	MBP	MBCT + TAU (60)	MBCT D	Eight weekly group sessions (2.5 hr each) + daily home practice (30 min)	Mindfulness teacher	TAU [usual treatments for ADHD patients] (60)	**FFMQ-SF** [**total score** and five subscales], **SCS-SF** [**total score** and positive vs negative subscales], **BRIEF-A** [**total score** and nine subscales], CAARS-INV: SV	**CAARS-INV:SV, MHC-SF**	Multivariate and univariate path analysis with bootstrapping based on 5,000 samples (with 95% CI)	In the univariate models: A non-sig. negative IE of group (MBCT+TAU vs TAU) on CAARS-INV: SV through FFMQ-SF total score and non-sig. positive IE for SCS-SF total score. A sig. negative IE of group on CAARS-INV: SV through the BRIEF-A total score. A sig. positive IE of group on MHC-SF through SCS-SF total scores but not for FFMQ-SF.	In the multivariate models: A non-sig negative total IE on CAARS-INV:SV through FFMQ-SF and SCS-SF total scores and subscale scores; A sig. positive total IE on MHC-SF through FFMQ-SF, SCS-SF, and CAARS-INV: SV total and subscale scores	No direct effects were reported. All proposed mediators were tested. Within-group mediation analyses were not reported to supplement non-sig. IE. Baseline measures of outcomes and mediators were used as covariates for change scores.

Goldin et al. (2016)	Participants with SAD<br><br>PP: 108<br>M: 108	Treatment	MBP	Adapted MBSR (36)	MBSR	Twelve weekly group sessions (2.5 hr each) + daily home practice	Mindfulness teacher (MBSR) & doctoral clinical psychologist (CBGT)	CBGT (36) or WL (36)	**ERQ [reappraisal frequency & self-efficacy subscales], SAFE, CD-Quest, FFMQ, ACS [attention focusing and shifting subscales], RRS [brooding subscale]**	**LSAS-SR**	Univariate path analysis with bootstrapping based on 5,000 bootstrap samples (with 95% CI)	A sig. positive IE of group (CBGT vs MBSR) on LSAS-SR through reappraisal self-efficacy (ERQ) and SAFE. There were non-sig. negative IEs of group on LSAS-SR through reappraisal frequency (ERQ), FFMQ, and ACS [attention focusing] and non-sig positive IEs for CD-Quest, ACS [shifting], and RRS [brooding].	N/A	No direct effects were reported. All proposed mediators were tested. Only comparisons between CBGT and MBSR are reported here. Within-group mediation analyses were not reported to supplement non-sig. IE. Residualized scores for mediators and outcome were used.
Morrison et al. (2019)*	Participants with SAD<br><br>[sub-sample of Goldin et al. (2016)]<br><br>CC: 81<br>M: 76<br>[for pre-post intervention (26 = CBGT, 22 = MBSR); for pre-follow-up (20 = CBGT, 17 = MBSR)]	Treatment	MBP	Adapted MBSR (26)	MBSR	Twelve weekly group sessions (2.5 hr each) + daily home practice	Mindfulness teacher (MBSR) & doctoral clinical psychologist (CBGT)	CBGT (27) or WL (28)	**Positive affective empathy**	**LSAS-SR**	Univariate path analysis with bootstrapping based on 10,000 bootstrap samples (with 95% CI) following the Preacher & Hayes (2008) method	A sig. negative IE for group (CBGT vs MBSR) on LSAS-SR through positive affective empathy pre-post intervention.	A sig. negative IE for group (CBGT vs MBSR) on LSAS-SR through positive affective empathy pre-follow-up.	No direct effects were reported. Only positive affective empathy was evaluated due to sig. treatment effects between MBSR and WL. Only comparisons between CBGT and MBSR are reported here. Residualized scores for mediators and outcome were used.
Hayes-Skelton & Lee (2020)	University students and staff with SAD	Treatment	P	Mindfulness of emotio	MBCTD	One-off practice (10 min)	Experimenter	Attentional control (15) or CR (15)	**EQ [decentering subscale],**	**SUDS,** Willingness	Univariate path analysis with bootstrapping	A sig. positive IE for group (mindfulness condition vs.	A non-sig. IE for group (mindfulness condition vs.	No direct effects were reported. Only

	ITT: 46<br>M: 46			ns practic e (16)					**TMS [decenteri ng subscale]**		(with 95% CI and SE)	attentional control) on SUDS through TMS. A non-sig. IE for group on SUDS through EQ.	attentional control) on willingness through TMS. A non-sig. IE for group on willingness through EQ.	comparisons between mindfulness and attentional control are reported here. Mediation analyses were only conducted for variables where there was a significant relationship between the proposed mediator and outcome. Within-group mediation analyses were not reported to supplement non-sig. IE. Residualized change scores for mediators and outcome were used.
Hofheinz et al. (2020)	Participant s with current MDD episode<br><br>ITT: 72<br>PP: 50 [CI = 23, MI = 27]<br>M: n/a	Treatment	MBP	MI (33)	MBCT D [first two sessions]	Two group therapy sessions (1.5 hr each) + daily home practice	CBT therapist or doctoral candidate with CBT experience	Cognitive interventi on (CI) (39)	**ATQ-R [negative self-statement s, well-being, and self-confidenc e subscales] , DAS [perform ance evaluatio n and approval by others subscales]**	**BDI-II**	Multi-level structural equation modelling (MSEM) [univariate mediation model] (with 95% CI)	A sig. positive IE for group (cognitive intervention vs MI) on BDI-II through negative self-statements (ATQ-R). A non-sig. negative IE for group on BDI-II through well-being, and self-confidence (ATQ-R) and non-sig. positive IE for performance	N/A	No direct effects were reported. All proposed mediators were tested. Within-group mediation analyses were only conducted by time.

												evaluation and approval by others (DAS).		
Hoge et al. (2015)	Participants with GAD<br><br>CC: 38<br>M: 38<br><br>[secondary analysis of Hoge et al. (2013)]	Treatment	MBP	Adapted MBSR (19)	MBSR	Eight weekly group sessions + 4 hr retreat day + daily home practice (20 min)	Mindfulness teacher	SME (19)	**EQ [decentering subscale], FFMQ** [**total score** and five subscales]	**BAI**, PSWQ-PW	Multivariate and univariate path analysis with bootstrapping based on 4,000 samples (with 95% CI)	In the univariate model, sig. positive IEs for group (MBSR vs SME) on BAI through EQ [decentering] and FFMQ [total score].	In the multivariate model, a sig. positive total IE for group on BAI through FFMQ [total score] and EQ [decentering], but only EQ made an independent contribution.	No direct effects were reported. Results for subscale scores are not reported here. Within-group mediation analyses were not reported to supplement non-sig. IE. Change scores were used.
Lengacher et al. (2014)	Post-treatment breast cancer survivors<br><br>ITT: 84<br>CC: 82<br>M: 82<br>[MBSR(BC) = 40, UC = 42]<br><br>[secondary analysis of Lengacher et al. (2013)]	Prevention (selective)/ Promotion	MBP	MBSR (BC) (41)	MBSR	Six weekly group sessions (2 hr each) + daily home practice (15 to 45 min) six days/week	MBSR trained psychologist	WL + usual care [regular doctor visits] (43)	**CARS [fear of recurrence problems and fear of recurrence concerns]**, CES-D, MOS SF-36 [energy]	**CES-D, STAI [sate and trait anxiety], PSS, MOS SF-36 [mental health]**	Estimated IEs [univariate mediation model] using Sobel first-order test (z-scores)	Non-sig. positive estimates of IEs for group on CES-D, STAI (trait anxiety), and MOS SF-36 [mental health] through CRS [fear of recurrence problems]. Sig. positive estimates of IEs for group (MBSR (BC) vs. WL + usual care) on PSS and STAI (state anxiety) through CRS (fear of recurrence problems). Non-sig positive estimates of IEs for group on PSS, CES-D, STAI [state and trait anxiety], and MOS SF-36 (mental health) through CRS	Non-sig. positive estimates of IEs of group on PSS, STAI [state and trait anxiety] and MOS SF-36 [mental health] through CES-D. Non-sig. positive estimates of IEs of group on PSS, CES-D, STAI [state and trait anxiety] and MOS SF-26 through MOS SF-26 [energy].	The signs (negative or positive) of the estimates of IE are based on z-scores. No direct effects are reported. Mediation analyses were only conducted for variables whereby there were sig. treatment effects and sig. relationships between the proposed mediator and outcome. Within-group mediation analyses were not reported to supplement non-sig. IE. Change

												(fear of recurrence concerns).		scores for mediators were used.
Lengacher et al. (2021)	Post-treatment breast cancer survivors<br><br>ITT: 322*<br>CC: 299 [MBSR (BC) = 152, UC = 147]<br>M: 291-295<br><br>[secondary analysis of Lengacher et al. 2016]	Prevention (selective)	MBP	MBSR (BC) (167)	MBSR	Six weekly group sessions (2 hr each) + daily home practice (15 to 45 min) six days/week	MBSR trained psychologist	WL + usual care [regular doctor visits] (155)	**CARS [fear of recurrence problems],** PSS	**STAI [sate anxiety]**	Univariate path analysis with bias-corrected bootstrapping (with 95% CI)	Sig. IE of group (MBSR (BC) vs WL + usual care) on STAI [state anxiety] through CRS [fear of recurrence problems] at 6 wks.	Sig. IE of group on STAI [state anxiety] through CRS [fear of recurrence problems] at 12 wks. Sig. IE of group on STAI [state anxiety] through PSS at 6 wks and 12 wks.	The signs (positive or negative) and point estimate of IEs were not reported. No direct effects were reported. Mediation analyses were only conducted for variables where there were significant main effects. Change scores for mediators and outcomes were used
Li et al. (2023)	Individuals with emotional disorders with moderate emotional distress (75)<br><br>ITT: 75	Treatment/ Prevention (selective)	MBP	iMIED + TAU (37)	MBSR	49 days with 30 min of practice and content daily	Self-help & online	TAU only (medicinal and psychological treatment)	**FFMQ, AAQ-II**	**K10, BAI, BDI-II**	Path analysis with 95% CIs	A non-sig. IE of group (iMIED+. TAU vs TAU only) on K10, BAI, and BDI-II through FFMQ and AAQ-II was found when considered independently.	Sig. IE of group (iMEID + TAU vs TAU) on BAI & BD-II when FFMQ and AAQ-II were entered sequentially, with non-sig. direct effects [indirect-only]. Sig. IE of group on K10 through FFMQ and AAQ-II sequentially with sig. direct effect [complementary mediation]	All proposed mediators were tested in mediation models. No direct effects reported when mediators were reported independently.
Lonnberg et al. (2020)	Pregnant women at-risk of	Prevention (indicated, selective) /	MBP	MBCP (96)	MBCT/ MBSR	Eight weekly group	Mindfulness teacher	Lamaze childbirth class (97)	**FFMQ [total score** and	**EPDS, PSS, PSOM**	Univariate path analysis with	A sig. positive IE for group on EPDS through	A sig. positive IE for group on PSS through	No direct effects were reported for

	perinatal depression<br><br>ITT: 193<br>Completers: 164<br>M: 164<br>[MBCP = 75, Lamaze = 89]	Promotion				sessions (2 hr 15 min each) + daily home practice (30 min) + reunion			five subscales]		bootstrapping based on 10,000 bootstrap samples (with 95% CI)	FFMQ, but non-sig. positive direct effect [**indirect-only mediation**]. A sig. positive IE for group (MBCP vs Lamaze) on PSS through FFMQ, but a non-sig. positive direct effect for group on PSS [**indirect-only mediation**]. A sig. negative IE and direct effect for group on PSOM through FFMQ [**complementary mediation**]	non-reactivity and non-judgement (FFMQ) subscales, but not for observing, acting with awareness, and describing subscales (FFMQ).<br>A sig. positive IE for group on EPDS through non-judgement subscale, but non-sig. positive IE for non-reactivity, observing, acting with awareness, and describing subscales (FFMQ). Non-sig. negative IE for group on PSOM through the five subscales of FFMQ	subscale scores. All proposed mediators were tested. Within-group mediation analyses were not reported to supplement non-sig. IEs. Change scores for mediators and outcomes were used.
Maloney et al. (2023)	Graduates of MBSR/MBCT (164)<br><br>Secondary analysis of Maloney et al. (2024)<br><br>ITT: 164	Promotion/ Prevention (universal)	MBP	MBCT-TiF (83)	MBCT	135 min / week for 12 weeks + home practice for 30-45 min/day	Teacher-led	Ongoing mindfulness practice (OMP) (81)	**FFMQ-SF, EQ, SCS-SF**	**WEMWBS, WHOQOL BREF (Psychological), PHQ-9, GAD-7**	Path analysis with 95% CIs (10,000 bootstrapped samples)	Sig. positive IE of group (MBCT-TiF vs OMP) on WEMWBS through FFMQ-SF, SF, and EQ with positive direct effect; A sig negative IE of group on PHQ-9 through FFMQ-SF, EQ, and SCS-SF with negative sig. direct effects [**complementary mediation**]; Sig. negative IE of group on	Reverse mediation models whereby the outcomes are tested as mediators and vice versa are provided in supplementary material.	All proposed mediators were tested.

												GAD-7 and positive IE of group on WHOQOLBREF (psychological) through EQ and FFMQ with sig. direct effects (negative and positive, respectively)[**complementary mediation**], but non-sig. IE of group on outcome through SCS-SF, with sig. direct effects [signs same as above] [**direct-only**].		
McManus et al. (2012)	Participants with hypochondriasis<br><br>ITT: 74<br>M: 74 for ITT and 68 for PP [36 = US, 32 = MBCT]	Treatment	MBP	MBCT + US (36)	MBCT D	Eight weekly group sessions (2 hr each) + daily home practice (1 hr)	Mindfulness teacher + CBT therapist	US [WL + encouragement to continue medication or interventions during study period] (38)	**FFMQ**	**SHAI**	Univariate path analysis with non-parametric bootstrapping based on 5,000 bootstrap samples (with 95% CI) using z-statistic	A sig. negative estimate for IE for group (MBCT vs US) on SHAI through FFMQ, but non-sig. direct effect.	N/A	The signs (positive or negative) of indirect effects were based on z-scores and were not reported for direct effects. All proposed mediators were tested. Change scores for mediators and outcomes were used.
Moreira et al. (2023)[a]	Women with deep endometriosis with current pain symptoms (63)	Promotion/ Prevention (selective)	MBP	Brief mindfulness based intervention (bMBI) (26)	MBSR	4 weekly sessions (1.5 hr per session) + 20-30 min of practice per day	Teacher-led	Standard medical care (SMC) [hormonal therapy & analgesics] (28)	MPQ [pain and **affective dimensions**], PSS	**MOS-SF-36 (vitality subscale), PSS**	Path analysis (95% CIs with 2,000 bootstrapped samples)	Sig. negative IE of group (bMBI vs SMC) on PSS through MPQ [pain affective dimension], with negative non-sig. direct effect [**indirect-**	There was also a sig. IE of group (bMBI vs SMC) on PSS through MPQ [pain sensory dimension] with non-sig. direct effect. When MPQ [pain	Only variables that were significant pre-post intervention were used in mediation model. Sensitivity

	Secondary analysis of Moreira et al (2022)<br><br>ITT: 63<br>M: 54											**only**]; Positive Non-sig. IE of group on MOS-SF-36 (vitality) through MPQ [affective dimension], with positive sig. direct effect of group on MOST-SF-36 (vitality) [**direct-only**].	sensory and affective dimensions] and PSS were considered sequentially, there was a sig. IE of group on MOS-SF-36 (vitality) with sig. direct effect.	analyses were also conducted. Baseline measures of proposed mediators and outcomes were considered as covariates.
Moreira et al. (2023)[b]	Women with deep endometriosis with current pain symptoms (54)<br><br>Secondary analysis of Moreira et al (2022)<br><br>ITT: 63<br>M: 54	Promotion	MBP	Brief mindfulness based intervention (bMBI) (26)	MBSR	4 weekly sessions (1.5 hr per session) + 20-30 min of practice per day	Teacher-led	Standard medical care (SMC) [hormonal therapy & analgesics] (28)	PC, **PA, NA**	**MOS-SF-36 (mental health subscale),** Pain numeric rating scale (PNRS),	Path analysis (95% CIs with 2,000 bootstrapped samples	A sig. IE of group (bMBI vs SMC) on MOS-SF-36 (mental health) through PA and NA.	A sig. IE of group (bMBI vs SMC) on PNRS through PC, but not PA and NA. A sig. IE of group on MOS-SF-36 (mental health) through PC when not controlled for by PA. Additional serial mediation effects are reported in text.	Baseline measures of mediators and outcomes were considered as covariates. Sensitivity analyses were also conducted. Direct effects not clearly reported for when mediators are considered independently.
Montero-Marin et al. (2021)	Secondary-school teachers<br><br>CC: 166<br>M: 166<br><br>[secondary analysis of Crane et al. (2020)]	Promotion/ Prevention (universal)	MBP	M-FP [instructor-led format] (86)	MBCTD	Eight sessions (in groups or self-led) + daily home practice (20 min)	Mindfulness teacher	M-FP [self-led format] (80)	**FFMQ-SF, SCS-SF**	**WEMWBS, PSS, PHQ-9, GAD-7, MBI-ES**	Univariate path analysis with unstandardized path estimates based on 20,000 bootstrap samples (with 95% CI)	A sig. positive IE for group (M-FP instructor-led vs. self-led) on WEMWBS through FFMQ-SF and non-sig. positive direct effect [**indirect-only mediation**]. A sig. negative IE for group on PSS through FFMQ-SF and a sig. negative direct effect [**complementar**	N/A	All proposed mediators were tested variables were tested. Change scores for mediators and outcomes were used.

												**y mediation**]. A sig. negative IE for group on PHQ-9, GAD-7 and MBI-ES through FFMQ-SF with non-sig. negative direct effects [**indirect-only mediation**]. A sig. positive IE for group on WEMWBS through SCS-SF with a non-sig. positive direct effect [**indirect-only mediation**]. A sig. negative IE for group on PHQ-9 through SCS-SF with also a sig. negative direct effect [**complementary mediation**]. A sig. negative IE for group on PSS, GAD-7 and MBI-ES through SCS-SF with non-sig negative direct effect [**indirect-only mediation**].		
Montero-Marin et al. (under review)	Adults with a history of MDD in full or partial remission on ADM	Prevention (indicated, selective)	MBP	MBCT + ADM (212)	MBCT	8 weekly sessions (2.25 hr per session) + four booster sessions	Teacher-led	ADM + standard monitoring in primary care settings (212)	**FFMQ**	**BDI-II**	A moderated mediation model (growth mixture) 95% CIs	A sig. negative IE of group (MBCT + ADM vs ADM) on BDI-II trough FFMQ, moderated by latent profile of higher depressive symptom severity at baseline, was found. A	Results were maintained after adjusting for potential confounders (e.g., amount of home-based practice and actual discontinuation of ADM)	All proposed mediators were tested.

												negative non sig. direct effect was found for group on BDI-II [**indirect-only**]		
Nyklicek et al. (2014)	Individuals who have had a percutaneous coronary intervention<br><br>ITT: 114<br>CC: 107<br>[MBSR = 55, self-help = 52]<br>M: 107	Prevention (selective)/ Promotion	MBP	Adapted MBSR (57)	MBSR	Three weekly group sessions (1.5 to 2 hr each) + daily home practice (30 min)	Clinical psychologist	Minimal mindfulness self-help (57)	**FMI-s**	**SAD-4, PSS, WHOQOL-Bref**	Univariate path analysis with non-parametric bootstrapping based on 5,000 bootstrap samples	A sig. positive IE for group (MBSR vs self-help) on SAD-4 through FMI-s in the subsample younger than 60 years old. A sig positive IE for group on WHOQOL-BREF through FMI-s in the whole sample. Non-sig. negative IE for group on PSS through FMI-s in the younger subsample.	N/A	No direct effects were reported. Mediation analyses were only conducted for significant relationships between the independent, dependent, and mediator variables. Within-group mediation analyses were not reported to supplement non-sig. IEs. Unclear if change scores are used.
Perez-Aranda et al. (2019)	Participants with FM<br><br>ITT: 225<br>CC: 149<br>M: 149<br>[MBSR + TAU = 49, FibroQOL + TAU = 51, TAU = 49]	Treatment/ Prevention (selective)	MBP	MBSR + TAU (75)	MBSR	Eight weekly group sessions (2 hr each) + optional retreat + daily home practice	Mindfulness teacher	FibroQOL + TAU (75) or TAU alone (75)<br><br>[TAU involved WL + continuation of medication (if any)]	**FFMQ [act with awareness subscale], PIPS**	**FIQR, PSS-10,** PCS	Multivariate path analysis with bias-corrected bootstrapping (with 95% CI and SE)	A sig. negative IE for group (MBSR + TAU vs TAU) on FIQR through act with awareness (FFMQ) and PIPs with non-sig negative direct effects [**indirect-only mediation**]. A sig. negative IE for group on PSS-10 through act with awareness (FFMQ) and PIPS with a non-sig. negative direct	A sig. negative IE for group on PCS through PIPS with a non-sig. negative direct effect.	Mediation analyses were only conducted for variables that demonstrated a significant association between proposed mediators and outcomes. Comparisons between MBSR + TAU and TAU were prioritized. Change scores for

												effect [**indirect-only mediation**].		mediators and outcomes were used. Total indirect effects in multivariate models do not seem to be reported.
Sousa et al. (2022)	University students<br><br>ITT: 43<br>CC: 40<br><br>[secondary analysis of Menezes de Sousa et al. (2021)]	Prevention (universal)	P	Body scan and breath awareness (20)	MBSR	Three sessions of one-off practice (30 min each)	Researcher	Attentional control [AC] (23)	**MAIA [total score]**	**STAI-S [state anxiety]**	Univariate path analysis with bootstrapping based on 10,000 bootstrap samples (with 95% CI)	A sig. negative IE of group (mindfulness practice vs. AC) on STAI-S through MAIA and a sig. negative direct effect [**complementary mediation**]	N/A	Only variables that sig. changed after training were included in mediation analysis. Change scores were used.
Spinhoven et al. (2022)	Individuals with treatment refractory anxiety disorders<br><br>ITT: 171<br>PP: 136<br>M: 171	Treatment	MBP	MBCT (82)	MBCT D	Eight weekly group sessions (2 hr each) + daily home practice (up to 1 hr/ day)	Mindfulness teacher	CBT-RP (89)	**DERS, FFMQ, PSWQ, RSS**	**BAI**	Cross-lagged structural equation models and univariate path analysis with bootstrapping based on 5,000 bootstrap samples (with 95% CI)	Non-sig. positive IEs for group (MBCT vs. CBT-RP) on BAI through FFMQ, PSWQ, and RSS but sig. positive direct effect [**direct-only mediation**]. Sig. positive IE for group (MBCT vs. CBT-RP) on BAI through DERS but non-sig positive direct effect [**indirect-only mediation**]	The IE of group on BAI through DERS was non-sig. based on p-values but was close to reaching statistical significance (p = .06).	Significance of IE is based on CIs and sig. of direct effect is based on p-values. Mediation analyses were only conducted for the primary outcome (BAI) but other outcome variables were measured. Within-group mediation analyses were not reported to supplement non-sig. IEs. Change scores do not seem to be used.

Sun et al. (2022)	Pregnant women at risk of perinatal depression<br><br>ITT: 168<br>M: 168<br><br>[secondary analysis of Sun et al. (2021)]	Prevention (indicated, selective)	MBP	Adapted app-based MBCT (84)	MBCTD	Eight weekly group sessions + daily home practice (15-25 min)	A psychologist with mindfulness experience	ACG [plus regular health consultation] (84)	**FFMQ**	**EPDS**	Univariate path analysis with bootstrapping based on 5,000 bootstrap samples (with 95% CI)	A sig. negative IE for group (app-based MBCT vs. ACG) on EPDS through FFMQ with a non-sig. negative direct effect [**indirect-only mediation**]	N/A	All proposed mediators were tested. Change scores may have been used.
van der Velden et al. (n.d. [pre-print])	Individuals with a history of recurrent depression who were partially or fully remitted<br><br>ITT: 80<br>PP: n/a<br>M: n/a	Prevention (indicated, selective)	MBP	MBCT + TAU (50)	MBCTD	Eight weekly group sessions (2.25 hr each) + daily home practice	MBCT therapists	TAU [either a stable dose of antidepressant medication or no medication] (30)	**EQ [decentering subscale], FFMQ, MAIA [body listening, attention regulation]**	**QIDS_SR16**	Univariate path analysis with bootstrapping based on 5,000 bootstrap samples (with 95% CI)	Sig. positive IEs for group (MBCT + TAU vs TAU) on QIDS_SR16 through EQ [decentering] and FFMQ, with non-sig. direct effect [**indirect-only mediation**]. Non-sig. IEs for group on QIDS-SR16 through MAIA (body listening and attention regulation] with no direct effect reported.	N/A	Mediation analyses were conducted for variables that predicted change in outcome. Signs of point estimates for IEs and direct effects were not always reported. Within-group mediation analyses were not reported to supplement non-sig. IEs. Change scores were used for mediators.
Villa & Hilt (2014)	Non-clinical sample of undergraduate students<br><br>ITT: 114<br>*CC: 111<br>M: 111	Prevention (universal)	P	Breath meditation (38)	MBSR	One-off mindfulness practice (8 min)	Experimenter	Somatic relaxation (37) or no-treatment (36)	**SR**	**PANAS-SF [negative affect subscale]**	Univariate moderated-mediation path analysis with bootstrapping based on 5,000 bootstrap samples (with 95% CI)	A sig. positive IE for group (mindfulness condition vs. somatic relaxation) on PANAS-SF (negative affect) through SR for women but not for men, with a non-sig.	N/A	Mediation analyses were informed by significant 3x2x2 ANCOVA tests. Only comparisons between breath meditation

												negative direct effect [**indirect-only mediation**].		and somatic relaxation were used for mediation analyses. Change scores were used.

This table summarizes the main characteristics of the included randomised controlled trials (RCTs) with active comparators. Note: N = number of studies, MBP = mindfulness-based programme, P = practice, M = mediation, MBCT = mindfulness-based cognitive therapy, TAU = treatment-as-usual, MBCTD = mindfulness-based cognitive therapy for depression, WL = waitlist, LEIDS-R = the leiden index of depression sensitivity revised, HAMD = The hamilton rating scale for depression, BDI = Beck Depression Inventory, ITT = intention-to-treat, RSS = rumination on sadness scale, PSWQ = penn state worry questionnaire-past week, KIMS = kentucky inventory of mindfulness skills, MBSR = mindfulness-based stress reduction, ABCT = attachment-based compassion therapy, FFMQ = five facet mindfulness questionnaire, SCS = self-compassion scale, DASS-21 = depression anxiety stress scales-21, BS = body scan, BS/P = body/self-phenomenology, UC = unified consciousness, SA-DHS = subjective authentic-durable happiness Scale, MMB = mindful mood balance, UDC = usual depression care, EQ = experiences questionnaire, RRS = ruminative response scale, PHQ-9 = patient health questionnaire, CC = complete cases, MAIA = multidimensional assessment of interoceptive awareness, ADHD = attention deficit hyperactivity disorder, FFMQ-SF = five facet mindfulness questionnaire short form, SCS-SF = self-compassion scale short form, BRIEF-A = behavior rating inventory of executive function – adult version, CAARS-INV:SV = conner's adult ADHS rating scale – investigator rated screening version, CBGT = cognitive behaviour group therapy, ERQ = emotional regulation questionnaire, SAFE = subtle avoidance frequency examination, CD-Quest = the cognitive distortions questionnaire, ACS = attentional control scale, LSAS-SR = leibowitz social anxiety scale – self-report, SAD = social anxiety disorder, CR = cognitive restructuring, SUDS = subjective units of discomfort, MI = mindfulness intervention, CBT = cognitive behaviour therapy, ATQ-R = automatic thoughts questionnaire, DAS = dysfunctional attitude scale, GAD = generalised anxiety disorder, SME = stress management education, BAI = beck anxiety inventory, MBSR(BC) = mindfulness-based stress reduction (breast cancer), CARS = concerns about recurrence scale, CES-D = center for epidemiological studies depression scale, MOSSF-36 = medical outcomes studies short form, STAI = state-trait anxiety inventory, PSS = perceived stress scale, iMIED = internet-based mindfulness intervention for emotional disorders, AAQ-II = acceptance and action questionnaire, K10 = kessler psychological distress scale, MBCP = mindfulness-based childbirth and parenting, EPDS = Edinburgh postnatal depression scale, PSOM = positive states of mind, MBCT-TiF = mindfulness-based cognitive therapy – taking it further, OMP = ongoing mindfulness practice, US = unrestricted services, SHAI = short health anxiety inventory, bMBI = brief mindfulness-based intervention, SMC = standard medical care, PC = pain catastrophising, PA = positive affect, NA = negative affect, MPQ = mcgill pain questionnaire, M-FP = mindfulness-based cognitive therapy - finding peace in a frantic world, MBI -ES = Maslach burnout inventory – educators survey, ADM = antidepressant medication, FMIs = freiburg mindfulness inventory, SAD-4 = symptoms of anxiety depression index, `FibroQOL = fibromyalgia quality of life, FIQR = the revised fibromyalgia impact questionnaire, AC = attentional control, CBT-RP = cognitive behaviour therapy relapse prevention, DERS = difficulties in emotion regulation scale, ACG = attention control group, QIDS_SR16 = quick inventory of depressive symptomatology, SR = state rumination. MHC-SF = mental health continuum-short form; PIPS = the psychological inflexibility in pain scale;TMS = toronto mindfulness scale, WEMWBS = warwick-edinburgh mental well-Being scale; WHOQOL-Bref = world health organisation-bref. **Other notes:** The five subscales of FFMQ and FFMQ-SF include observing, describing, acting with awareness, non-judging, and non-reactivity. The six subscales of SCS and SCS-SF include self-kindness (self-judgement), common humanity (isolation), and mindfulness (over-identification). The four subscales of the KIMs include the observing, describing, act with awareness, and accept without judgement subscales. The nine subscales of the BRIEF-A include: inhibit, shift, emotional control, self-monitor, initiate, working memory, plan/organize, task monitor, and organization of materials. For Dambrun et al. (2019), one participant dropped out after randomisation but the text does not specify which group and this is why the numbers for the conditions do not add to the total sample. For the Lengacher et al. (2021) study, two participants did not complete baseline levels (322 vs 320 participants). Univariate models or simple mediation analyses are prioritized in the key findings column to help increase clarity. Records that are blocked out in grey represent papers that share the same sample or a subsample of another included paper.

Supplement 14. Refined recommendations for testing and reporting mediation.

Aligned with the recommendations outlined by Zhao et al. (2010) and Kazdin (2007) [see Supplement 7], we outline below additional considerations for testing and reporting mediation. The first step is to calculate and report the significance and coefficients of the IE (path ab). The significance of the IE should be established using an appropriate data-analytic method (e.g., path analysis, SEM) and the confidence intervals (CIs) of the coefficients of the IE should be calculated and reported based on methods that account for the non-normal distribution of the standard error (e.g., bootstrapping, Monte Carlo, etc.). Reporting CIs, instead of p-values, for the IE will help overcome issues related to the non-normal distribution of the standard error of the IE (MacKinnon et al., 2002). The sample size calculation and the analytical strategy used to test the IE should be clearly stated. The sample size needs to be calculated according to a statistical power estimation specifically aimed at evaluating the IE, and this must be adjusted for potential multiple comparisons in the case we are testing different potential mechanisms using the same sample. Whether or not the analytic approach to test the IE was the primary or secondary analysis is key since this has important implications in terms of the statistical power estimation. In terms of analytical strategies, a per-protocol approach is often recommended to assess the targeted mechanism due to the intervention. The per-protocol analysis focuses on individuals who adhere to the intervention protocol as intended, providing insight into the specific effects of the intervention on the targeted mechanism. By analysing only those who comply with the intervention (e.g., MBP), this approach can help identify whether changes in the mechanism are indeed a result of the intervention itself (by means of receiving the appropriate dose needed to produce effects) rather than other factors (Hernán & Robins, 2017). For testing the IE, sensitivity analyses can also be considered (VanderWeele, 2016) to help control other potential confounders of the mediator-outcome relationship, especially due to the possibility of common causes for change in the mediator and outcome (Loeys et al., 2015). In this sense, testing the IE between-group in RCTs, whereby the condition (e.g., MBP) is compared to a control group, should be prioritized over within-group mediation to help establish mechanism specificity. However, the within-group mediation results should supplement the between-group mediation findings in the case of a non-significant IE, to further understand whether the non-significant IE is occurring because the control group also works through the same mediator, or because there are no IEs within the condition (e.g., MBP) or control group alone or in both. All theoretically proposed mechanisms, evaluated in the study, should be tested rather than basing this decision on the three-step Baron and Kenny approach (e.g., significant main effects or significant correlations between changes in proposed mediators and outcomes), as this will avoid losing sight of the different types of mediation that can occur and therefore provide rich information to guide subsequent research. Once the significance and the coefficients of the IE have been calculated

and reported, the next step is to calculate the significance and coefficients of the direct effect (path c), using p-values to calculate the significance (e.g., Wald test). This information will then help classify the type of mediation, in accordance with the framework established by Zhao and colleagues (2010). The direct effect (path c) should be clearly defined as examining the relationship between the condition and outcome, whilst controlling for the proposed mediators. The direct effect should be distinguished from the total effect (path c'), which is examining the relationship between the condition and outcome without controlling for the mediators

References:

Hernán, M. A., & Robins, J. M. (2017). Per-protocol analyses of pragmatic trials. *N Engl J Med*, *377*(14), 1391–1398.

Kazdin, A. E. (2007). Mediators and Mechanisms of Change in Psychotherapy Research. *Annual Review of Clinical Psychology*, *3*(1), 1–27. https://doi.org/10.1146/annurev.clinpsy.3.022806.091432

Loeys, T., Moerkerke, B., & Vansteelandt, S. (2015). A cautionary note on the power of the test for the indirect effect in mediation analysis. *Frontiers in Psychology*, *5*. https://www.frontiersin.org/articles/10.3389/fpsyg.2014.01549

MacKinnon, D. P., Lockwood, C. M., Hoffman, J. M., West, S. G., & Sheets, V. (2002). A comparison of methods to test mediation and other intervening variable effects. *Psychological Methods*, *7*(1), 83–104. https://doi.org/10.1037/1082-989X.7.1.83

VanderWeele, T. J. (2016). Mediation Analysis: A Practitioner's Guide. *Annual Review of Public Health*, *37*(1), 17–32. https://doi.org/10.1146/annurev-publhealth-032315-021402

Zhao, X., Lynch, J. G., Jr., & Chen, Q. (2010). Reconsidering Baron and Kenny: Myths and Truths about Mediation Analysis. *Journal of Consumer Research*, *37*(2), 197–206. https://doi.org/10.1086/651257
